# Occurrence of *Chlamydiaceae* in Raptors and Crows in Switzerland

**DOI:** 10.3390/pathogens9090724

**Published:** 2020-09-02

**Authors:** Sandro Stalder, Hanna Marti, Nicole Borel, Konrad Sachse, Sarah Albini, Barbara Renate Vogler

**Affiliations:** 1 Vetsuisse Faculty, Institute for Food Safety and Hygiene, National Reference Center for Poultry and Rabbit Diseases, University of Zurich, 8057 Zurich, Switzerland; sarah.albini@vetbakt.uzh.ch (S.A.); bvogler@vetbakt.uzh.ch (B.R.V.); 2 Vetsuisse Faculty, Institute of Veterinary Pathology, University of Zurich, 8057 Zurich, Switzerland; hanna.marti@uzh.ch (H.M.); n.borel@access.uzh.ch (N.B.); 3 Institute of Bioinformatics, Friedrich-Schiller-Universität Jena, 07743 Jena, Germany; konrad.sachse@gmx.net

**Keywords:** *Chlamydiaceae*, raptors, crows, Switzerland, *C.psittaci*/*C.abortus* intermediates

## Abstract

Bacteria of the family *Chlamydiaceae* are globally disseminated and able to infect many bird species. So far, 11 species of *Chlamydia* have been detected in wild birds, and several studies found chlamydial strains classified as genetically intermediate between *Chlamydia* (*C*.) *psittaci* and *C.* *abortus*. Recently, a group of these intermediate strains was shown to form a separate species, i.e., *C.* *buteonis*. In the present study, 1128 samples from 341 raptors of 16 bird species and 253 corvids representing six species were examined using a stepwise diagnostic approach. *Chlamydiaceae* DNA was detected in 23.7% of the corvids and 5.9% of the raptors. In corvids, the most frequently detected *Chlamydia* species was *C.* *psittaci* of outer membrane protein A (*ompA*) genotype 1V, which is known to have a host preference for corvids. The most frequently detected *ompA* genotype in raptors was M56. Furthermore, one of the raptors harbored *C.* *psittaci* 1V, and two others carried genotype A. *C.* *buteonis* was not detected in the bird population investigated, so it remains unknown whether this species occurs in Switzerland. The infection rate of *Chlamydiaceae* in corvids was high compared to rates reported in other wild bird species, but neither *Chlamydiaceae*-positive corvids nor raptors showed overt signs of disease. Since the *Chlamydiaceae* of both, raptors and crows were identified as *C.* *psittaci* and all *C.* *psittaci* genotypes are considered to be zoonotic, it can be suggested that raptors and crows pose a potential hazard to the health of their handlers.

## 1. Introduction

Microorganisms of the family *Chlamydiaceae* are Gram-negative, obligate intracellular bacteria characterized by their unique biphasic lifecycle [1]. The family *Chlamydiaceae* currently comprises a single genus, *Chlamydia*, including 14 characterized species [2,3,4]. *Chlamydiaceae* are globally disseminated and have a broad host range including mammals, birds, reptiles, and amphibians [2]. *Chlamydia* (*C*). *psittaci*, the best-known chlamydial species associated with birds, has been reported to infect more than 460 avian species comprising at least 30 orders [5]. Wild birds serve as an important reservoir not only for *C. psittaci* but also for several other chlamydial species. To date, eleven *Chlamydia* species have been detected in birds [3,6,7,8,9,10,11].

Avian chlamydiosis caused by *C. psittaci* is a notifiable disease in Switzerland and other countries. Between 2010 and 2019, 46 cases were reported to the Federal Food Safety and Veterinary Office, of which 35 cases occurred in domestic and eleven in wild birds [12]. The clinical signs in infected birds can be variable, depending on the virulence of the strain, the susceptibility of the host species, and the immune status of the individual [1,13]. Shedding of the bacteria occurs in both diseased birds and asymptomatic carriers and can be intermittently activated by stressful events like migration, breeding or illness [14].

The zoonotic risk associated with *C. psittaci* and *C. abortus* infections is well-known for other chlamydial species harbored by birds; zoonotic transmission is suspected (e.g., *C. gallinacea* [15]) or unknown (e.g., *C. pecorum* [2], *C. buteonis* [3], *C. avium* [16]).

There are few studies on infection rates of *Chlamydiaceae* in birds in Switzerland but no study concerning raptors and crows. One study focusing on *C. psittaci* in pigeons, songbirds, and waterfowl found infection rates of 14.3%, 0.4%, and 4.3%, respectively [17]. Mattmann et al. (2019) investigated *Chlamydiaceae* infection rates in pigeons from different geographical areas in Switzerland and found a total infection rate of 16.9% [18]. 

In some European countries, however, the infection rates of *Chlamydiaceae* in raptors have been investigated. In Sweden, one study reported a *C. psittaci* infection rate of 1.3% in peregrine falcons (*Falco peregrinus*) and white-tailed sea eagles (*Haliaeetus albicilla*) using real-time PCR (qPCR) [19]. Gerbermann and Korbel (1993) reported *a C. psittaci* infection rate of 13.2% in raptors from southern Germany by antigen ELISA, whereas in eastern Germany Schettler et al. (2003) found 74.4% of the sampled raptors to be positive for *C. psittaci* using nested PCR [20,21]. Data on *Chlamydiaceae* in corvids from Europe appears to be even scarcer. One study from Poland found an infection rate of 13.4% based on qPCR, while in Italy an infection rate of 28.9% has been reported in corvids using nested PCR [8,10].

Several studies investigating wild birds found chlamydial species that could not be classified but were identified as genetic intermediates between *C. psittaci* and *C. abortus* [22,23,24,25,26,27,28,29,30]. One of these intermediates had initially been detected in a red-tailed hawk (*Buteo jamaicensis*) in the 1990s [31]. At that time, the organism was identified as *C. psittaci*. The genome of this isolate was later re-evaluated and recently classified as the new species *C. buteonis*, together with a new isolate found in a red-shouldered hawk (*Buteo lineatus*) [3,32]. The clinical importance of *C. buteonis* is still unknown as few studies have focused on clinical signs associated with chlamydial infections in raptors. However, both the red-tailed hawk and the red-shouldered hawk from which *C. buteonis* was isolated showed clinical signs of avian chlamydiosis, respiratory distress, and diarrhea in the first, and conjunctivitis in the latter. 

The aims of the present study were (i) the collection of data on the infection rates of *Chlamydiaceae* in raptors and corvids in Switzerland also related to a potential health hazard to humans and (ii) the characterization of the chlamydial species involved, with particular interest in the aforementioned, so far not fully characterized “intermediates” and the new species *C. buteonis* in view of the limited information available for these organisms.

## 2. Results

### 2.1. Chlamydiaceae 23S rRNA qPCR

#### 2.1.1. Species

Results of qPCR testing using an assay targeting the 23S ribosomal RNA (rRNA) gene of *Chlamydiaceae* (“*Chlamydiaceae* 23S rRNA qPCR”) for the different bird species are presented in Table 1. In total, 119 samples (10.5%) from 80 birds (13.5%) were positive for *Chlamydiaceae.* In corvids, *Chlamydiaceae* were detected in 60/253 animals (23.7%), while 20/341 raptors (5.9%) were positive. The odds ratio showed that the odds of *Chlamydiaceae* infection was five times higher in corvids than in raptors (OR = 4.99 (95% confidence interval (CI): 2.92–8.53), *p* < 0.01). *Chlamydiaceae* were detected in representatives of six raptor species, namely in 13/142 common buzzards (*Buteo buteo*), 3/32 Eurasian sparrowhawks (*Accipiter nisus*), 1/23 red kites (*Milvus milvus*), 1/66 common kestrels (*Falco tinnunculus*), 1/17 long-eared owls (*Asio otus*), and 1/17 barn owls (*Tyto alba*). In corvids, 59/207 carrion crows (*Corvus corone*) and 1/3 rooks (*Corvus frugilegus*) were positive for *Chlamydiaceae*.

#### 2.1.2. Geographical Origin

*Chlamydiaceae*-positive birds were detected in nine Swiss cantons as shown in Table 2. There was a strong trend towards higher rates of *Chlamydiaceae* positivity in the cantons Zug (52.9%) and Zurich (24.2%) compared to the other cantons tested. The lowest infection rate of the cantons of which at least one bird was positive was found in Lucerne (1.7%).

#### 2.1.3. Swab Type

*Chlamydiaceae* were detected in 13.9% of the choanal (*n* = 72), 8.8% of the cloacal (*n* = 46), and 1.1% of the fecal (*n* = 1) swabs (Table 3). Paired choanal and cloacal swabs were available from 79 birds that tested positive for *Chlamydiaceae* in at least one site. Regarding these 79 birds, *Chlamydiaceae* were detected in both swabs in 39 (49.4%) birds, in choanal swabs only in 33 (41.8%) birds and in cloacal swabs only in 7 (8.9%) birds. Based on chi-squared test, successful detection of *Chlamydiaceae*-positive birds was significantly higher (*p* < 0.01) with choanal swabs, which detected 72/79 (91.1%) of the cases, compared to cloacal swabs, which only detected 46/79 cases (58.2%). No appropriate comparison with fecal swabs was possible due to the limited number of birds where all three swab types were available. 

### 2.2. C. Psittaci qPCR and C. Buteonis qPCR

Of the 119 *Chlamydiaceae*-positive samples, all were negative in *C. buteonis* species-specific qPCR, and two were positive in *C. psittaci*-specific qPCR. Both positives originated from raptors, one from a Eurasian sparrowhawk (Nr. 268C), the other one from a common buzzard (Nr. 683C). Both specimens were choanal swabs, and in both animals, the cloacal swab was negative using *Chlamydiaceae* 23S rRNA qPCR.

### 2.3. 16S rRNA Conventional PCR and Sequencing

Partial sequences of the 16S ribosomal RNA (278 bp) were successfully obtained from 74 samples of 55 individuals that met the requirements of being negative by both previously described species-specific qPCRs and having a mean cycle quantification (Cq) value <35 in the *Chlamydiaceae*-specific 23S rRNA qPCR, i.e., eight samples from six raptors and 66 samples from 49 crows (Table 4). Seven samples (Nr. 14C, 311K, 556C, 556K, 669K, 671C, 671K) from five raptors were identified as *C. psittaci* M56 (accession number: CP003795.1). The remaining sample (Nr. 566C), a choanal swab from a common buzzard, showed 100% sequence identity with two *C. abortus* strains, *C. abortus* 15-58d44 (LS974600.1) and *C. abortus* 15-58d/44 (KX870502.1), and with three *C. psittaci* isolates, *C. psittaci* nier_A97 (KX603686.1), *C. psittaci* nier_A101 (KX603687.1), and *C. psittaci* nier_A113 (KX603688.1). The 16S rRNA sequences obtained from all the 66 samples from corvids also showed the highest similarity with the sequences of these five strains with identities ranging between 95.6% and 100%. The amplified sequence was identical in these five strains.

The ten samples selected for 16S rRNA (1481 bp) conventional PCR originated from one Eurasian sparrowhawk (Nr. 268C), one common kestrel (Nr. 311K), one rook (Nr. 621C), two common buzzards (Nr. 556K, 566C), and five carrion crows (Nr. 565C, 746C, 769C, 814C, 972C). The results were very similar to those of the partial 16S rRNA PCR (Table 5). Two strains found in a common buzzard (Nr. 556K) and a common kestrel (Nr. 311K) showed high nucleotide identity with *C. psittaci* M56 with identities of 99.1% and 100%, respectively. The strains found in the five carrion crows (Nr. 565C, 746C, 769C, 814C, 972C) one rook (Nr. 621C), and one common buzzard (Nr. 566C) again showed high sequence similarity with the five aforementioned *C. psittaci* and *C. abortus* strains with identities ranging from 99.6% to 100%. The strain detected in the Eurasian sparrowhawk (Nr. 268C) showed high sequence identity (98%) with several *C. psittaci* and *C. abortus* strains, including *C. psittaci* Ful127 (CP033059.1), *C. abortus* 84/2334 (CP031646.1), *C. psittaci* GIMC 2005 (CP024451.1), and *C. psittaci* WC (CP003796.1). 

### 2.4. Outer Membrane Protein A (ompA) Genotyping

**Figure 1:**
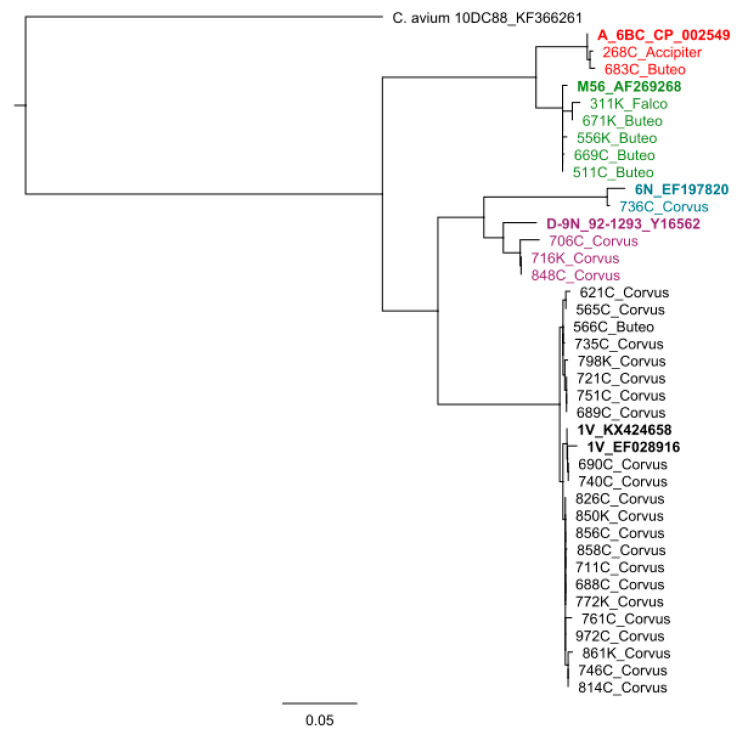
Outer membrane protein A (*ompA*) based Neighbor Joining dendrogram of *Chlamydiaceae* from raptors and corvids from Switzerland. Representative sequences from various *C. psittaci* genotypes are included in boldface. Designation of study isolates correspond to Table 6. Samples in the same color belong to the same *ompA* genotype.

Amplification and sequencing of the *ompA* gene was successful in both qPCR-positive samples for *C. psittaci*, as well as 33 selected samples that were positive for *Chlamydiaceae*, but negative in both species-specific qPCRs (Supplementary Table S1). The 33 samples were selected based on the mean Cq value in the *Chlamydiaceae* 23S rRNA qPCR, on host species, and geographical location. They originated from five common buzzards (Nr. 511C, 556K, 566C, 669C, 671K), one common kestrel (Nr. 311K), one long-eared owl (877K), one rook (621C), and 25 carrion crows (565C, 688C, 689C, 690C, 706C, 711C, 716K, 721C, 735C, 736C, 740C, 746C, 751C, 752C, 761C, 772K, 798K, 814C, 826C, 848C, 850K, 856C, 858C, 861K, 972C). Both organisms detected in the Eurasian sparrowhawk (Nr. 268C) and the common buzzard (Nr. 683C) positive by *C. psittaci* qPCR shared the highest *ompA* sequence identity with the strain *C. psittaci* Ful127 (CP033059.1) with identities of 99.9% and 99.5%, respectively. This strain had been detected in Northern fulmars (*Fulmarus glacialis*) from the Faroe Islands and belongs to *ompA* genotype A [33]. The *ompA* sequence of four common buzzards (Nr. 511C, 556K, 669C, 671K), the common kestrel (Nr. 311K), and the long-eared owl (Nr. 877K) shared the highest nucleotide identity with *C. psittaci* M56 (LS974600.1) with identities ranging from 97.2% to 100%. The remaining common buzzard (Nr. 566C), as well as 22 corvids (Nr. 565C, 621C, 688C, 689C, 690C, 711C, 721C, 735C, 740C, 746C, 751C, 752C, 761C, 772K, 798K, 814C, 826C, 850K, 856C, 858C, 861K, 972C) harbored a chlamydial species that shared the highest *ompA* sequence identity with *C. abortus* strain 15-58d/44 (KX870484.1), *C. psittaci* isolate 15-58D/43 (KX424658.1), and *C. abortus* strain 15-58d44 (LS974600.1) with identities ranging from 99.2% to 100%. All three strains are classified within the *ompA* genotype 1V. The *ompA* sequence of the sample of one carrion crow (Nr. 736C) shared the highest sequence similarity with *C. psittaci* isolate nier_A113 (KX603696.1), *C. psittaci* isolate nier_A97 (KX603693.1), and *C. psittaci* isolate 6N (EF197829.1), all belonging to the *ompA* genotype 6N with identities of 100%, 99.8%, and 98.4%, respectively. Furthermore, chlamydial organisms sharing the highest *ompA* sequence similarity with *C. psittaci* NJ1 (CP003798.1), belonging to *ompA* genotype D, were detected in three carrion crows (Nr. 706C, 716K, 848C). Identities ranged from 96.8% to 97.1%. Results of the *ompA* sequencing are shown in Table 6. In Figure 1, an *ompA* based Neighbor Joining dendrogram is shown. Two obtained *ompA* sequences (752C, 877K) were not included in the dendrogram due to poor sequence quality.

## 3. Discussion

### 3.1. Corvids

The *Chlamydiaceae* infection rate found in this study (23.7%) is in accordance with the findings of Di Francesco et al. (2015), who detected *Chlamydiaceae* in 28.9% (*n* = 22) of the 76 corvids sampled [8]. This study suggests, that *C. psittaci* of ompA genotype 1V is widespread in the Swiss crow population. Genotypes 1V and 6N are considered to be intermediates between *C. psittaci* and *C. abortus*. [10,34,35]. Genotype D, which was detected in three carrion crows, has a known host preference for turkeys [36].

As all corvid samples were negative in the species-specific qPCR for the recently described species *C. buteonis*, it remains unknown whether this species is able to infect corvids or not.

### 3.2. Raptors

The *Chlamydiaceae* infection rate in raptors (5.9%) was towards the lower end of the wide range of infection rates (1.3–74.4%) reported in European raptors [19,20,21] and is in agreement with the findings of Konicek et al. (2016) [37]. Two studies performed in the neighboring country of Germany reported higher infection rates of 13.2% and 74.4% in the southern and eastern part of the country based on antigen ELISA and nested PCR, respectively [20,21]. However, the significance of the 74.4% has to be put in perspective as the authors only tested a small number of birds (*n* = 39) for *Chlamydiaceae* [19].

Regarding the three orders of raptors, no significant differences in the *Chlamydiaceae* infection rate could be observed, as reported earlier [19,30]. Although members of the Accipitriformes (7.9%) showed a considerably higher infection rate than members of the Falconiformes (1.4%), this difference proved statistically not significant (*p* = 0.050).

*C. psittaci* M56, which was identified in seven raptors, is considered a mammalian strain with a host preference for muskrats and hares [38]; none of these samples were identified as *C. psittaci*-positive using the *C. psittaci*-specific qPCR by Pantchev et al. (2009). This lack of coverage had already been noticed by other researchers (Sachse K., personal communication). These raptors presumably got infected through their prey, as this strain is usually found in mammals, and it was detected only in raptors in this study. The mean Cq value in the *Chlamydiaceae* qPCR in these birds was 29.1 with values ranging from 14.1–38.3, thus suggesting that the bacteria in samples with low Cq values may have been actively replicating, and the positive result was not only due to residual bacteria from infected prey. Further, *ompA* genotyping revealed that one common buzzard harbored *C. psittaci* genotype 1V, a *C. psittaci*/*C. abortus* intermediate that has a host preference for corvids [10,34,39]. As all samples were found negative for *C. buteonis* using species-specific qPCR, it remains unknown whether this recently described chlamydial species does occur in raptors in Switzerland.

### 3.3. Geographical Distribution

Due to a geographically uneven distribution of samples, owed to the nature of sample collection, it was decided to forego a statistical analysis of differences in infection rate between the cantons. However, the finding that birds from the canton of Zurich showed a higher infection rate compared to birds from Lucerne is supported by previous studies [17,18]. Zweifel et al. (2009) reported a *Chlamydiaceae* infection rate of 3.3% in feral pigeons from Lucerne and 41.7% in feral pigeons from Zurich [17]. Mattmann et al. (2019) found an infection rate of 17.4% in pigeons from Lucerne and 27.5% in pigeons from Zurich [18]. Mattmann et al. (2019) explained the difference between the infection rates of these two areas with the fact that culling of pigeons, as performed in Zurich, may lead to an increased contact rate of individual pigeons due to frequent restructuring of the population, and therefore, the transmission of pathogens might be increased [18]. In Lucerne on the other hand, different population management programs, including city lofts, are in use.

### 3.4. Swab Types

Swabs of the choana detected significantly more *Chlamydiaceae*-positive birds than cloacal swabs, which is in accordance with studies on farmed chickens, ducks, geese, pigeons, turkeys, and cockatiels [9,40,41]. One study investigating the pathogenicity of different *C. psittaci* strains in chickens found that the overall pharyngeal excretion was slightly higher than the cloacal excretion and that the intensity of excretion varies depending on the *C. psittaci* strain involved [42]. These findings suggest that the respiratory tract plays a major role in the infection and transmission of chlamydiae [43]. Although the present study highlights that choanal swabs have a higher sensitivity, some birds were negative in the choanal but positive in the cloacal swab as the site of shedding depends on the stage of infection. Thus, for clinical sampling, it can be suggested to use both choanal and cloacal swabs for detection of *Chlamydiaceae*, since both are relatively easily accessible.

### 3.5. Public Health Concerns

The *Chlamydiaceae* of raptors and crows in this study were all identified as *C. psittaci*, belonging to the *ompA* genotypes M56, A and 1V (raptors), and *ompA* genotypes 1V, 6N and D (crows). *C. psittaci* is the best characterized zoonotic species of the family *Chlamydiaceae,* and all genotypes are considered zoonotic, including *C. psittaci* M56 [44,45,46,47,48]. The reported case numbers of human psittacosis indicate that the disease is most likely underdiagnosed, due to lack of disease awareness among the general public and physicians [49,50,51,52]. 

Sporadic outbreaks of psittacosis are regularly reported in the literature. In 2002, there was a psittacosis outbreak in the Blue Mountains, New South Wales, Australia, with 95 suspected cases with community-acquired pneumonia [53]. From January to April 2013, 25 individuals from southern Sweden were diagnosed with psittacosis [54]. Wild birds were thought to be the source of infection in both outbreaks [53,55]. *C. psittaci* Ful127 is thought to be the responsible agent for a psittacosis outbreak with 174 human cases on the Faroe Islands in the 1930s [33]. Humans contracted *C. psittaci* while catching juvenile fulmars and preparing them for cooking [56].

These examples show that *C. psittaci* outbreaks are still a possible threat to human health. Humans with an increased risk for psittacosis include those coming into close contact with birds on a regular basis (e.g., workers in a zoo or in pet shops, veterinarians, veterinary assistants, and pet bird owners) [57,58,59,60,61,62]. These individuals should take appropriate safety and hygiene measures when handling wild birds. A study showed that bird handlers applying simple measures like wearing protective gloves and washing their hands after handling birds were less likely to get infected by *C. psittaci* [63].

## 4. Materials and Methods 

### 4.1. Samples

Sampling was performed between April 2018 and January 2020. A total of 1128 samples were collected from 594 birds representing 22 species belonging to four orders (Supplementary Table S2). In detail, 483 samples were collected from 253 corvids of six species and 645 samples from 341 raptors representing 16 species. Samples consisted of dry choanal (*n* = 519), cloacal (*n* = 520), and fecal (*n* = 89) swabs. Choanal and cloacal swabs were obtained from deceased birds (*n* = 528), whereas from living birds (*n* = 66) only fresh fecal material was sampled with swabs after defecation (Table 7). Twenty-three birds died or were euthanized during treatment; therefore, all three swab types were available from these birds. Paired choanal and cloacal swabs were available from 511 birds. For sampling, dry swabs (FLOQSwab^®^, Copan Flock Technologies, Brescia, Italy) were used and stored in cryovials at −80 °C until further processing. 

Dead birds or their samples were obtained from the bird rehabilitation center of the Swiss Ornithological Institute in Sempach, Lucerne, the Wildlife Rehabilitation Center Landshut, Utzenstorf, Berne, the Clinic for Zoo Animals, Exotic Pets and Wildlife, Vetsuisse Faculty, University of Zurich, the Berg am Irchel Bird of Prey Sanctuary, as well as from gamekeepers and local hunters of various cantons. In total, sampled birds originated from 19 Swiss cantons. Carcasses of birds of prey and Corvidae were found dead or were euthanized due to incurable trauma or disease. In addition, carcasses of corvids shot in the scope of cantonal population control programs to reduce the number of birds were available. All living birds were inpatients either at the bird rehabilitation center of the Swiss Ornithological Institute or at the Wildlife Rehabilitation Center Landshut, Utzenstorf, canton of Berne. For all species, the canton of origin and date of sampling were noted if available.

### 4.2. DNA Extraction

DNA of the choanal and cloacal swabs was extracted using a commercial kit (Genomic DNA from tissue, NucleoSpin^®^ Tissue from Macherey-Nagel, Düren, Germany) according to manufacturer’s instructions. For each extraction lot, a negative control was prepared by using “Buffer T1” instead of the sample. DNA of the fecal samples was extracted with the NucleoSpin^®^ Stool kit (Macherey-Nagel, Düren, Germany) according to the company recommendations. Quality (260/280 value) and quantity of extracted DNA was measured using a Nanodrop 2000c spectrophotometer (Thermo Fisher Scientific, Waltham, MA, USA). The extracted DNA was stored at −20 °C until further use.

### 4.3. Chlamydiaceae 23S rRNA qPCR

All samples (*n* = 1128) were analyzed with a 23S rRNA based *Chlamydiaceae* family-specific real-time PCR as described previously, modified to include an internal amplification control (eGFP) to control for inhibition [64,65,66]. The cycle conditions were 95 °C for 20 s, followed by 45 cycles of 95 °C for 3 s, and 60 °C for 30 s. Detailed information about all primers and probes used in this study are listed in Table 8. All samples were tested in duplicates. The cycle threshold was set at 0.1 in each run, and a sevenfold dilution series of *C. abortus* was included as a standard curve in each run. Molecular grade water was used as a negative control. Samples were interpreted as positive if the mean Cq value was <38. Samples with questionable results with Cq values >38 were retested in duplicates. Samples with inhibited amplification were retested undiluted and tenfold diluted, both in duplicates. Samples repeatedly showing a Cq value >38 were considered as positive.

### 4.4. C. Psittaci OmpA qPCR

All *Chlamydiaceae*-positive samples were subsequently tested with the *C. psittaci*-specific qPCR according to the protocol as described by Pantchev et al. (2009) including an internal amplification control [67,68]. The reaction mix contained 4 μL (<150 ng/μL) sample template, 1 μL eGFP template, 1x TaqMan Universal PCR MasterMix (Thermo Fisher Scientific, Waltham, MA, USA), 900 nM of the primers CppsOMP1-F and CppsOMP1-R, 200 nM probe CppsOMP1-S, 900 nM of the primers eGFP-1-F and eGFP 2-R, and 200 nM probe eGFP-HEX in a final volume of 25 μL. A negative control (aqua bidest.) and a positive control (synthesized oligonucleotide of the *ompA* gene of a *C. psittaci* field isolate “T0592/03, amazon parrot” (National Reference Centre for poultry and Rabbit Disease, University of Zurich); synthesized by Microsynth) were used in duplicates in each run [18].

### 4.5. C. Buteonis OxaA qPCR

The *C. buteonis*-specific qPCR was performed as previously described in all *Chlamydiaceae*-positive samples, modified to include an internal amplification control [3,68]. The reaction mix contained 4 μL sample template, 1 μL eGFP template, 12.5 μL TaqMan Universal PCR MasterMix (Thermo Fisher Scientific, Waltham, MA, USA), 600 nM of the primers RSHA-F and RSHA-R, 200 nM probe RSHA-P, 400 nM of the primers eGFP-1-F and eGFP-2-R, and 200 nM probe eGFP-HEX in a final volume of 25 μL. A negative control (aqua bidest.) and a positive control (DNA of *C. buteonis* RSHA, kindly provided by Karine Laroucau, ANSES, Maison-Alfort, France) were used in duplicates in each run.

### 4.6. 16S rRNA PCR and Sequencing

Samples negative by both previously described species-specific qPCRs and fulfilling the requirement of a mean Cq value <35 in the *Chlamydiaceae* 23S rRNA qPCR were subjected to the 16S rRNA conventional PCR as previously described [69], using the modified primers 16S IGF (short) and 16S IGR (short) [70] to amplify a partial sequence of 278 bp. Per sample, a 50 μL reaction mix was prepared, containing 5 μL (<150 ng/μL) sample template, 25 μL Red Taq Ready Mix (Merck KGaA, Darmstadt, Germany), and 300 nM of both the forward (16S IGF) and the reverse (16S IGR) primer. Cycling conditions were 95 °C for 5 min, followed by 40 cycles of 95 °C for 60 s, 65 °C for 60 s, 72 °C for 90 s, and a final extension of 72 °C for 10 min. 16S rRNA sequences generated in this study are available in GenBank under accession numbers MT423441–MT423514.

Ten samples were selected based on the result of the 16S (partial) sequencing, host species, geographical location, and mean Cq value in the *Chlamydiaceae* 23S rRNA qPCR and subjected to the near-full length 16S rRNA conventional PCR to amplify a sequence of 1481 bp [71]. The reaction mix was identical to the reaction mix described above, but instead of 16S IGF (short) and 16S IGR (short), the forward and reverse primers 16S-IGF [69] and 16S-B1 [72] were used, respectively. Cycling conditions were identical to those described above, with the only difference that the annealing temperature was set at 57.5 °C instead of 65 °C. The nearly complete 16S rRNA gene sequences are available in GenBank under accession numbers MT429304 and MT430892–MT430900.

Products from all conventional PCRs were purified using the QIAquick^®^ PCR Purification Kit (Qiagen, Hilden, Germany) according to the manufacturer’s instructions. Purified amplicons were Sanger sequenced by Microsynth. The obtained sequences were assembled and analyzed using the Geneious Prime software (version 2019.2.3, https://www.geneious.com) and compared against the NCBI database using the BLASTn tool (NCBI, https://blast.ncbi.nlm.nih.gov/).

### 4.7. OmpA Genotyping PCR

Per sample, a reaction mix with a final volume of 50 μL containing 25 μL REDTaq ReadyMix (Merck KGaA, Darmstadt, Germany), 200 nM of the primers ompA F (CTU) and ompA rev [73], and 3 μL sample template with a DNA concentration of 25 ng/μL was prepared. Cycling conditions were 10 min at 95 °C, followed by 35 cycles of 95 °C for 30 s, 49 °C for 30 s, 72 °C for 60 s, and a final elongation at 72 °C for 7 min [73]. If amplification resulted in weak bands, a modified cycling protocol with 40 cycles of 95 °C for 60 s, 49°C for 60 s, 72 °C for 90 s was used [18]. The *ompA* sequences obtained in this study are available in GenBank under accession numbers MT450242–MT450276. Analysis of *ompA* nucleotide sequences was conducted using Geneious version 10.2 (Biomatters Ltd., available from https://www.geneious.com). Multiple sequence alignments were handled using MAFFT v7.450 [74] using the Auto algorithm and scoring matrix: 200PAM/k = 2. Phylogenetic trees were reconstructed using RAxML v8 [75] with nucleotide model GTR GAMMA and the Rapid hill climbing algorithm.

### 4.8. Statistical Analysis

Statistical analyses were carried out using SPSS version 26 software. For differences in detection rate of *Chlamydiaceae* from different swab sites, the chi-squared test was performed. The value of *p* < 0.05 was considered statistically significant. 

### 4.9. Ethical Statement

All animal housing and sampling were conducted in strict accordance to the Swiss law of animal welfare. None of the birds were killed for this study. The birds of which choanal and cloacal swabs were taken were euthanized due to incurable trauma or disease prior to sampling.

## Figures and Tables

**Table 1:** Total number and percentage of raptors and corvids positive for *Chlamydiaceae* per species and number and percentage of chlamydial species identified.

Species Name	*Chlamydiaceae* qPCR Pos. (%)	Final Classification						
		*C. Abortus*/ *C. Psittaci* (%)	*C. Psittaci* M56 (%)	*C. Psittaci* A (%)	*C. Psittaci* 6N (%)	*C. Psittaci* 1V (%)	*C. Psittaci* D (%)	Not Further Identified (%)
Bearded vulture	0	0	0	0	0	0	0	0
Black kite	0	0	0	0	0	0	0	0
Common buzzard	13 (9.2%)	0	5 (38.5%)	1 (7.7%)	0	1 (7.7%)	0	6 (46.2%)
Eurasian sparrowhawk	3 (9.4%)	0	0	1 (33.3%)	0	0	0	2 (66.7%)
European honey-buzzard	0	0	0	0	0	0	0	0
Golden eagle	0	0	0	0	0	0	0	0
Montagu’s harrier	0	0	0	0	0	0	0	0
Northern goshawk	0	0	0	0	0	0	0	0
Red kite	1 (4.3%)	0	0	0	0	0	0	1 (100%)
**Accipitridae subtotal**	**17 (7.9%)**	**0**	**5 (29.4%)**	**2 (11.8%)**	**0**	**1 (5.9%)**	**0**	**9 (52.9%)**
Common kestrel	1 (1.5%)	0	1 (100%)	0	0	0	0	0
Eurasian hobby	0	0	0	0	0	0	0	0
Peregrine falcon	0	0	0	0	0	0	0	0
**Falconidae subtotal**	**1 (1.4%)**	**0**	**1 (100%)**	**0**	**0**	**0**	**0**	**0**
Eurasian eagle-owl	0	0	0	0	0	0	0	0
Long-eared owl	1 (5.9%)	0	1 (100%)	0	0	0	0	0
Tawny owl	0	0	0	0	0	0	0	0
**Strigidae subtotal**	**1 (2.6%)**	**0**	**1 (100%)**	**0**	**0**	**0**	**0**	**0**
Barn owl	1 (5.9%)	0	0	0	0	0	0	1 (100%)
**Tytonidae subtotal**	**1 (5.9%)**	**0**	**0**	**0**	**0**	**0**	**0**	**1 (100%)**
Carrion crow	59 (28.5%)	23 (39.0%)	0	0	1 (1.7%)	21 (35.6%)	3 (5.1%)	11 (18.6%)
Eurasian jay	0	0	0	0	0	0	0	0
Eurasian magpie	0	0	0	0	0	0	0	0
Hooded crow	0	0	0	0	0	0	0	0
Rook	1 (33.3%)	0	0	0	0	1 (100%)	0	0
Western jackdaw	0	0	0	0	0	0	0	0
**Corvidae subtotal**	**60 (23.7%)**	**23 (38.3%)**	**0**	**0**	**1 (1.7%)**	**22 (36.7%)**	**3 (5.0%)**	**11 (18.3%)**

**Table 2:** *Chlamydiaceae* infection rates of raptors and corvids per canton.

Greater Region	Swiss Canton	Number of Birds	*Chlamydiaceae* Positive (%)
Lake Geneva	Geneva	15	0
	Valais	11	0
Espace Mittelland	Bern	56	5 (8.9%)
	Fribourg	18	1 (5.6%)
	Solothurn	13	0
Northwestern Switzerland	Aargau	31	1 (3.2%)
	Basel District	4	0
Zurich	Zurich	132	32 (24.2%)
Eastern Switzerland	Glarus	1	0
	Grisons	10	0
	Schaffhausen	9	1 (11.1%)
	St. Gallen	6	0
	Thurgau	14	1 (7.1%)
Central Switzerland	Lucerne	115	2 (1.7%)
	Nidwalden	3	0
	Obwalden	5	1 (20.0%)
	Uri	1	0
	Zug	51	27 (52.9%)
Ticino	Ticino	6	0
Unknown	Unknown	93	9 (9.7%)

**Table 3:** Number of swabs per sampling site from raptors and corvids positive for *Chlamydiaceae*.

	Choanal Swabs Positive/Total (%)	Cloacal Swabs Positive/Total (%)	Fecal Swabs Positive/Total (%)
Raptors	15/299 (5.0%)	16/304 (5.3%)	0/42 (0%)
Corvids	57/220 (25.9%)	30/216 (13.9%)	1/47 (2.1%)
Total	72/519 (13.9%)	46/520 (8.8%)	1/89 (1.1%)

**Table 4:** Sequence length, sequence quality, first hit by nucleotide identity when compared against the NCBI database and accession number of partial 16S rRNA sequences generated in this study from eight samples from six raptors and 66 samples from 49 corvids from Switzerland.

Sample Nr.	Species Name (English)	Sequence Length (bp)	Sequence Quality (%)	First Hit	Nucleotide Identity (%)	Accession Number
**Raptors**						
14C	Common buzzard	245	26.1	*C. psittaci* M56	99.59	MT423441
311K	Common kestrel	269	80.3	*C. psittaci* M56	100	MT423442
556C	Common buzzard	239	30.1	*C. psittaci* M56	100	MT423443
556K	Common buzzard	278	83.1	*C. psittaci* M56	100	MT423444
566C	Common buzzard	278	80.9	*C. abortus* 15-58d44	100	MT423446
669K	Common buzzard	271	83.0	*C. psittaci* M56	100	MT423448
671C	Common buzzard	260	32.7	*C. psittaci* M56	98.85	MT423449
671K	Common buzzard	269	77.3	*C. psittaci* M56	100	MT423450
**Corvids**						
565C	Carrion crow	253	82.2	*C. abortus* 15-58d44	100	MT423445
621C	Rook	274	75.2	*C. abortus* 15-58d44	100	MT423447
686C	Carrion crow	271	40.6	*C. abortus* 15-58d44	99.63	MT423451
688C	Carrion crow	252	80.2	*C. abortus* 15-58d44	100	MT423452
688K	Carrion crow	279	73.8	*C. abortus* 15-58d44	100	MT423453
689C	Carrion crow	271	79.3	*C. abortus* 15-58d44	100	MT423454
689K	Carrion crow	249	25.7	*C. abortus* 15-58d44	95.58	MT423455
690C	Carrion crow	253	80.6	*C. abortus* 15-58d44	100	MT423456
696C	Carrion crow	276	78.3	*C. abortus* 15-58d44	100	MT423457
702C	Carrion crow	275	80.7	*C. abortus* 15-58d44	100	MT423458
705C	Carrion crow	278	71.6	*C. abortus* 15-58d44	100	MT423459
706C	Carrion crow	256	80.1	*C. abortus* 15-58d44	100	MT423460
711C	Carrion crow	260	85.4	*C. abortus* 15-58d44	100	MT423461
716K	Carrion crow	272	84.2	*C. abortus* 15-58d44	100	MT423462
721C	Carrion crow	253	81.4	*C. abortus* 15-58d44	100	MT423463
725C	Carrion crow	267	81.3	*C. abortus* 15-58d44	100	MT423464
735C	Carrion crow	267	76.8	*C. abortus* 15-58d44	100	MT423465
736C	Carrion crow	253	80.6	*C. abortus* 15-58d44	100	MT423466
736K	Carrion crow	271	76.8	*C. abortus* 15-58d44	100	MT423467
737C	Carrion crow	275	77.5	*C. abortus* 15-58d44	100	MT423468
737K	Carrion crow	315	63.8	*C. abortus* 15-58d44	96.96	MT423469
740C	Carrion crow	260	82.7	*C. abortus* 15-58d44	100	MT423470
740K	Carrion crow	260	73.5	*C. abortus* 15-58d44	100	MT423471
744C	Carrion crow	278	77.3	*C. abortus* 15-58d44	100	MT423472
746C	Carrion crow	266	84.6	*C. abortus* 15-58d44	100	MT423473
750C	Carrion crow	276	76.4	*C. abortus* 15-58d44	100	MT423474
750K	Carrion crow	250	21.2	*C. abortus* 15-58d44	98.40	MT423475
751C	Carrion crow	269	85.9	*C. abortus* 15-58d44	100	MT423476
751K	Carrion crow	226	32.7	*C. abortus* 15-58d44	100	MT423477
752C	Carrion crow	278	62.2	*C. abortus* 15-58d44	100	MT423478
752K	Carrion crow	253	51.0	*C. abortus* 15-58d44	100	MT423479
753C	Carrion crow	274	75.9	*C. abortus* 15-58d44	100	MT423480
754C	Carrion crow	267	82.8	*C. abortus* 15-58d44	100	MT423481
756C	Carrion crow	278	75.5	*C. abortus* 15-58d44	100	MT423482
756K	Carrion crow	240	28.3	*C. abortus* 15-58d44	99.17	MT423483
759C	Carrion crow	278	72.7	*C. abortus* 15-58d44	100	MT423484
760C	Carrion crow	279	68.1	*C. abortus* 15-58d44	100	MT423485
760K	Carrion crow	250	53.2	*C. abortus* 15-58d44	100	MT423486
761C	Carrion crow	256	81.3	*C. abortus* 15-58d44	100	MT423487
764C	Carrion crow	276	71.7	*C. abortus* 15-58d44	100	MT423488
765C	Carrion crow	278	78.8	*C. abortus* 15-58d44	100	MT423489
769C	Carrion crow	276	81.2	*C. abortus* 15-58d44	100	MT423490
770C	Carrion crow	256	75.4	*C. abortus* 15-58d44	100	MT423491
772C	Carrion crow	267	82.8	*C. abortus* 15-58d44	100	MT423492
772K	Carrion crow	270	80.4	*C. abortus* 15-58d44	100	MT423493
773C	Carrion crow	269	76.6	*C. abortus* 15-58d44	100	MT423494
774C	Carrion crow	277	75.1	*C. abortus* 15-58d44	100	MT423495
797C	Carrion crow	275	77.1	*C. abortus* 15-58d44	100	MT423496
797K	Carrion crow	265	64.2	*C. abortus* 15-58d44	100	MT423497
798C	Carrion crow	277	75.8	*C. abortus* 15-58d44	100	MT423498
798K	Carrion crow	271	81.2	*C. abortus* 15-58d44	100	MT423499
814C	Carrion crow	266	82.7	*C. abortus* 15-58d44	100	MT423500
814K	Carrion crow	278	74.8	*C. abortus* 15-58d44	100	MT423501
826C	Carrion crow	266	77.8	*C. abortus* 15-58d44	100	MT423502
846C	Carrion crow	276	71.7	*C. abortus* 15-58d44	100	MT423503
847C	Carrion crow	238	43.3	*C. abortus* 15-58d44	99.58	MT423504
848C	Carrion crow	267	82.8	*C. abortus* 15-58d44	100	MT423505
850C	Carrion crow	256	75.4	*C. abortus* 15-58d44	100	MT423506
850K	Carrion crow	278	65.5	*C. abortus* 15-58d44	100	MT423507
851C	Carrion crow	264	82.6	*C. abortus* 15-58d44	100	MT423508
856C	Carrion crow	267	80.1	*C. abortus* 15-58d44	100	MT423509
858C	Carrion crow	255	74.9	*C. abortus* 15-58d44	100	MT423510
858K	Carrion crow	271	77.1	*C. abortus* 15-58d44	100	MT423511
861C	Carrion crow	276	80.1	*C. abortus* 15-58d44	100	MT423512
861K	Carrion crow	270	40.0	*C. abortus* 15-58d44	99.63	MT423513
972C	Carrion crow	260	83.8	*C. abortus* 15-58d44	100	MT423514

**Table 5:** Sequence length, sequence quality, first hit by nucleotide identity when compared against the NCBI database and accession number of 16S rRNA (1481 bp) sequences generated in this study from four raptors and six corvids from Switzerland.

Sample Nr.	Species Name (English)	Sequence Length (bp)	Sequence Quality (%)	First Hit	Nucleotide Identity (%)	Accession Number
**Raptors**						
268C	Eurasian sparrowhawk	1000	86.1	*C. psittaci* Ful127	97.99	MT430892
311K	Common kestrel	921	78.8	*C. psittaci* M56	99.57	MT429304
556K	Common buzzard	1395	95.8	*C. psittaci* M56	100	MT430893
566C	Common buzzard	1147	94.9	C. psittaci nier_A113	100	MT430895
**Corvids**						
565C	Carrion crow	996	98.4	*C. psittaci* nier_A113	99.90	MT430894
621C	Rook	1357	91.4	*C. psittaci* nier_A113	100	MT430896
746C	Carrion crow	1218	95.7	*C. psittaci* nier_A113	99.92	MT430897
769C	Carrion crow	1370	95.5	*C. psittaci* nier_A113	99.85	MT430898
814C	Carrion crow	1379	93.8	*C. psittaci* nier_A97	99.93	MT430899
972C	Carrion crow	1071	93.8	*C. psittaci* nier_A113	99.72	MT430900

**Table 6:** Identified outer membrane protein A (*ompA*) genotype of *Chlamydiaceae* detected in nine raptors and 26 crows from various Swiss cantons.

Sample Nr.	Species Name (English)	Canton of Origin	Year of Sampling	Mean Cq Value *Chlamydiaceae* qPCR	*OmpA* Genotype	Accession Number
**Raptors**						
268C	Eurasian sparrowhawk	Unknown	2018	26.3	A	MT450242
311K	Common kestrel	Unknown	2018	26.8	M56	MT450243
511C	Common buzzard	Unknown	2019	38.3	M56	MT450244
556K	Common buzzard	Zurich	2019	14.1	M56	MT450245
566C	Common buzzard	Obwalden	2019	29.8	1V	MT450247
669C	Common buzzard	Unknown	2019	27.9	M56	MT450249
671K	Common buzzard	Thurgau	2019	27.1	M56	MT450250
683C	Common buzzard	Unknown	2019	33.3	A	MT450251
877K	Long-eared owl	Bern	2019	31.3	M56	MT450275
**Corvids**						
565C	Carrion crow	Aargau	2019	27.9	1V	MT450246
621C	Rook	Unknown	2019	23.9	1V	MT450248
688C	Carrion crow	Zurich	2019	28.2	1V	MT450252
689C	Carrion crow	Zurich	2019	26.8	1V	MT450253
690C	Carrion crow	Zurich	2019	29.4	1V	MT450254
706C	Carrion crow	Zurich	2019	30.2	D	MT450255
711C	Carrion crow	Bern	2019	31.6	1V	MT450256
716K	Carrion crow	Bern	2019	30.6	D	MT450257
721C	Carrion crow	Zurich	2019	29.9	1V	MT450258
735C	Carrion crow	Zug	2019	28.4	1V	MT450259
736C	Carrion crow	Zug	2019	28.7	6N	MT450260
740C	Carrion crow	Zug	2019	27.7	1V	MT450261
746C	Carrion crow	Zug	2019	24.9	1V	MT450262
751C	Carrion crow	Zug	2019	20.2	1V	MT450263
752C	Carrion crow	Zug	2019	30.4	1V	MT450264
761C	Carrion crow	Zug	2019	29.7	1V	MT450265
772K	Carrion crow	Zug	2019	26.8	1V	MT450266
798K	Carrion crow	Zurich	2019	26.7	1V	MT450267
814C	Carrion crow	Zurich	2019	25.6	1V	MT450268
826C	Carrion crow	Zurich	2019	29.3	1V	MT450269
848C	Carrion crow	Zurich	2019	26.6	D	MT450270
850K	Carrion crow	Zurich	2019	27.9	1V	MT450271
856C	Carrion crow	Zurich	2019	29.3	1V	MT450272
858C	Carrion crow	Zurich	2019	27.8	1V	MT450273
861K	Carrion crow	Unknown	2019	23.6	1V	MT450274
972C	Carrion crow	Fribourg	2019	27.4	1V	MT450276

**Table 7:** Number of raptors and corvids with sample types obtained in this study per bird species.

Order	Family	Species Name (Latin)	Species Name (English)	Number of Birds	Number of Choanal Swabs	Number of Cloacal Swabs	Number of Fecal Swabs
**Accipitriformes**	Accipitridae	*Gypaetus barbatus*	Bearded vulture	1	1	1	0
		*Milvus migrans*	Black kite	6	4	4	3
		*Buteo buteo*	Common buzzard	142	127	128	14
		*Accipiter nisus*	Eurasian sparrowhawk	32	32	32	1
		*Pernis apivorus*	European honey-buzzard	1	1	1	0
		*Aquila chrysaetos*	Golden eagle	6	3	6	0
		*Circus pygargus*	Montagu’s harrier	1	1	1	0
		*Accipiter gentilis*	Northern goshawk	2	2	2	0
		*Milvus milvus*	Red kite	23	20	21	2
**Falconiformes**	Falconidae	*Falco tinnunculus*	Common kestrel	66	54	55	12
		*Falco subbuteo*	Eurasian hobby	4	3	3	1
		*Falco peregrinus*	Peregrine falcon	1	1	1	0
**Passeriformes**	Corvidae	*Corvus corone*	Carrion crow	207	190	187	19
		*Garrulus glandarius*	Eurasian jay	9	9	8	1
		*Pica pica*	Eurasian magpie	30	16	16	22
		*Corvus cornix*	Hooded crow	1	1	1	0
		*Corvus frugilegus*	Rook	3	3	3	2
		*Corvus monedula*	Western jackdaw	3	1	1	3
**Strigiformes**	Strigidae	*Bubo bubo*	Eurasian eagle-owl	4	4	4	0
		*Asio otus*	Long-eared owl	17	15	14	2
		*Strix aluco*	Tawny owl	18	14	14	6
	Tytonidae	*Tyto alba*	Barn owl	17	17	17	1
**Total**				**594**	**519**	**520**	**89**

**Table 8:** Detailed information about the primers and probes used in this study for the detection of *Chlamydiaceae* in raptors and corvids, including their final concentration in the PCR reagent mix. eGFP = enhanced green fluorescent protein (used as internal amplification control), *ompA* = outer membrane protein A; qPCR = real-time PCR.

Method	Target	Final Concentration	Primer & Probe	Sequence (5′–3′)	Amplicon Size (Base Pairs)	Annealing Temperature (°C)	References
*Chlamydiaceae* 23S rRNA qPCR	23S rRNA	500 nM	Ch23S-F Ch23S-R	CTGAAACCAGTAGCTTATAAGCGGT ACCTCGCCGTTTAACTTAACTCC	111	60	Ehricht et al. (2006) [64]
		200 nM	Ch23S-P	FAM-CTCATCATGCAAAAGGCACGCCG-TAMRA			
Internal amplification control	eGFP	200 nM	eGFP-1-F	GACCACTACCAGCAGAACAC	177		Hoffmann et al. (2006) [65] PCR modified by Blumer et al. (2011) [66]
			eGFP-10-R	CTTGTACAGCTCGTCCATGC			
			eGFP-HEX	HEX-AGCACCCAGTCCGCCCTGAGCA-BHQ1			
*C. psittaci ompA* qPCR	*ompA*	900 nM	CppsOMP1-F	CACTATGTGGGAAGGTGCTTCA	76	60	Pantchev et al. (2009) [67]
			CppsOMP1-R	CTGCGCGGATGCTAATGG			
		200 nM	CppsOMP1-S	FAM-CGCTACTTGGTGTGAC-TAMRA			
Internal amplification control	eGFP	900 nM	eGFP-1-F	GACCACTACCAGCAGAACAC	132		Hoffmann et al. (2005) [68]
			eGFP-2-R	GAACTCCAGCAGGACCATG			
		200 nM	eGFP-HEX	HEX-AGCACCCAGTCCGCCCTGAGCA-BHQ1			
16S rRNA PCR (partial)	16S rRNA	300 nM	16S IGF (short) 16S IGR (short)	GATGAGGCATGCAAGTCGAACG CCAGTGTTGGCGGTCAATCTCTC	278	65	Blumer et al. (2007) [70], Modified from Everett et al. (1999) [75]
16S rRNA PCR (near-full length)	16S rRNA	300 nM	16S-IGF 16S-B1	CGGCGTGGATGAGGCAT TACGGYTACCTTGTTACGACTT	1481	57.5	Everett et al. (1999) [69] Hosokawa et al. (2006) [72]
C. *buteonis oxaA* qPCR	*oxaA*	600 nM	RSHA-F	ATTTCCAACACGCACTGCAT	80	60	Laroucau et al. (2019) [3]
			RSHA-R	TGGGACTAGGTGTTCTCCCT			
		200 nM	RSHA-P	FAM-GGACAACATGCCTAGATGAAGA-TAMRA			
Internal amplification control	eGFP	400 nM	eGFP-1-F	GACCACTACCAGCAGAACAC	132		Hoffmann et al. (2005) [68]
			eGFP-2-R	GAACTCCAGCAGGACCATG			
		200 nM	eGFP-HEX	HEX-AGCACCCAGTCCGCCCTGAGCA-BHQ1			
*ompA* typing PCR	*ompA*	200 nM	ompA F (CTU)	ATGAAAAAACTCTTGAAATCGG	1212	49	Sachse et al. (2008) [73]
			ompA rev	TCCTTAGAATCTGAATTGAGC			

## Supplementary Materials

The following are available online at https://www.mdpi.com/2076-0817/9/9/724/s1, Table S1: Sequence length, sequence quality, first hit by nucleotide identity when compared against the NCBI database and accession number of outer membrane protein A (ompA) sequences generated in this study from nine raptors and 26 corvids from Switzerland, Table S2: Details on origin and analysis results of all swab samples collected and processed in the frame of the present study.
