# Supplementary material for: Occurrence of Chlamydiaceae in Raptors and Crows in Switzerland
Source: Pathogens. 2020 Sep 2;9(9):724. doi: 10.3390/pathogens9090724 (PMC7558692; doi:10.3390/pathogens9090724)
Supplement: Supplementary file 1 [file pathogens-09-00724-s001.pdf]

Supplementary Material

Supplementary Table S1. Sequence length, sequence quality, first hit by nucleotide identity when compared against the NCBI database and accession number of outer membrane protein A (ompA) sequences generated in this study from nine raptors and 26 corvids from Switzerland.

Sample Nr.	Species Name (English)	Sequence Length (bp)	Sequence Quality (%)	First Hit	Nucleotide Identity (%)	Accession Number
268C	Eurasian sparrowhawk	1080	97.0	*C. psittaci* Ful127	99.91	MT450242
311K	Common kestrel	687	93.1	*C. psittaci* M56	99.56	MT450243
511C	Common buzzard	978	67.2	*C. psittaci* M56	100	MT450244
556K	Common buzzard	1116	97.2	*C. psittaci* M56	100	MT450245
565C	Carrion crow	1041	97.5	*C. abortus* 15-58d44	99.43	MT450246
566C	Common buzzard	1080	96.3	*C. abortus* 15-58d44	99.35	MT450247
621C	Rook	1083	97.7	*C. abortus* 15-58d44	99.26	MT450248
669C	Common buzzard	1011	98.1	*C. psittaci* M56	100	MT450249
671K	Common buzzard	933	86.4	*C. psittaci* M56	100	MT450250
683C	Common buzzard	975	71.0	*C. psittaci* Ful127	99.49	MT450251
688C	Carrion crow	1005	97.9	*C. abortus* 15-58d44	99.6	MT450252
689C	Carrion crow	1080	97.8	*C. abortus* 15-58d44	99.26	MT450253
690C	Carrion crow	972	96.8	*C. abortus* 15-58d44	99.9	MT450254
706C	Carrion crow	912	98.3	*C. psittaci* NJ1	96.83	MT450255
711C	Carrion crow	960	95.1	*C. abortus* 15-58d44	99.58	MT450256
716K	Carrion crow	1026	98.1	*C. psittaci* NJ1	97.08	MT450257
721C	Carrion crow	957	99.6	*C. abortus* 15-58d44	99.17	MT450258
735C	Carrion crow	975	99.3	*C. abortus* 15-58d44	99.39	MT450259
736C	Carrion crow	642	97.8	*C. psittaci* nier_A113.6N	100	MT450260
740C	Carrion crow	1008	98.1	*C. abortus* 15-58d44	99.9	MT450261
746C	Carrion crow	1026	98.9	*C. abortus* 15-58d44	99.61	MT450262
751C	Carrion crow	1038	98.8	*C. abortus* 15-58d44	99.23	MT450263
752C	Carrion crow	357	82.2	*C. abortus* 15-58d44	100	MT450264
761C	Carrion crow	954	100	*C. abortus* 15-58d44	99.58	MT450265
772K	Carrion crow	1026	98.5	*C. abortus* 15-58d44	99.61	MT450266
798K	Carrion crow	1038	97.1	*C. abortus* 15-58d44	99.33	MT450267
814C	Carrion crow	1050	98.8	*C. abortus* 15-58d44	99.62	MT450268
826C	Carrion crow	1083	89.4	*C. abortus* 15-58d44	99.54	MT450269
848C	Carrion crow	1023	96.4	*C. psittaci* NJ1	97.07	MT450270
850K	Carrion crow	966	100	*C. abortus* 15-58d44	99.59	MT450271

856C	Carrion crow	942	98.4	*C. abortus* 15-58d44	99.43	MT450272
858C	Carrion crow	966	98.5	*C. abortus* 15-58d44	99.59	MT450273
861K	Carrion crow	1065	98.1	*C. abortus* 15-58d44	99.53	MT450274
877K	Long-eared owl	390	97.2	*C. psittaci* M56	97.17	MT450275
972C	Carrion crow	996	97.8	*C. abortus* 15-58d44	99.6	MT450276

Supplementary Table S2. Details on origin and analysis results of all swab samples collected and processed in the frame of the present study. (neg. = negative, pos. = positive, n.d. = not determined, N/A = not applicable)

Sample	Species Name (English)	Species Name (Latin)	Place of Origin	Canton	Material	*Chlamydiaceae* qPCR (Ø CT value)	*C. psittaci* qPCR (Ø CT value)	*C. buteonis* qPCR	Final Classification
**8C**	Tawny owl	*Strix aluco*	Unknown	LU	Choana	neg.	n.d.	n.d.	N/A
**8K**	Tawny owl	*Strix aluco*	Unknown	LU	Cloaca	neg.	n.d.	n.d.	N/A
**8Kot**	Tawny owl	*Strix aluco*	Unknown	LU	Feces	neg.	n.d.	n.d.	N/A
**11C**	Eurasian sparrowhawk	*Accipiter nisus*	6028 Herlisberg	LU	Choana	neg.	n.d.	n.d.	N/A
**11K**	Eurasian sparrowhawk	*Accipiter nisus*	6028 Herlisberg	LU	Cloaca	neg.	n.d.	n.d.	N/A
**12C**	Common buzzard	*Buteo buteo*	6210 Sursee	LU	Choana	neg.	n.d.	n.d.	N/A
**12K**	Common buzzard	*Buteo buteo*	6210 Sursee	LU	Cloaca	neg.	n.d.	n.d.	N/A
**13C**	Common buzzard	*Buteo buteo*	Unknown	N/A	Choana	neg.	n.d.	n.d.	N/A
**13K**	Common buzzard	*Buteo buteo*	Unknown	N/A	Cloaca	neg.	n.d.	n.d.	N/A
**14C**	Common buzzard	*Buteo buteo*	6000 Luzern	LU	Choana	pos. (32.7)	neg.	neg.	*C. psittaci* M56
**14K**	Common buzzard	*Buteo buteo*	6000 Luzern	LU	Cloaca	pos. (29.4)	neg.	neg.	Not identified
**15C**	Barn owl	*Tyto alba*	3114 Wichtrach	BE	Choana	neg.	n.d.	n.d.	N/A
**15K**	Barn owl	*Tyto alba*	3114 Wichtrach	BE	Cloaca	pos. (36.3)	neg.	neg.	Not identified
**16C**	Tawny owl	*Strix aluco*	6130 Willisau	LU	Choana	neg.	n.d.	n.d.	N/A
**16K**	Tawny owl	*Strix aluco*	6130 Willisau	LU	Cloaca	neg.	n.d.	n.d.	N/A
**17C**	Red kite	*Milvus milvus*	6289 Hämikon	LU	Choana	neg.	n.d.	n.d.	N/A
**17K**	Red kite	*Milvus milvus*	6289 Hämikon	LU	Cloaca	neg.	n.d.	n.d.	N/A
**18C**	Common kestrel	*Falco tinnunculus*	6246 Altishofen	LU	Choana	neg.	n.d.	n.d.	N/A
**18K**	Common kestrel	*Falco tinnunculus*	6246 Altishofen	LU	Cloaca	neg.	n.d.	n.d.	N/A
**19C**	Common buzzard	*Buteo buteo*	6244 Nebikon	LU	Choana	neg.	n.d.	n.d.	N/A
**19K**	Common buzzard	*Buteo buteo*	6244 Nebikon	LU	Cloaca	neg.	n.d.	n.d.	N/A
**35Kot**	Carrion crow	*Corvus corone*	Unknown	N/A	Feces	neg.	n.d.	n.d.	N/A
**55Kot**	Eurasian magpie	*Pica pica*	5727 Oberkulm	AG	Feces	neg.	n.d.	n.d.	N/A
**58C**	Common buzzard	*Buteo buteo*	6032 Emmen	LU	Choana	neg.	n.d.	n.d.	N/A
**58K**	Common buzzard	*Buteo buteo*	6032 Emmen	LU	Cloaca	neg.	n.d.	n.d.	N/A
**60Kot**	Black kite	*Milvus migrans*	6032 Emmen	LU	Feces	neg.	n.d.	n.d.	N/A
**68C**	Red kite	*Milvus milvus*	6017 Ruswil	LU	Choana	neg.	n.d.	n.d.	N/A
**68K**	Red kite	*Milvus milvus*	6017 Ruswil	LU	Cloaca	neg.	n.d.	n.d.	N/A
**102Kot**	Eurasian magpie	*Pica pica*	5623 Boswil	AG	Feces	neg.	n.d.	n.d.	N/A

**103Kot**	Eurasian magpie	*Pica pica*	5623 Boswil	AG	Feces	neg.	n.d.	n.d.	N/A
**105Kot**	Carrion crow	*Corvus corone*	6203 Sempach-Station	LU	Feces	neg.	n.d.	n.d.	N/A
**106Kot**	Carrion crow	*Corvus corone*	6033 Buchrain	LU	Feces	neg.	n.d.	n.d.	N/A
**126C**	Carrion crow	*Corvus corone*	6210 Sursee	LU	Choana	neg.	n.d.	n.d.	N/A
**126K**	Carrion crow	*Corvus corone*	6210 Sursee	LU	Cloaca	neg.	n.d.	n.d.	N/A
**132C**	Long-eared owl	*Asio otus*	6213 Knutwil	LU	Choana	neg.	n.d.	n.d.	N/A
**132K**	Long-eared owl	*Asio otus*	6213 Knutwil	LU	Cloaca	neg.	n.d.	n.d.	N/A
**160C**	Eurasian jay	*Garrulus glandarius*	6204 Sempach	LU	Choana	neg.	n.d.	n.d.	N/A
**160K**	Eurasian jay	*Garrulus glandarius*	6204 Sempach	LU	Cloaca	neg.	n.d.	n.d.	N/A
**160Kot**	Eurasian jay	*Garrulus glandarius*	6204 Sempach	LU	Feces	neg.	n.d.	n.d.	N/A
**163C**	Eurasian magpie	*Pica pica*	6032 Emmen	LU	Choana	neg.	n.d.	n.d.	N/A
**163K**	Eurasian magpie	*Pica pica*	6032 Emmen	LU	Cloaca	neg.	n.d.	n.d.	N/A
**163Kot**	Eurasian magpie	*Pica pica*	6032 Emmen	LU	Feces	neg.	n.d.	n.d.	N/A
**166Kot**	Carrion crow	*Corvus corone*	6252 Dagmersellen	LU	Feces	neg.	n.d.	n.d.	N/A
**167Kot**	Carrion crow	*Corvus corone*	6216 Mauensee	LU	Feces	neg.	n.d.	n.d.	N/A
**176Kot**	Eurasian magpie	*Pica pica*	8157 Oberglatt	ZH	Feces	neg.	n.d.	n.d.	N/A
**178C**	Carrion crow	*Corvus corone*	6005 Luzern	LU	Choana	neg.	n.d.	n.d.	N/A
**178K**	Carrion crow	*Corvus corone*	6005 Luzern	LU	Cloaca	neg.	n.d.	n.d.	N/A
**178Kot**	Carrion crow	*Corvus corone*	6005 Luzern	LU	Feces	neg.	n.d.	n.d.	N/A
**179Kot**	Eurasian magpie	*Pica pica*	6003 Luzern	LU	Feces	neg.	n.d.	n.d.	N/A
**199C**	Carrion crow	*Corvus corone*	6000 Luzern	LU	Choana	neg.	n.d.	n.d.	N/A
**199K**	Carrion crow	*Corvus corone*	6000 Luzern	LU	Cloaca	neg.	n.d.	n.d.	N/A
**209Kot**	Carrion crow	*Corvus corone*	6206 Neuenkirch	LU	Feces	neg.	n.d.	n.d.	N/A
**213C**	Carrion crow	*Corvus corone*	6010 Kriens	LU	Choana	neg.	n.d.	n.d.	N/A
**213K**	Carrion crow	*Corvus corone*	6010 Kriens	LU	Cloaca	neg.	n.d.	n.d.	N/A
**214C**	Carrion crow	*Corvus corone*	6010 Kriens	LU	Choana	neg.	n.d.	n.d.	N/A
**214K**	Carrion crow	*Corvus corone*	6010 Kriens	LU	Cloaca	neg.	n.d.	n.d.	N/A
**215C**	Carrion crow	*Corvus corone*	6010 Kriens	LU	Choana	neg.	n.d.	n.d.	N/A
**215K**	Carrion crow	*Corvus corone*	6010 Kriens	LU	Cloaca	neg.	n.d.	n.d.	N/A
**218Kot**	Common kestrel	*Falco tinnunculus*	6294 Ermensee	LU	Feces	neg.	n.d.	n.d.	N/A
**223Kot**	Common kestrel	*Falco tinnunculus*	6289 Hämikon	LU	Feces	neg.	n.d.	n.d.	N/A
**228C**	Long-eared owl	*Asio otus*	6280 Hochdorf	LU	Choana	neg.	n.d.	n.d.	N/A
**228K**	Long-eared owl	*Asio otus*	6280 Hochdorf	LU	Cloaca	neg.	n.d.	n.d.	N/A
**231Kot**	Long-eared owl	*Asio otus*	6280 Hochdorf	LU	Feces	neg.	n.d.	n.d.	N/A
**237Kot**	Black kite	*Milvus migrans*	6017 Ruswil	LU	Feces	neg.	n.d.	n.d.	N/A
**241C**	Eurasian magpie	*Pica pica*	6036 Dierikon	LU	Choana	neg.	n.d.	n.d.	N/A
**241K**	Eurasian magpie	*Pica pica*	6036 Dierikon	LU	Cloaca	neg.	n.d.	n.d.	N/A
**241Kot**	Eurasian magpie	*Pica pica*	6036 Dierikon	LU	Feces	neg.	n.d.	n.d.	N/A
**246Kot**	Common kestrel	*Falco tinnunculus*	6032 Emmen	LU	Feces	neg.	n.d.	n.d.	N/A
**248C**	Common kestrel	*Falco tinnunculus*	3032 Hinterkappelen	BE	Choana	neg.	n.d.	n.d.	N/A
**248K**	Common kestrel	*Falco tinnunculus*	3032 Hinterkappelen	BE	Cloaca	neg.	n.d.	n.d.	N/A
**255C**	Common buzzard	*Buteo buteo*	Unknown	N/A	Choana	neg.	n.d.	n.d.	N/A
**255K**	Common buzzard	*Buteo buteo*	Unknown	N/A	Cloaca	neg.	n.d.	n.d.	N/A

**256C**	Common buzzard	*Buteo buteo*	Unknown	N/A	Choana	neg.	n.d.	n.d.	N/A
**256K**	Common buzzard	*Buteo buteo*	Unknown	N/A	Cloaca	neg.	n.d.	n.d.	N/A
**257C**	Common buzzard	*Buteo buteo*	Unknown	N/A	Choana	neg.	n.d.	n.d.	N/A
**257K**	Common buzzard	*Buteo buteo*	Unknown	N/A	Cloaca	neg.	n.d.	n.d.	N/A
**258C**	Common buzzard	*Buteo buteo*	Unknown	N/A	Choana	neg.	n.d.	n.d.	N/A
**258K**	Common buzzard	*Buteo buteo*	Unknown	N/A	Cloaca	neg.	n.d.	n.d.	N/A
**259C**	Tawny owl	*Strix aluco*	Unknown	N/A	Choana	neg.	n.d.	n.d.	N/A
**259K**	Tawny owl	*Strix aluco*	Unknown	N/A	Cloaca	neg.	n.d.	n.d.	N/A
**260C**	Long-eared owl	*Asio otus*	Unknown	N/A	Choana	neg.	n.d.	n.d.	N/A
**260K**	Long-eared owl	*Asio otus*	Unknown	N/A	Cloaca	neg.	n.d.	n.d.	N/A
**261C**	Common kestrel	*Falco tinnunculus*	Unknown	N/A	Choana	neg.	n.d.	n.d.	N/A
**261K**	Common kestrel	*Falco tinnunculus*	Unknown	N/A	Cloaca	neg.	n.d.	n.d.	N/A
**262C**	Eurasian sparrowhawk	*Accipiter nisus*	Unknown	N/A	Choana	neg.	n.d.	n.d.	N/A
**262K**	Eurasian sparrowhawk	*Accipiter nisus*	Unknown	N/A	Cloaca	neg.	n.d.	n.d.	N/A
**263C**	Common buzzard	*Buteo buteo*	Unknown	N/A	Choana	neg.	n.d.	n.d.	N/A
**263K**	Common buzzard	*Buteo buteo*	Unknown	N/A	Cloaca	neg.	n.d.	n.d.	N/A
**264C**	Eurasian hobby	*Falco subbuteo*	Unknown	N/A	Choana	neg.	n.d.	n.d.	N/A
**264K**	Eurasian hobby	*Falco subbuteo*	Unknown	N/A	Cloaca	neg.	n.d.	n.d.	N/A
**265C**	Barn owl	*Tyto alba*	Unknown	N/A	Choana	neg.	n.d.	n.d.	N/A
**265K**	Barn owl	*Tyto alba*	Unknown	N/A	Cloaca	neg.	n.d.	n.d.	N/A
**266C**	Red kite	*Milvus milvus*	Unknown	N/A	Choana	neg.	n.d.	n.d.	N/A
**266K**	Red kite	*Milvus milvus*	Unknown	N/A	Cloaca	neg.	n.d.	n.d.	N/A
**267C**	Common kestrel	*Falco tinnunculus*	Unknown	N/A	Choana	neg.	n.d.	n.d.	N/A
**267K**	Common kestrel	*Falco tinnunculus*	Unknown	N/A	Cloaca	neg.	n.d.	n.d.	N/A
**268C**	Eurasian sparrowhawk	*Accipiter nisus*	Unknown	N/A	Choana	pos. (26.3)	pos. (24.9)	neg.	*C. psittaci* A
**268K**	Eurasian sparrowhawk	*Accipiter nisus*	Unknown	N/A	Cloaca	neg.	n.d.	n.d.	N/A
**269C**	Common buzzard	*Buteo buteo*	Unknown	N/A	Choana	neg.	n.d.	n.d.	N/A
**269K**	Common buzzard	*Buteo buteo*	Unknown	N/A	Cloaca	neg.	n.d.	n.d.	N/A
**270C**	Common buzzard	*Buteo buteo*	Unknown	N/A	Choana	neg.	n.d.	n.d.	N/A
**270K**	Common buzzard	*Buteo buteo*	Unknown	N/A	Cloaca	neg.	n.d.	n.d.	N/A
**271C**	Red kite	*Milvus milvus*	Unknown	N/A	Choana	neg.	n.d.	n.d.	N/A
**271K**	Red kite	*Milvus milvus*	Unknown	N/A	Cloaca	neg.	n.d.	n.d.	N/A
**272C**	Common buzzard	*Buteo buteo*	Unknown	N/A	Choana	neg.	n.d.	n.d.	N/A
**272K**	Common buzzard	*Buteo buteo*	Unknown	N/A	Cloaca	neg.	n.d.	n.d.	N/A
**273C**	Red kite	*Milvus milvus*	Unknown	N/A	Choana	neg.	n.d.	n.d.	N/A
**273K**	Red kite	*Milvus milvus*	Unknown	N/A	Cloaca	neg.	n.d.	n.d.	N/A
**274C**	Red kite	*Milvus milvus*	4566 Halten	SO	Choana	neg.	n.d.	n.d.	N/A
**274K**	Red kite	*Milvus milvus*	4566 Halten	SO	Cloaca	neg.	n.d.	n.d.	N/A
**275C**	Eurasian sparrowhawk	*Accipiter nisus*	2545 Seuzach	SO	Choana	neg.	n.d.	n.d.	N/A
**275K**	Eurasian sparrowhawk	*Accipiter nisus*	2545 Seuzach	SO	Cloaca	neg.	n.d.	n.d.	N/A
**276C**	Eurasian sparrowhawk	*Accipiter nisus*	3000 Bern	BE	Choana	neg.	n.d.	n.d.	N/A
**276K**	Eurasian sparrowhawk	*Accipiter nisus*	3000 Bern	BE	Cloaca	neg.	n.d.	n.d.	N/A
**277C**	Eurasian sparrowhawk	*Accipiter nisus*	3255 Rapperswil	BE	Choana	neg.	n.d.	n.d.	N/A

**277K**	Eurasian sparrowhawk	*Accipiter nisus*	3255 Rapperswil	BE	Cloaca	neg.	n.d.	n.d.	N/A
**278C**	Tawny owl	*Strix aluco*	4704 Wolfisberg	BE	Choana	neg.	n.d.	n.d.	N/A
**278K**	Tawny owl	*Strix aluco*	4704 Wolfisberg	BE	Cloaca	neg.	n.d.	n.d.	N/A
**279C**	Tawny owl	*Strix aluco*	Unknown	N/A	Choana	neg.	n.d.	n.d.	N/A
**279K**	Tawny owl	*Strix aluco*	Unknown	N/A	Cloaca	neg.	n.d.	n.d.	N/A
**280C**	Tawny owl	*Strix aluco*	Unknown	N/A	Choana	neg.	n.d.	n.d.	N/A
**280K**	Tawny owl	*Strix aluco*	Unknown	N/A	Cloaca	neg.	n.d.	n.d.	N/A
**281C**	Tawny owl	*Strix aluco*	8805 Richterswil	ZH	Choana	neg.	n.d.	n.d.	N/A
**281K**	Tawny owl	*Strix aluco*	8805 Richterswil	ZH	Cloaca	neg.	n.d.	n.d.	N/A
**282C**	Barn owl	*Tyto alba*	Unknown	N/A	Choana	neg.	n.d.	n.d.	N/A
**282K**	Barn owl	*Tyto alba*	Unknown	N/A	Cloaca	neg.	n.d.	n.d.	N/A
**283C**	Barn owl	*Tyto alba*	Unknown	N/A	Choana	neg.	n.d.	n.d.	N/A
**283K**	Barn owl	*Tyto alba*	Unknown	N/A	Cloaca	neg.	n.d.	n.d.	N/A
**284C**	Barn owl	*Tyto alba*	3257 Ammerzwil	BE	Choana	neg.	n.d.	n.d.	N/A
**284K**	Barn owl	*Tyto alba*	3257 Ammerzwil	BE	Cloaca	neg.	n.d.	n.d.	N/A
**285C**	Long-eared owl	*Asio otus*	3454 Sumiswald	BE	Choana	neg.	n.d.	n.d.	N/A
**285K**	Long-eared owl	*Asio otus*	3454 Sumiswald	BE	Cloaca	neg.	n.d.	n.d.	N/A
**286C**	Barn owl	*Tyto alba*	4460 Gelterkinden	BL	Choana	neg.	n.d.	n.d.	N/A
**286K**	Barn owl	*Tyto alba*	4460 Gelterkinden	BL	Cloaca	neg.	n.d.	n.d.	N/A
**287C**	Barn owl	*Tyto alba*	Unknown	N/A	Choana	neg.	n.d.	n.d.	N/A
**287K**	Barn owl	*Tyto alba*	Unknown	N/A	Cloaca	neg.	n.d.	n.d.	N/A
**288C**	Long-eared owl	*Asio otus*	Unknown	N/A	Choana	neg.	n.d.	n.d.	N/A
**288K**	Long-eared owl	*Asio otus*	Unknown	N/A	Cloaca	neg.	n.d.	n.d.	N/A
**289C**	Barn owl	*Tyto alba*	Unknown	N/A	Choana	neg.	n.d.	n.d.	N/A
**289K**	Barn owl	*Tyto alba*	Unknown	N/A	Cloaca	neg.	n.d.	n.d.	N/A
**293Kot**	Common kestrel	*Falco tinnunculus*	Unknown	N/A	Feces	neg.	n.d.	n.d.	N/A
**294Kot**	Common kestrel	*Falco tinnunculus*	Unknown	N/A	Feces	neg.	n.d.	n.d.	N/A
**295Kot**	Common kestrel	*Falco tinnunculus*	Unknown	N/A	Feces	neg.	n.d.	n.d.	N/A
**296Kot**	Common kestrel	*Falco tinnunculus*	Unknown	N/A	Feces	neg.	n.d.	n.d.	N/A
**297Kot**	Common buzzard	*Buteo buteo*	Unknown	N/A	Feces	neg.	n.d.	n.d.	N/A
**298C**	Common kestrel	*Falco tinnunculus*	3294 Büren a.A.	BE	Choana	neg.	n.d.	n.d.	N/A
**298K**	Common kestrel	*Falco tinnunculus*	3294 Büren a.A.	BE	Cloaca	neg.	n.d.	n.d.	N/A
**299C**	Common kestrel	*Falco tinnunculus*	4710 Balsthal	SO	Choana	neg.	n.d.	n.d.	N/A
**299K**	Common kestrel	*Falco tinnunculus*	4710 Balsthal	SO	Cloaca	neg.	n.d.	n.d.	N/A
**300C**	Common kestrel	*Falco tinnunculus*	3315 Bätterkinden	BE	Choana	neg.	n.d.	n.d.	N/A
**300K**	Common kestrel	*Falco tinnunculus*	3315 Bätterkinden	BE	Cloaca	neg.	n.d.	n.d.	N/A
**301C**	Common kestrel	*Falco tinnunculus*	Unknown	N/A	Choana	neg.	n.d.	n.d.	N/A
**301K**	Common kestrel	*Falco tinnunculus*	Unknown	N/A	Cloaca	neg.	n.d.	n.d.	N/A
**302C**	Common kestrel	*Falco tinnunculus*	4566 Oekingen	SO	Choana	neg.	n.d.	n.d.	N/A
**302K**	Common kestrel	*Falco tinnunculus*	4566 Oekingen	SO	Cloaca	neg.	n.d.	n.d.	N/A
**303C**	Common kestrel	*Falco tinnunculus*	3303 Jegenstorf	BE	Choana	neg.	n.d.	n.d.	N/A
**303K**	Common kestrel	*Falco tinnunculus*	3303 Jegenstorf	BE	Cloaca	neg.	n.d.	n.d.	N/A
**304C**	Common kestrel	*Falco tinnunculus*	3427 Utzenstorf	BE	Choana	neg.	n.d.	n.d.	N/A

**304K**	Common kestrel	*Falco tinnunculus*	3427 Utzenstorf	BE	Cloaca	neg.	n.d.	n.d.	N/A
**305C**	Common kestrel	*Falco tinnunculus*	Unknown	N/A	Choana	neg.	n.d.	n.d.	N/A
**305K**	Common kestrel	*Falco tinnunculus*	Unknown	N/A	Cloaca	neg.	n.d.	n.d.	N/A
**306C**	Common kestrel	*Falco tinnunculus*	Unknown	N/A	Choana	neg.	n.d.	n.d.	N/A
**306K**	Common kestrel	*Falco tinnunculus*	Unknown	N/A	Cloaca	neg.	n.d.	n.d.	N/A
**307C**	Common kestrel	*Falco tinnunculus*	3305 Scheunen	BE	Choana	neg.	n.d.	n.d.	N/A
**307K**	Common kestrel	*Falco tinnunculus*	3305 Scheunen	BE	Cloaca	neg.	n.d.	n.d.	N/A
**308C**	Common kestrel	*Falco tinnunculus*	3314 Schalunen	BE	Choana	neg.	n.d.	n.d.	N/A
**308K**	Common kestrel	*Falco tinnunculus*	3314 Schalunen	BE	Cloaca	neg.	n.d.	n.d.	N/A
**309C**	Common kestrel	*Falco tinnunculus*	Unknown	N/A	Choana	neg.	n.d.	n.d.	N/A
**309K**	Common kestrel	*Falco tinnunculus*	Unknown	N/A	Cloaca	neg.	n.d.	n.d.	N/A
**310C**	Common kestrel	*Falco tinnunculus*	Unknown	N/A	Choana	neg.	n.d.	n.d.	N/A
**310K**	Common kestrel	*Falco tinnunculus*	Unknown	N/A	Cloaca	neg.	n.d.	n.d.	N/A
**311C**	Common kestrel	*Falco tinnunculus*	Unknown	N/A	Choana	neg.	n.d.	n.d.	N/A
**311K**	Common kestrel	*Falco tinnunculus*	Unknown	N/A	Cloaca	pos. (26.8)	neg.	neg.	*C. psittaci* M56
**312C**	Common kestrel	*Falco tinnunculus*	Unknown	N/A	Choana	neg.	n.d.	n.d.	N/A
**312K**	Common kestrel	*Falco tinnunculus*	Unknown	N/A	Cloaca	neg.	n.d.	n.d.	N/A
**313C**	Common kestrel	*Falco tinnunculus*	Unknown	N/A	Choana	neg.	n.d.	n.d.	N/A
**313K**	Common kestrel	*Falco tinnunculus*	Unknown	N/A	Cloaca	neg.	n.d.	n.d.	N/A
**314C**	Common kestrel	*Falco tinnunculus*	Unknown	N/A	Choana	neg.	n.d.	n.d.	N/A
**314K**	Common kestrel	*Falco tinnunculus*	Unknown	N/A	Cloaca	neg.	n.d.	n.d.	N/A
**315C**	Common kestrel	*Falco tinnunculus*	4704 Wolfisberg	BE	Choana	neg.	n.d.	n.d.	N/A
**315K**	Common kestrel	*Falco tinnunculus*	4704 Wolfisberg	BE	Cloaca	neg.	n.d.	n.d.	N/A
**316C**	Common kestrel	*Falco tinnunculus*	Unknown	N/A	Choana	neg.	n.d.	n.d.	N/A
**316K**	Common kestrel	*Falco tinnunculus*	Unknown	N/A	Cloaca	neg.	n.d.	n.d.	N/A
**317C**	Red kite	*Milvus milvus*	Unknown	N/A	Choana	neg.	n.d.	n.d.	N/A
**317K**	Red kite	*Milvus milvus*	Unknown	N/A	Cloaca	neg.	n.d.	n.d.	N/A
**318C**	Common buzzard	*Buteo buteo*	Unknown	N/A	Choana	neg.	n.d.	n.d.	N/A
**318K**	Common buzzard	*Buteo buteo*	Unknown	N/A	Cloaca	neg.	n.d.	n.d.	N/A
**319C**	Common buzzard	*Buteo buteo*	Unknown	N/A	Choana	neg.	n.d.	n.d.	N/A
**319K**	Common buzzard	*Buteo buteo*	Unknown	N/A	Cloaca	neg.	n.d.	n.d.	N/A
**320C**	Common buzzard	*Buteo buteo*	Unknown	N/A	Choana	neg.	n.d.	n.d.	N/A
**320K**	Common buzzard	*Buteo buteo*	Unknown	N/A	Cloaca	neg.	n.d.	n.d.	N/A
**321C**	Tawny owl	*Strix aluco*	Unknown	N/A	Choana	neg.	n.d.	n.d.	N/A
**321K**	Tawny owl	*Strix aluco*	Unknown	N/A	Cloaca	neg.	n.d.	n.d.	N/A
**323C**	Eurasian magpie	*Pica pica*	6234 Triengen	LU	Choana	neg.	n.d.	n.d.	N/A
**323K**	Eurasian magpie	*Pica pica*	6234 Triengen	LU	Cloaca	neg.	n.d.	n.d.	N/A
**329Kot**	Common buzzard	*Buteo buteo*	Unknown	N/A	Feces	neg.	n.d.	n.d.	N/A
**330Kot**	Common kestrel	*Falco tinnunculus*	Unknown	N/A	Feces	neg.	n.d.	n.d.	N/A
**339C**	Common kestrel	*Falco tinnunculus*	8918 Unterlunkhofen	AG	Choana	neg.	n.d.	n.d.	N/A
**339K**	Common kestrel	*Falco tinnunculus*	8918 Unterlunkhofen	AG	Cloaca	neg.	n.d.	n.d.	N/A
**339Kot**	Common kestrel	*Falco tinnunculus*	8918 Unterlunkhofen	AG	Feces	neg.	n.d.	n.d.	N/A
**348C**	Carrion crow	*Corvus corone*	4802 Strengelbach	AG	Choana	neg.	n.d.	n.d.	N/A

**348K**	Carrion crow	*Corvus corone*	4802 Strengelbach	AG	Cloaca	neg.	n.d.	n.d.	N/A
**371C**	Common kestrel	*Falco tinnunculus*	8903 Birmensdorf	AG	Choana	neg.	n.d.	n.d.	N/A
**371K**	Common kestrel	*Falco tinnunculus*	8903 Birmensdorf	AG	Cloaca	neg.	n.d.	n.d.	N/A
**373Kot**	Carrion crow	*Corvus corone*	6216 Mauensee	LU	Feces	neg.	n.d.	n.d.	N/A
**377C**	Common kestrel	*Falco tinnunculus*	6074 Giswil	OW	Choana	neg.	n.d.	n.d.	N/A
**377K**	Common kestrel	*Falco tinnunculus*	6074 Giswil	OW	Cloaca	neg.	n.d.	n.d.	N/A
**378C**	Common buzzard	*Buteo buteo*	6062 Oberwilen	OW	Choana	neg.	n.d.	n.d.	N/A
**378K**	Common buzzard	*Buteo buteo*	6062 Oberwilen	OW	Cloaca	neg.	n.d.	n.d.	N/A
**379Kot**	Eurasian hobby	*Falco subbuteo*	4450 Sissach	BL	Feces	neg.	n.d.	n.d.	N/A
**381C**	Red kite	*Milvus milvus*	6045 Meggen	LU	Choana	neg.	n.d.	n.d.	N/A
**381K**	Red kite	*Milvus milvus*	6045 Meggen	LU	Cloaca	neg.	n.d.	n.d.	N/A
**382C**	Eurasian magpie	*Pica pica*	6204 Sempach	LU	Choana	neg.	n.d.	n.d.	N/A
**382K**	Eurasian magpie	*Pica pica*	6204 Sempach	LU	Cloaca	neg.	n.d.	n.d.	N/A
**383Kot**	Common buzzard	*Buteo buteo*	5643 Sins	AG	Feces	neg.	n.d.	n.d.	N/A
**385Kot**	Red kite	*Milvus milvus*	5632 Buttwil	AG	Feces	neg.	n.d.	n.d.	N/A
**388C**	Eurasian magpie	*Pica pica*	6284 Sulz	LU	Choana	neg.	n.d.	n.d.	N/A
**388K**	Eurasian magpie	*Pica pica*	6284 Sulz	LU	Cloaca	neg.	n.d.	n.d.	N/A
**388Kot**	Eurasian magpie	*Pica pica*	6284 Sulz	LU	Feces	neg.	n.d.	n.d.	N/A
**402C**	Black kite	*Milvus migrans*	6122 Menznau	LU	Choana	neg.	n.d.	n.d.	N/A
**402K**	Black kite	*Milvus migrans*	6122 Menznau	LU	Cloaca	neg.	n.d.	n.d.	N/A
**402Kot**	Black kite	*Milvus migrans*	6122 Menznau	LU	Feces	neg.	n.d.	n.d.	N/A
**412Kot**	Eurasian magpie	*Pica pica*	6038 Gisikon	OW	Feces	neg.	n.d.	n.d.	N/A
**425C**	Tawny owl	*Strix aluco*	5728 Gontenschwil	AG	Choana	neg.	n.d.	n.d.	N/A
**425K**	Tawny owl	*Strix aluco*	5728 Gontenschwil	AG	Cloaca	neg.	n.d.	n.d.	N/A
**425Kot**	Tawny owl	*Strix aluco*	5728 Gontenschwil	AG	Feces	neg.	n.d.	n.d.	N/A
**443C**	Eurasian eagle-owl	*Asio otus*	6126 Daiwil	LU	Choana	neg.	n.d.	n.d.	N/A
**443K**	Eurasian eagle-owl	*Asio otus*	6126 Daiwil	LU	Cloaca	neg.	n.d.	n.d.	N/A
**452C**	Eurasian sparrowhawk	*Accipiter nisus*	6476 Altdorf	UR	Choana	neg.	n.d.	n.d.	N/A
**452K**	Eurasian sparrowhawk	*Accipiter nisus*	6476 Altdorf	UR	Cloaca	neg.	n.d.	n.d.	N/A
**452Kot**	Eurasian sparrowhawk	*Accipiter nisus*	6476 Altdorf	UR	Feces	neg.	n.d.	n.d.	N/A
**454C**	Common buzzard	*Buteo buteo*	6234 Triengen	LU	Choana	neg.	n.d.	n.d.	N/A
**454K**	Common buzzard	*Buteo buteo*	6234 Triengen	LU	Cloaca	neg.	n.d.	n.d.	N/A
**460C**	Barn owl	*Tyto alba*	6203 Sempach-Station	LU	Choana	neg.	n.d.	n.d.	N/A
**460K**	Barn owl	*Tyto alba*	6203 Sempach-Station	LU	Cloaca	neg.	n.d.	n.d.	N/A
**472C**	Barn owl	*Tyto alba*	1678 Siviriez	FR	Choana	neg.	n.d.	n.d.	N/A
**472K**	Barn owl	*Tyto alba*	1678 Siviriez	FR	Cloaca	neg.	n.d.	n.d.	N/A
**473C**	Red kite	*Milvus milvus*	5728 Gontenschwil	AG	Choana	neg.	n.d.	n.d.	N/A
**473K**	Red kite	*Milvus milvus*	5728 Gontenschwil	AG	Cloaca	neg.	n.d.	n.d.	N/A
**474C**	Tawny owl	*Strix aluco*	6276 Hohenrain	LU	Choana	neg.	n.d.	n.d.	N/A
**474K**	Tawny owl	*Strix aluco*	6276 Hohenrain	LU	Cloaca	neg.	n.d.	n.d.	N/A
**476C**	Black kite	*Milvus migrans*	6032 Emmen	LU	Choana	neg.	n.d.	n.d.	N/A
**476K**	Black kite	*Milvus migrans*	6032 Emmen	LU	Cloaca	neg.	n.d.	n.d.	N/A
**477C**	Barn owl	*Tyto alba*	6204 Sempach	LU	Choana	neg.	n.d.	n.d.	N/A

**477K**	Barn owl	*Tyto alba*	6204 Sempach	LU	Cloaca	neg.	n.d.	n.d.	N/A
**478C**	Common buzzard	*Buteo buteo*	6234 Schlierbach	LU	Choana	neg.	n.d.	n.d.	N/A
**478K**	Common buzzard	*Buteo buteo*	6234 Schlierbach	LU	Cloaca	neg.	n.d.	n.d.	N/A
**479C**	Eurasian sparrowhawk	*Accipiter nisus*	4805 Brittnau	AG	Choana	neg.	n.d.	n.d.	N/A
**479K**	Eurasian sparrowhawk	*Accipiter nisus*	4805 Brittnau	AG	Cloaca	neg.	n.d.	n.d.	N/A
**480C**	Barn owl	*Tyto alba*	5645 Fenkrieden	AG	Choana	neg.	n.d.	n.d.	N/A
**480K**	Barn owl	*Tyto alba*	5645 Fenkrieden	AG	Cloaca	neg.	n.d.	n.d.	N/A
**481C**	Common kestrel	*Falco tinnunculus*	6023 Rothenburg	LU	Choana	neg.	n.d.	n.d.	N/A
**481K**	Common kestrel	*Falco tinnunculus*	6023 Rothenburg	LU	Cloaca	neg.	n.d.	n.d.	N/A
**482C**	Common kestrel	*Falco tinnunculus*	6055 Alpnach	OW	Choana	neg.	n.d.	n.d.	N/A
**482K**	Common kestrel	*Falco tinnunculus*	6055 Alpnach	OW	Cloaca	neg.	n.d.	n.d.	N/A
**483C**	Montagu's harrier	*Circus pygargus*	3860 Meiringen	BE	Choana	neg.	n.d.	n.d.	N/A
**483K**	Montagu's harrier	*Circus pygargus*	3860 Meiringen	BE	Cloaca	neg.	n.d.	n.d.	N/A
**484C**	Eurasian sparrowhawk	*Accipiter nisus*	6212 St. Erhard	LU	Choana	neg.	n.d.	n.d.	N/A
**484K**	Eurasian sparrowhawk	*Accipiter nisus*	6212 St. Erhard	LU	Cloaca	neg.	n.d.	n.d.	N/A
**485C**	Common kestrel	*Falco tinnunculus*	6205 Eich	LU	Choana	neg.	n.d.	n.d.	N/A
**485K**	Common kestrel	*Falco tinnunculus*	6205 Eich	LU	Cloaca	neg.	n.d.	n.d.	N/A
**486C**	Eurasian magpie	*Pica pica*	6383 Dallenwil	NW	Choana	neg.	n.d.	n.d.	N/A
**486K**	Eurasian magpie	*Pica pica*	6383 Dallenwil	NW	Cloaca	neg.	n.d.	n.d.	N/A
**487Kot**	Carrion crow	*Corvus corone*	6032 Emmen	LU	Feces	pos. (36.2)	neg.	neg.	Not identified
**488C**	Eurasian sparrowhawk	*Accipiter nisus*	6210 Sursee	LU	Choana	neg.	n.d.	n.d.	N/A
**488K**	Eurasian sparrowhawk	*Accipiter nisus*	6210 Sursee	LU	Cloaca	neg.	n.d.	n.d.	N/A
**489C**	Common buzzard	*Buteo buteo*	6026 Rain	LU	Choana	neg.	n.d.	n.d.	N/A
**489K**	Common buzzard	*Buteo buteo*	6026 Rain	LU	Cloaca	neg.	n.d.	n.d.	N/A
**490C**	Carrion crow	*Corvus corone*	6014 Littau	LU	Choana	neg.	n.d.	n.d.	N/A
**490K**	Carrion crow	*Corvus corone*	6014 Littau	LU	Cloaca	neg.	n.d.	n.d.	N/A
**491C**	Common buzzard	*Buteo buteo*	6204 Sempach	LU	Choana	neg.	n.d.	n.d.	N/A
**491K**	Common buzzard	*Buteo buteo*	6204 Sempach	LU	Cloaca	neg.	n.d.	n.d.	N/A
**492C**	Eurasian jay	*Garrulus glandarius*	4125 Riehen	BL	Choana	neg.	n.d.	n.d.	N/A
**492K**	Eurasian jay	*Garrulus glandarius*	4125 Riehen	BL	Cloaca	neg.	n.d.	n.d.	N/A
**493C**	Eurasian sparrowhawk	*Accipiter nisus*	Unknown	N/A	Choana	neg.	n.d.	n.d.	N/A
**493K**	Eurasian sparrowhawk	*Accipiter nisus*	Unknown	N/A	Cloaca	neg.	n.d.	n.d.	N/A
**494C**	Common buzzard	*Buteo buteo*	8500 Frauenfeld	TG	Choana	neg.	n.d.	n.d.	N/A
**494K**	Common buzzard	*Buteo buteo*	8500 Frauenfeld	TG	Cloaca	neg.	n.d.	n.d.	N/A
**495C**	Common buzzard	*Buteo buteo*	8804 Wädenswil	ZH	Choana	neg.	n.d.	n.d.	N/A
**495K**	Common buzzard	*Buteo buteo*	8804 Wädenswil	ZH	Cloaca	neg.	n.d.	n.d.	N/A
**496C**	Common buzzard	*Buteo buteo*	8262 Ramsen	SH	Choana	neg.	n.d.	n.d.	N/A
**496K**	Common buzzard	*Buteo buteo*	8262 Ramsen	SH	Cloaca	neg.	n.d.	n.d.	N/A
**497C**	Common buzzard	*Buteo buteo*	8001 Zürich	ZH	Choana	neg.	n.d.	n.d.	N/A
**497K**	Common buzzard	*Buteo buteo*	8001 Zürich	ZH	Cloaca	neg.	n.d.	n.d.	N/A
**498C**	Common buzzard	*Buteo buteo*	Unknown	N/A	Choana	neg.	n.d.	n.d.	N/A
**498K**	Common buzzard	*Buteo buteo*	Unknown	N/A	Cloaca	neg.	n.d.	n.d.	N/A
**499C**	Common buzzard	*Buteo buteo*	8416 Flaach	ZH	Choana	neg.	n.d.	n.d.	N/A

**499K**	Common buzzard	*Buteo buteo*	8416 Flaach	ZH	Cloaca	neg.	n.d.	n.d.	N/A
**500C**	Common buzzard	*Buteo buteo*	8254 Basadingen	TG	Choana	neg.	n.d.	n.d.	N/A
**500K**	Common buzzard	*Buteo buteo*	8254 Basadingen	TG	Cloaca	neg.	n.d.	n.d.	N/A
**501C**	Common buzzard	*Buteo buteo*	8400 Winterthur	ZH	Choana	neg.	n.d.	n.d.	N/A
**501K**	Common buzzard	*Buteo buteo*	8400 Winterthur	ZH	Cloaca	neg.	n.d.	n.d.	N/A
**502C**	Common buzzard	*Buteo buteo*	Unknown	N/A	Choana	neg.	n.d.	n.d.	N/A
**502K**	Common buzzard	*Buteo buteo*	Unknown	N/A	Cloaca	neg.	n.d.	n.d.	N/A
**503C**	Common buzzard	*Buteo buteo*	6344 Meierskappel	LU	Choana	neg.	n.d.	n.d.	N/A
**503K**	Common buzzard	*Buteo buteo*	6344 Meierskappel	LU	Cloaca	neg.	n.d.	n.d.	N/A
**504C**	Common buzzard	*Buteo buteo*	6032 Emmen	LU	Choana	neg.	n.d.	n.d.	N/A
**504K**	Common buzzard	*Buteo buteo*	6032 Emmen	LU	Cloaca	neg.	n.d.	n.d.	N/A
**505C**	Common buzzard	*Buteo buteo*	5467 Fisibach	AG	Choana	neg.	n.d.	n.d.	N/A
**505K**	Common buzzard	*Buteo buteo*	5467 Fisibach	AG	Cloaca	neg.	n.d.	n.d.	N/A
**506C**	Common buzzard	*Buteo buteo*	8422 Pfungen	ZH	Choana	neg.	n.d.	n.d.	N/A
**506K**	Common buzzard	*Buteo buteo*	8422 Pfungen	ZH	Cloaca	neg.	n.d.	n.d.	N/A
**507C**	Common buzzard	*Buteo buteo*	8590 Romanshorn	TG	Choana	neg.	n.d.	n.d.	N/A
**507K**	Common buzzard	*Buteo buteo*	8590 Romanshorn	TG	Cloaca	neg.	n.d.	n.d.	N/A
**508C**	Common buzzard	*Buteo buteo*	8058 Flughafen-Zürich	ZH	Choana	neg.	n.d.	n.d.	N/A
**508K**	Common buzzard	*Buteo buteo*	8058 Flughafen-Zürich	ZH	Cloaca	neg.	n.d.	n.d.	N/A
**509C**	Common buzzard	*Buteo buteo*	8340 Hinwil	ZH	Choana	neg.	n.d.	n.d.	N/A
**509K**	Common buzzard	*Buteo buteo*	8340 Hinwil	ZH	Cloaca	neg.	n.d.	n.d.	N/A
**510C**	Common buzzard	*Buteo buteo*	8488 Turbenthal	ZH	Choana	neg.	n.d.	n.d.	N/A
**510K**	Common buzzard	*Buteo buteo*	8488 Turbenthal	ZH	Cloaca	neg.	n.d.	n.d.	N/A
**511C**	Common buzzard	*Buteo buteo*	Unknown	N/A	Choana	pos. (38.3)	neg.	neg.	*C. psittaci* M56
**511K**	Common buzzard	*Buteo buteo*	Unknown	N/A	Cloaca	pos. (35.2)	neg.	neg.	Not identified
**512C**	Common buzzard	*Buteo buteo*	8185 Winkel	ZH	Choana	neg.	n.d.	n.d.	N/A
**512K**	Common buzzard	*Buteo buteo*	8185 Winkel	ZH	Cloaca	neg.	n.d.	n.d.	N/A
**513C**	Red kite	*Milvus milvus*	8700 Küsnacht	ZH	Choana	neg.	n.d.	n.d.	N/A
**513K**	Red kite	*Milvus milvus*	8700 Küsnacht	ZH	Cloaca	neg.	n.d.	n.d.	N/A
**514C**	Common buzzard	*Buteo buteo*	8046 Zürich Affoltern	ZH	Choana	neg.	n.d.	n.d.	N/A
**514K**	Common buzzard	*Buteo buteo*	8046 Zürich Affoltern	ZH	Cloaca	neg.	n.d.	n.d.	N/A
**515C**	Common buzzard	*Buteo buteo*	8460 Marthalen	ZH	Choana	neg.	n.d.	n.d.	N/A
**515K**	Common buzzard	*Buteo buteo*	8460 Marthalen	ZH	Cloaca	neg.	n.d.	n.d.	N/A
**516C**	Common buzzard	*Buteo buteo*	8610 Uster	ZH	Choana	neg.	n.d.	n.d.	N/A
**516K**	Common buzzard	*Buteo buteo*	8610 Uster	ZH	Cloaca	neg.	n.d.	n.d.	N/A
**517C**	Common buzzard	*Buteo buteo*	8535 Herdern	TG	Choana	neg.	n.d.	n.d.	N/A
**517K**	Common buzzard	*Buteo buteo*	8535 Herdern	TG	Cloaca	neg.	n.d.	n.d.	N/A
**518C**	Common buzzard	*Buteo buteo*	8460 Marthalen	ZH	Choana	neg.	n.d.	n.d.	N/A
**518K**	Common buzzard	*Buteo buteo*	8460 Marthalen	ZH	Cloaca	neg.	n.d.	n.d.	N/A
**519C**	Common buzzard	*Buteo buteo*	9400 Rorschach	SG	Choana	neg.	n.d.	n.d.	N/A
**519K**	Common buzzard	*Buteo buteo*	9400 Rorschach	SG	Cloaca	neg.	n.d.	n.d.	N/A
**520C**	Common buzzard	*Buteo buteo*	Unknown	ZH	Choana	neg.	n.d.	n.d.	N/A
**520K**	Common buzzard	*Buteo buteo*	Unknown	ZH	Cloaca	neg.	n.d.	n.d.	N/A

**521C**	Eurasian sparrowhawk	*Accipiter nisus*	8409 Winterthur	ZH	Choana	neg.	n.d.	n.d.	N/A
**521K**	Eurasian sparrowhawk	*Accipiter nisus*	8409 Winterthur	ZH	Cloaca	neg.	n.d.	n.d.	N/A
**522C**	Common buzzard	*Buteo buteo*	8902 Urdorf	ZH	Choana	neg.	n.d.	n.d.	N/A
**522K**	Common buzzard	*Buteo buteo*	8902 Urdorf	ZH	Cloaca	neg.	n.d.	n.d.	N/A
**523C**	Common buzzard	*Buteo buteo*	8315 Tagelswangen	ZH	Choana	neg.	n.d.	n.d.	N/A
**523K**	Common buzzard	*Buteo buteo*	8315 Tagelswangen	ZH	Cloaca	neg.	n.d.	n.d.	N/A
**524C**	Common kestrel	*Falco tinnunculus*	8196 Wil (Rafz)	ZH	Choana	neg.	n.d.	n.d.	N/A
**524K**	Common kestrel	*Falco tinnunculus*	8196 Wil (Rafz)	ZH	Cloaca	neg.	n.d.	n.d.	N/A
**525C**	Carrion crow	*Corvus corone*	6174 Sörenberg	LU	Choana	neg.	n.d.	n.d.	N/A
**525K**	Carrion crow	*Corvus corone*	6174 Sörenberg	LU	Cloaca	neg.	n.d.	n.d.	N/A
**526C**	Common buzzard	*Buteo buteo*	5225 Bözberg	AG	Choana	neg.	n.d.	n.d.	N/A
**526K**	Common buzzard	*Buteo buteo*	5225 Bözberg	AG	Cloaca	neg.	n.d.	n.d.	N/A
**527Kot**	Common buzzard	*Buteo buteo*	5712 Beinwil am See	AG	Feces	neg.	n.d.	n.d.	N/A
**528Kot**	Common buzzard	*Buteo buteo*	6000 Luzern	LU	Feces	neg.	n.d.	n.d.	N/A
**529C**	Common buzzard	*Buteo buteo*	6252 Dagmersellen	LU	Choana	neg.	n.d.	n.d.	N/A
**529K**	Common buzzard	*Buteo buteo*	6252 Dagmersellen	LU	Cloaca	neg.	n.d.	n.d.	N/A
**530Kot**	Common buzzard	*Buteo buteo*	6204 Sempach	LU	Feces	neg.	n.d.	n.d.	N/A
**531Kot**	Common buzzard	*Buteo buteo*	6333 Hünenberg	ZG	Feces	neg.	n.d.	n.d.	N/A
**532C**	Common buzzard	*Buteo buteo*	6025 Neudorf	LU	Choana	neg.	n.d.	n.d.	N/A
**532K**	Common buzzard	*Buteo buteo*	6025 Neudorf	LU	Cloaca	neg.	n.d.	n.d.	N/A
**533C**	Common buzzard	*Buteo buteo*	6023 Rothenburg	LU	Choana	neg.	n.d.	n.d.	N/A
**533K**	Common buzzard	*Buteo buteo*	6023 Rothenburg	LU	Cloaca	neg.	n.d.	n.d.	N/A
**534C**	Common buzzard	*Buteo buteo*	8187 Weiach	ZH	Choana	neg.	n.d.	n.d.	N/A
**534K**	Common buzzard	*Buteo buteo*	8187 Weiach	ZH	Cloaca	neg.	n.d.	n.d.	N/A
**535C**	Common buzzard	*Buteo buteo*	9548 Matzingen	TG	Choana	neg.	n.d.	n.d.	N/A
**535K**	Common buzzard	*Buteo buteo*	9548 Matzingen	TG	Cloaca	neg.	n.d.	n.d.	N/A
**536C**	Eurasian sparrowhawk	*Accipiter nisus*	8570 Ottenberg	TG	Choana	neg.	n.d.	n.d.	N/A
**536K**	Eurasian sparrowhawk	*Accipiter nisus*	8570 Ottenberg	TG	Cloaca	neg.	n.d.	n.d.	N/A
**537C**	Common buzzard	*Buteo buteo*	8610 Uster	ZH	Choana	neg.	n.d.	n.d.	N/A
**537K**	Common buzzard	*Buteo buteo*	8610 Uster	ZH	Cloaca	neg.	n.d.	n.d.	N/A
**538C**	Common buzzard	*Buteo buteo*	8156 Oberhasli	ZH	Choana	neg.	n.d.	n.d.	N/A
**538K**	Common buzzard	*Buteo buteo*	8156 Oberhasli	ZH	Cloaca	neg.	n.d.	n.d.	N/A
**539C**	Common buzzard	*Buteo buteo*	8253 Diessenhofen	TG	Choana	neg.	n.d.	n.d.	N/A
**539K**	Common buzzard	*Buteo buteo*	8253 Diessenhofen	TG	Cloaca	neg.	n.d.	n.d.	N/A
**540C**	Common buzzard	*Buteo buteo*	8225 Siblingen	SH	Choana	neg.	n.d.	n.d.	N/A
**540K**	Common buzzard	*Buteo buteo*	8225 Siblingen	SH	Cloaca	neg.	n.d.	n.d.	N/A
**541C**	Common buzzard	*Buteo buteo*	8400 Winterthur	ZH	Choana	neg.	n.d.	n.d.	N/A
**541K**	Common buzzard	*Buteo buteo*	8400 Winterthur	ZH	Cloaca	neg.	n.d.	n.d.	N/A
**542C**	Common buzzard	*Buteo buteo*	8226 Schleitheim	SH	Choana	neg.	n.d.	n.d.	N/A
**542K**	Common buzzard	*Buteo buteo*	8226 Schleitheim	SH	Cloaca	neg.	n.d.	n.d.	N/A
**543C**	Common buzzard	*Buteo buteo*	8185 Winkel	ZH	Choana	neg.	n.d.	n.d.	N/A
**543K**	Common buzzard	*Buteo buteo*	8185 Winkel	ZH	Cloaca	neg.	n.d.	n.d.	N/A
**544C**	Common buzzard	*Buteo buteo*	8416 Flaach	ZH	Choana	neg.	n.d.	n.d.	N/A

**544K**	Common buzzard	*Buteo buteo*	8416 Flaach	ZH	Cloaca	neg.	n.d.	n.d.	N/A
**545C**	Common buzzard	*Buteo buteo*	5634 Merenschwand	AG	Choana	neg.	n.d.	n.d.	N/A
**545K**	Common buzzard	*Buteo buteo*	5634 Merenschwand	AG	Cloaca	neg.	n.d.	n.d.	N/A
**546C**	Common buzzard	*Buteo buteo*	9000 St. Gallen	SG	Choana	neg.	n.d.	n.d.	N/A
**546K**	Common buzzard	*Buteo buteo*	9000 St. Gallen	SG	Cloaca	neg.	n.d.	n.d.	N/A
**547C**	Common buzzard	*Buteo buteo*	8247 Thayngen	SH	Choana	neg.	n.d.	n.d.	N/A
**547K**	Common buzzard	*Buteo buteo*	8247 Thayngen	SH	Cloaca	neg.	n.d.	n.d.	N/A
**548K**	Common buzzard	*Buteo buteo*	8926 Kappel am Albis	ZH	Cloaca	neg.	n.d.	n.d.	N/A
**549C**	Common buzzard	*Buteo buteo*	8902 Urdorf	ZH	Choana	neg.	n.d.	n.d.	N/A
**549K**	Common buzzard	*Buteo buteo*	8902 Urdorf	ZH	Cloaca	neg.	n.d.	n.d.	N/A
**550C**	Common buzzard	*Buteo buteo*	8136 Gattikon	ZH	Choana	neg.	n.d.	n.d.	N/A
**550K**	Common buzzard	*Buteo buteo*	8136 Gattikon	ZH	Cloaca	neg.	n.d.	n.d.	N/A
**551C**	Common buzzard	*Buteo buteo*	9506 Lommis	TG	Choana	neg.	n.d.	n.d.	N/A
**551K**	Common buzzard	*Buteo buteo*	9506 Lommis	TG	Cloaca	neg.	n.d.	n.d.	N/A
**552C**	Common buzzard	*Buteo buteo*	8265 Mammern	TG	Choana	neg.	n.d.	n.d.	N/A
**552K**	Common buzzard	*Buteo buteo*	8265 Mammern	TG	Cloaca	neg.	n.d.	n.d.	N/A
**553C**	Red kite	*Milvus milvus*	8108 Dällikon	ZH	Choana	neg.	n.d.	n.d.	N/A
**553K**	Red kite	*Milvus milvus*	8108 Dällikon	ZH	Cloaca	neg.	n.d.	n.d.	N/A
**554C**	Common buzzard	*Buteo buteo*	5313 Klingnau	AG	Choana	neg.	n.d.	n.d.	N/A
**554K**	Common buzzard	*Buteo buteo*	5313 Klingnau	AG	Cloaca	neg.	n.d.	n.d.	N/A
**555C**	Common buzzard	*Buteo buteo*	8280 Kreuzlingen	TG	Choana	neg.	n.d.	n.d.	N/A
**555K**	Common buzzard	*Buteo buteo*	8280 Kreuzlingen	TG	Cloaca	neg.	n.d.	n.d.	N/A
**556C**	Common buzzard	*Buteo buteo*	8416 Flaach	ZH	Choana	pos. (30.7)	neg.	neg.	*C. psittaci* M56
**556K**	Common buzzard	*Buteo buteo*	8416 Flaach	ZH	Cloaca	pos. (14.1)	neg.	neg.	*C. psittaci* M56
**557C**	Common buzzard	*Buteo buteo*	8305 Dietikon	ZH	Choana	pos. (37.1)	neg.	neg.	Not identified
**557K**	Common buzzard	*Buteo buteo*	8305 Dietikon	ZH	Cloaca	pos. (36.9)	neg.	neg.	Not identified
**558C**	Common buzzard	*Buteo buteo*	8320 Fehraltdorf	ZH	Choana	pos. (38.8)	neg.	neg.	Not identified
**558K**	Common buzzard	*Buteo buteo*	8320 Fehraltdorf	ZH	Cloaca	pos. (41.3)	neg.	neg.	Not identified
**559C**	Common buzzard	*Buteo buteo*	8165 Oberweningen	ZH	Choana	neg.	n.d.	n.d.	N/A
**559K**	Common buzzard	*Buteo buteo*	8165 Oberweningen	ZH	Cloaca	neg.	n.d.	n.d.	N/A
**560Kot**	Common buzzard	*Buteo buteo*	6242 Wauwil	LU	Feces	neg.	n.d.	n.d.	N/A
**561C**	Common buzzard	*Buteo buteo*	6016 Hellbühl	LU	Choana	neg.	n.d.	n.d.	N/A
**561K**	Common buzzard	*Buteo buteo*	6016 Hellbühl	LU	Cloaca	neg.	n.d.	n.d.	N/A
**562C**	Common buzzard	*Buteo buteo*	Unknown	N/A	Choana	neg.	n.d.	n.d.	N/A
**562K**	Common buzzard	*Buteo buteo*	Unknown	N/A	Cloaca	neg.	n.d.	n.d.	N/A
**562Kot**	Common buzzard	*Buteo buteo*	Unknown	N/A	Feces	neg.	n.d.	n.d.	N/A
**563C**	Common buzzard	*Buteo buteo*	8460 Marthalen	ZH	Choana	neg.	n.d.	n.d.	N/A
**563K**	Common buzzard	*Buteo buteo*	8460 Marthalen	ZH	Cloaca	neg.	n.d.	n.d.	N/A
**564Kot**	Common buzzard	*Buteo buteo*	6014 Littau	LU	Feces	neg.	n.d.	n.d.	N/A
**565C**	Carrion crow	*Corvus corone*	4800 Zofingen	AG	Choana	pos. (27.9)	neg.	neg.	*C. psittaci* 1V
**565K**	Carrion crow	*Corvus corone*	4800 Zofingen	AG	Cloaca	neg.	n.d.	n.d.	N/A
**565Kot**	Carrion crow	*Corvus corone*	4800 Zofingen	AG	Feces	neg.	n.d.	n.d.	N/A
**566C**	Common buzzard	*Buteo buteo*	6055 Alpnach	OW	Choana	pos. (29.8)	neg.	neg.	*C. psittaci* 1V

**566K**	Common buzzard	*Buteo buteo*	6055 Alpnach	OW	Cloaca	neg.	n.d.	n.d.	N/A
**567Kot**	Long-eared owl	*Asio otus*	6042 Dietwil	AG	Feces	neg.	n.d.	n.d.	N/A
**568C**	Common buzzard	*Buteo buteo*	6122 Menznau	LU	Choana	neg.	n.d.	n.d.	N/A
**568K**	Common buzzard	*Buteo buteo*	6122 Menznau	LU	Cloaca	neg.	n.d.	n.d.	N/A
**569C**	Common buzzard	*Buteo buteo*	6211 Buchs	LU	Choana	neg.	n.d.	n.d.	N/A
**569K**	Common buzzard	*Buteo buteo*	6211 Buchs	LU	Cloaca	neg.	n.d.	n.d.	N/A
**570C**	Common buzzard	*Buteo buteo*	6253 Dagmersellen	LU	Choana	neg.	n.d.	n.d.	N/A
**570K**	Common buzzard	*Buteo buteo*	6253 Dagmersellen	LU	Cloaca	neg.	n.d.	n.d.	N/A
**571C**	Carrion crow	*Corvus corone*	6285 Hitzkirch	LU	Choana	neg.	n.d.	n.d.	N/A
**571K**	Carrion crow	*Corvus corone*	6285 Hitzkirch	LU	Cloaca	neg.	n.d.	n.d.	N/A
**572C**	Red kite	*Milvus milvus*	6207 Nottwil	LU	Choana	neg.	n.d.	n.d.	N/A
**572K**	Red kite	*Milvus milvus*	6207 Nottwil	LU	Cloaca	neg.	n.d.	n.d.	N/A
**573C**	Eurasian magpie	*Pica pica*	6038 Gisikon	LU	Choana	neg.	n.d.	n.d.	N/A
**573K**	Eurasian magpie	*Pica pica*	6038 Gisikon	LU	Cloaca	neg.	n.d.	n.d.	N/A
**574C**	Common buzzard	*Buteo buteo*	6207 Nottwil	LU	Choana	neg.	n.d.	n.d.	N/A
**574K**	Common buzzard	*Buteo buteo*	6207 Nottwil	LU	Cloaca	neg.	n.d.	n.d.	N/A
**575K**	Common buzzard	*Buteo buteo*	6233 Büron	LU	Cloaca	neg.	n.d.	n.d.	N/A
**576C**	Common buzzard	*Buteo buteo*	6287 Aesch	LU	Choana	neg.	n.d.	n.d.	N/A
**576K**	Common buzzard	*Buteo buteo*	6287 Aesch	LU	Cloaca	neg.	n.d.	n.d.	N/A
**577C**	Carrion crow	*Corvus corone*	Luzern	LU	Choana	neg.	n.d.	n.d.	N/A
**577K**	Carrion crow	*Corvus corone*	Luzern	LU	Cloaca	neg.	n.d.	n.d.	N/A
**578C**	Common kestrel	*Falco tinnunculus*	Reiden	LU	Choana	neg.	n.d.	n.d.	N/A
**578K**	Common kestrel	*Falco tinnunculus*	Reiden	LU	Cloaca	neg.	n.d.	n.d.	N/A
**579Kot**	Tawny owl	*Strix aluco*	6283 Baldegg	LU	Feces	neg.	n.d.	n.d.	N/A
**580C**	Red kite	*Milvus milvus*	6218 Ettiswil	LU	Choana	neg.	n.d.	n.d.	N/A
**580K**	Red kite	*Milvus milvus*	6218 Ettiswil	LU	Cloaca	neg.	n.d.	n.d.	N/A
**581C**	Rook	*Corvus frugilegus*	5712 Beinwil am See	AG	Choana	neg.	n.d.	n.d.	N/A
**581K**	Rook	*Corvus frugilegus*	5712 Beinwil am See	AG	Cloaca	neg.	n.d.	n.d.	N/A
**581Kot**	Rook	*Corvus frugilegus*	5712 Beinwil am See	AG	Feces	neg.	n.d.	n.d.	N/A
**582Kot**	Eurasian magpie	*Pica pica*	6275 Ballwil	LU	Feces	neg.	n.d.	n.d.	N/A
**583Kot**	Eurasian magpie	*Pica pica*	6275 Ballwil	LU	Feces	neg.	n.d.	n.d.	N/A
**584Kot**	Eurasian magpie	*Pica pica*	6276 Ballwil	LU	Feces	neg.	n.d.	n.d.	N/A
**585Kot**	Eurasian magpie	*Pica pica*	6275 Ballwil	LU	Feces	neg.	n.d.	n.d.	N/A
**586Kot**	Eurasian magpie	*Pica pica*	6278 Ballwil	LU	Feces	neg.	n.d.	n.d.	N/A
**587C**	Long-eared owl	*Asio otus*	6042 Dietwil	AG	Choana	neg.	n.d.	n.d.	N/A
**587K**	Long-eared owl	*Asio otus*	6042 Dietwil	AG	Cloaca	neg.	n.d.	n.d.	N/A
**588Kot**	Eurasian magpie	*Pica pica*	Luzern	LU	Feces	neg.	n.d.	n.d.	N/A
**589C**	Common buzzard	*Buteo buteo*	5610 Wohlen	AG	Choana	neg.	n.d.	n.d.	N/A
**589K**	Common buzzard	*Buteo buteo*	5610 Wohlen	AG	Cloaca	neg.	n.d.	n.d.	N/A
**590C**	Red kite	*Milvus milvus*	6275 Ballwil	LU	Choana	neg.	n.d.	n.d.	N/A
**590K**	Red kite	*Milvus milvus*	6275 Ballwil	LU	Cloaca	neg.	n.d.	n.d.	N/A
**591Kot**	Common buzzard	*Buteo buteo*	Zug	ZG	Feces	neg.	n.d.	n.d.	N/A
**592C**	Eurasian sparrowhawk	*Accipiter nisus*	6210 Sursee	LU	Choana	neg.	n.d.	n.d.	N/A

**592K**	Eurasian sparrowhawk	*Accipiter nisus*	6210 Sursee	LU	Cloaca	neg.	n.d.	n.d.	N/A
**593Kot**	Carrion crow	*Corvus corone*	Unknown	N/A	Feces	neg.	n.d.	n.d.	N/A
**594C**	Carrion crow	*Corvus corone*	6102 Malters	LU	Choana	neg.	n.d.	n.d.	N/A
**594K**	Carrion crow	*Corvus corone*	6102 Malters	LU	Cloaca	neg.	n.d.	n.d.	N/A
**594Kot**	Carrion crow	*Corvus corone*	6102 Malters	LU	Feces	neg.	n.d.	n.d.	N/A
**595Kot**	Tawny owl	*Strix aluco*	St. Gallen	SG	Feces	neg.	n.d.	n.d.	N/A
**596Kot**	Carrion crow	*Corvus corone*	6015 Luzern	LU	Feces	neg.	n.d.	n.d.	N/A
**597Kot**	Tawny owl	*Strix aluco*	4457 Diegten	BL	Feces	neg.	n.d.	n.d.	N/A
**598Kot**	Eurasian magpie	*Pica pica*	8805 Richterswil	ZH	Feces	neg.	n.d.	n.d.	N/A
**599Kot**	Eurasian magpie	*Pica pica*	6000 Luzern	LU	Feces	neg.	n.d.	n.d.	N/A
**600C**	Carrion crow	*Corvus corone*	5737 Menziken	AG	Choana	neg.	n.d.	n.d.	N/A
**600K**	Carrion crow	*Corvus corone*	5737 Menziken	AG	Cloaca	neg.	n.d.	n.d.	N/A
**601K**	Red kite	*Milvus milvus*	6207 Nottwil	LU	Cloaca	neg.	n.d.	n.d.	N/A
**602Kot**	Carrion crow	*Corvus corone*	6102 Malters	LU	Feces	neg.	n.d.	n.d.	N/A
**603C**	Eurasian jay	*Garrulus glandarius*	8134 Adliswil	ZH	Choana	neg.	n.d.	n.d.	N/A
**603K**	Eurasian jay	*Garrulus glandarius*	8134 Adliswil	ZH	Cloaca	neg.	n.d.	n.d.	N/A
**604C**	Carrion crow	*Corvus corone*	Luzern	LU	Cloaca	neg.	n.d.	n.d.	N/A
**604K**	Carrion crow	*Corvus corone*	Luzern	LU	Choana	neg.	n.d.	n.d.	N/A
**604Kot**	Carrion crow	*Corvus corone*	Luzern	LU	Feces	neg.	n.d.	n.d.	N/A
**605C**	Carrion crow	*Corvus corone*	6274 Eschenbach	SG	Choana	neg.	n.d.	n.d.	N/A
**605K**	Carrion crow	*Corvus corone*	6274 Eschenbach	SG	Cloaca	neg.	n.d.	n.d.	N/A
**606Kot**	Common buzzard	*Buteo buteo*	6027 Römerswil	LU	Feces	neg.	n.d.	n.d.	N/A
**607Kot**	Common buzzard	*Buteo buteo*	6027 Römerswil	LU	Feces	neg.	n.d.	n.d.	N/A
**608Kot**	Carrion crow	*Corvus corone*	6383 Dallenwil	NW	Feces	neg.	n.d.	n.d.	N/A
**609Kot**	Carrion crow	*Corvus corone*	6022 Grosswangen	LU	Feces	neg.	n.d.	n.d.	N/A
**611C**	Eurasian magpie	*Pica pica*	6017 Ruswil	LU	Choana	neg.	n.d.	n.d.	N/A
**611K**	Eurasian magpie	*Pica pica*	6017 Ruswil	LU	Cloaca	neg.	n.d.	n.d.	N/A
**611Kot**	Eurasian magpie	*Pica pica*	6017 Ruswil	LU	Feces	neg.	n.d.	n.d.	N/A
**612C**	Carrion crow	*Corvus corone*	6212 St. Erhard	LU	Choana	neg.	n.d.	n.d.	N/A
**612K**	Carrion crow	*Corvus corone*	6212 St. Erhard	LU	Cloaca	neg.	n.d.	n.d.	N/A
**613Kot**	Carrion crow	*Corvus corone*	6280 Urswil	LU	Feces	neg.	n.d.	n.d.	N/A
**614C**	Carrion crow	*Corvus corone*	6242 Wauwil	LU	Choana	neg.	n.d.	n.d.	N/A
**614K**	Carrion crow	*Corvus corone*	6242 Wauwil	LU	Cloaca	neg.	n.d.	n.d.	N/A
**615C**	Common buzzard	*Buteo buteo*	6103 Schwarzenberg	LU	Choana	neg.	n.d.	n.d.	N/A
**615K**	Common buzzard	*Buteo buteo*	6103 Schwarzenberg	LU	Cloaca	neg.	n.d.	n.d.	N/A
**616Kot**	Red kite	*Milvus milvus*	5628 Aristau	AG	Feces	neg.	n.d.	n.d.	N/A
**617C**	Eurasian magpie	*Pica pica*	6015 Luzern	LU	Choana	neg.	n.d.	n.d.	N/A
**617K**	Eurasian magpie	*Pica pica*	6015 Luzern	LU	Cloaca	neg.	n.d.	n.d.	N/A
**617Kot**	Eurasian magpie	*Pica pica*	6015 Luzern	LU	Feces	neg.	n.d.	n.d.	N/A
**618C**	Carrion crow	*Corvus corone*	6287 Aesch	LU	Choana	neg.	n.d.	n.d.	N/A
**618K**	Carrion crow	*Corvus corone*	6287 Aesch	LU	Cloaca	neg.	n.d.	n.d.	N/A
**619C**	Carrion crow	*Corvus corone*	4312 Magden	AG	Choana	neg.	n.d.	n.d.	N/A
**619K**	Carrion crow	*Corvus corone*	4312 Magden	AG	Cloaca	neg.	n.d.	n.d.	N/A

**620Kot**	Common kestrel	*Falco tinnunculus*	6373 Ennetbürgen	NW	Feces	neg.	n.d.	n.d.	N/A
**621C**	Rook	*Corvus frugilegus*	Unknown	N/A	Choana	pos. (23.9)	neg.	neg.	*C. psittaci* 1V
**621K**	Rook	*Corvus frugilegus*	Unknown	N/A	Cloaca	neg.	n.d.	n.d.	N/A
**621Kot**	Rook	*Corvus frugilegus*	Unknown	N/A	Feces	neg.	n.d.	n.d.	N/A
**622C**	Eurasian magpie	*Pica pica*	6102 Malters	LU	Choana	neg.	n.d.	n.d.	N/A
**622K**	Eurasian magpie	*Pica pica*	6102 Malters	LU	Cloaca	neg.	n.d.	n.d.	N/A
**622Kot**	Eurasian magpie	*Pica pica*	6102 Malters	LU	Feces	neg.	n.d.	n.d.	N/A
**623C**	Eurasian magpie	*Pica pica*	5634 Merenschwand	AG	Choana	neg.	n.d.	n.d.	N/A
**623K**	Eurasian magpie	*Pica pica*	5634 Merenschwand	AG	Cloaca	neg.	n.d.	n.d.	N/A
**623Kot**	Eurasian magpie	*Pica pica*	5634 Merenschwand	AG	Feces	neg.	n.d.	n.d.	N/A
**624Kot**	Common kestrel	*Falco tinnunculus*	6014 Luzern	LU	Feces	neg.	n.d.	n.d.	N/A
**625C**	Black kite	*Milvus migrans*	6289 Hämikon	LU	Choana	neg.	n.d.	n.d.	N/A
**625K**	Black kite	*Milvus migrans*	6289 Hämikon	LU	Cloaca	neg.	n.d.	n.d.	N/A
**626C**	Eurasian magpie	*Pica pica*	6203 Sempach-Station	LU	Choana	neg.	n.d.	n.d.	N/A
**626K**	Eurasian magpie	*Pica pica*	6203 Sempach-Station	LU	Cloaca	neg.	n.d.	n.d.	N/A
**626Kot**	Eurasian magpie	*Pica pica*	6203 Sempach-Station	LU	Feces	neg.	n.d.	n.d.	N/A
**627Kot**	Carrion crow	*Corvus corone*	6020 Emmenbrücke	LU	Feces	neg.	n.d.	n.d.	N/A
**628Kot**	Western jackdaw		6000 Luzern	LU	Feces	neg.	n.d.	n.d.	N/A
**629C**	Carrion crow	*Corvus corone*	5628 Aristau	AG	Choana	neg.	n.d.	n.d.	N/A
**629K**	Carrion crow	*Corvus corone*	5628 Aristau	AG	Cloaca	neg.	n.d.	n.d.	N/A
**630C**	Carrion crow	*Corvus corone*	6005 Luzern	LU	Choana	neg.	n.d.	n.d.	N/A
**630K**	Carrion crow	*Corvus corone*	6005 Luzern	LU	Cloaca	neg.	n.d.	n.d.	N/A
**631C**	Western jackdaw	*Corvus monedula*	4915 St. Urban	LU	Choana	neg.	n.d.	n.d.	N/A
**631K**	Western jackdaw	*Corvus monedula*	4915 St. Urban	LU	Cloaca	neg.	n.d.	n.d.	N/A
**631Kot**	Western jackdaw	*Corvus monedula*	4915 St. Urban	LU	Feces	neg.	n.d.	n.d.	N/A
**632Kot**	Western jackdaw	*Corvus monedula*	Unknown	N/A	Feces	neg.	n.d.	n.d.	N/A
**633C**	Carrion crow	*Corvus corone*	6006 Luzern	LU	Choana	neg.	n.d.	n.d.	N/A
**633K**	Carrion crow	*Corvus corone*	6006 Luzern	LU	Cloaca	neg.	n.d.	n.d.	N/A
**635C**	Carrion crow	*Corvus corone*	5037 Muhen	AG	Choana	neg.	n.d.	n.d.	N/A
**635K**	Carrion crow	*Corvus corone*	5037 Muhen	AG	Cloaca	neg.	n.d.	n.d.	N/A
**636C**	Barn owl	*Tyto alba*	6203 Sempach-Station	LU	Choana	neg.	n.d.	n.d.	N/A
**636K**	Barn owl	*Tyto alba*	6203 Sempach-Station	LU	Cloaca	neg.	n.d.	n.d.	N/A
**636Kot**	Barn owl	*Tyto alba*	6203 Sempach-Station	LU	Feces	neg.	n.d.	n.d.	N/A
**637C**	Carrion crow	*Corvus corone*	6000 Luzern	LU	Choana	neg.	n.d.	n.d.	N/A
**637K**	Carrion crow	*Corvus corone*	6000 Luzern	LU	Cloaca	neg.	n.d.	n.d.	N/A
**655C**	Common buzzard	*Buteo buteo*	8213 Neunkirch	SH	Choana	neg.	n.d.	n.d.	N/A
**655K**	Common buzzard	*Buteo buteo*	8213 Neunkirch	SH	Cloaca	neg.	n.d.	n.d.	N/A
**656C**	Eurasian sparrowhawk	*Accipiter nisus*	8625 Gossau	SG	Choana	neg.	n.d.	n.d.	N/A
**656K**	Eurasian sparrowhawk	*Accipiter nisus*	8625 Gossau	SG	Cloaca	neg.	n.d.	n.d.	N/A
**657C**	Common buzzard	*Buteo buteo*	9548 Matzingen	TG	Choana	neg.	n.d.	n.d.	N/A
**657K**	Common buzzard	*Buteo buteo*	9548 Matzingen	TG	Cloaca	neg.	n.d.	n.d.	N/A
**658C**	Common buzzard	*Buteo buteo*	8302 Kloten	ZH	Choana	neg.	n.d.	n.d.	N/A
**658K**	Common buzzard	*Buteo buteo*	8302 Kloten	ZH	Cloaca	neg.	n.d.	n.d.	N/A

**659C**	Red kite	*Milvus milvus*	8547 Kefikon	TG	Choana	neg.	n.d.	n.d.	N/A
**659K**	Red kite	*Milvus milvus*	8547 Kefikon	TG	Cloaca	neg.	n.d.	n.d.	N/A
**660C**	Common buzzard	*Buteo buteo*	8590 Romanshorn	TG	Choana	neg.	n.d.	n.d.	N/A
**660K**	Common buzzard	*Buteo buteo*	8590 Romanshorn	TG	Cloaca	neg.	n.d.	n.d.	N/A
**661C**	Common kestrel	*Falco tinnunculus*	3267 Seedorf	BE	Choana	neg.	n.d.	n.d.	N/A
**661K**	Common kestrel	*Falco tinnunculus*	3267 Seedorf	BE	Cloaca	neg.	n.d.	n.d.	N/A
**662C**	Common kestrel	*Falco tinnunculus*	3125 Toffen	BE	Choana	neg.	n.d.	n.d.	N/A
**662K**	Common kestrel	*Falco tinnunculus*	3125 Toffen	BE	Cloaca	neg.	n.d.	n.d.	N/A
**663C**	Common buzzard	*Buteo buteo*	4553 Subingen	SO	Choana	neg.	n.d.	n.d.	N/A
**663K**	Common buzzard	*Buteo buteo*	4553 Subingen	SO	Cloaca	neg.	n.d.	n.d.	N/A
**664C**	Eurasian magpie	*Pica pica*	3280 Murten	FR	Choana	neg.	n.d.	n.d.	N/A
**664K**	Eurasian magpie	*Pica pica*	3280 Murten	FR	Cloaca	neg.	n.d.	n.d.	N/A
**665C**	Common buzzard	*Buteo buteo*	3628 Uttigen	BE	Choana	neg.	n.d.	n.d.	N/A
**665K**	Common buzzard	*Buteo buteo*	3628 Uttigen	BE	Cloaca	neg.	n.d.	n.d.	N/A
**666Kot**	Common buzzard	*Buteo buteo*	3380 Wangen a.d. Aare	BE	Feces	neg.	n.d.	n.d.	N/A
**667C**	Red kite	*Milvus milvus*	8454 Buchberg	SH	Choana	neg.	n.d.	n.d.	N/A
**667K**	Red kite	*Milvus milvus*	8454 Buchberg	SH	Cloaca	neg.	n.d.	n.d.	N/A
**668C**	Common buzzard	*Buteo buteo*	8416 Flaach	ZH	Choana	pos. (38.3)	neg.	neg.	Not identified
**668K**	Common buzzard	*Buteo buteo*	8416 Flaach	ZH	Cloaca	neg.	n.d.	n.d.	N/A
**669C**	Common buzzard	*Buteo buteo*	Unknown	N/A	Choana	pos. (27.9)	neg.	neg.	*C. psittaci* M56
**669K**	Common buzzard	*Buteo buteo*	Unknown	N/A	Cloaca	pos. (16.2)	neg.	neg.	*C. psittaci* M56
**670C**	Common buzzard	*Buteo buteo*	Unknown	N/A	Choana	neg.	n.d.	n.d.	N/A
**670K**	Common buzzard	*Buteo buteo*	Unknown	N/A	Cloaca	neg.	n.d.	n.d.	N/A
**671C**	Common buzzard	*Buteo buteo*	8254 Basadingen	TG	Choana	pos. (34.0)	neg.	neg.	*C. psittaci* M56
**671K**	Common buzzard	*Buteo buteo*	8254 Basadingen	TG	Cloaca	pos. (27.1)	neg.	neg.	*C. psittaci* M56
**672C**	Common buzzard	*Buteo buteo*	8315 Tagelswangen	ZH	Choana	pos. (37.1)	neg.	neg.	Not identified
**672K**	Common buzzard	*Buteo buteo*	8315 Tagelswangen	ZH	Cloaca	pos. (37.5)	neg.	neg.	Not identified
**673C**	Common buzzard	*Buteo buteo*	8200 Schaffhausen	SH	Choana	pos. (35.8)	neg.	neg.	Not identified
**673K**	Common buzzard	*Buteo buteo*	8200 Schaffhausen	SH	Cloaca	pos. (35.9)	neg.	neg.	Not identified
**674C**	Eurasian sparrowhawk	*Accipiter nisus*	Unknown	N/A	Choana	pos. (36.9)	neg.	neg.	Not identified
**674K**	Eurasian sparrowhawk	*Accipiter nisus*	Unknown	N/A	Cloaca	pos. (36.67)	neg.	neg.	Not identified
**675C**	Eurasian sparrowhawk	*Accipiter nisus*	8472 Seuzach	ZH	Choana	neg.	n.d.	n.d.	N/A
**675K**	Eurasian sparrowhawk	*Accipiter nisus*	8472 Seuzach	ZH	Cloaca	pos. (36.62)	neg.	neg.	Not identified
**676C**	Eurasian sparrowhawk	*Accipiter nisus*	8192 Glattfelden	ZH	Choana	neg.	n.d.	n.d.	N/A
**676K**	Eurasian sparrowhawk	*Accipiter nisus*	8192 Glattfelden	ZH	Cloaca	neg.	n.d.	n.d.	N/A
**677C**	Red kite	*Milvus milvus*	8952 Schlieren	ZH	Choana	neg.	n.d.	n.d.	N/A
**677K**	Red kite	*Milvus milvus*	8952 Schlieren	ZH	Cloaca	pos. (33.5)	neg.	neg.	Not identified
**679C**	Common buzzard	*Buteo buteo*	9113 Degersheim	SG	Choana	neg.	n.d.	n.d.	N/A
**679K**	Common buzzard	*Buteo buteo*	9113 Degersheim	SG	Cloaca	neg.	n.d.	n.d.	N/A
**680C**	Common buzzard	*Buteo buteo*	5620 Bremgarten	AG	Choana	neg.	n.d.	n.d.	N/A
**680K**	Common buzzard	*Buteo buteo*	5620 Bremgarten	AG	Cloaca	neg.	n.d.	n.d.	N/A
**681C**	Common buzzard	*Buteo buteo*	8232 Merishausen	SH	Choana	neg.	n.d.	n.d.	N/A

**681K**	Common buzzard	*Buteo buteo*	8232 Merishausen	SH	Cloaca	neg.	n.d.	n.d.	N/A
**682C**	Common buzzard	*Buteo buteo*	Unknown	N/A	Choana	neg.	n.d.	n.d.	N/A
**682K**	Common buzzard	*Buteo buteo*	Unknown	N/A	Cloaca	neg.	n.d.	n.d.	N/A
**683C**	Common buzzard	*Buteo buteo*	Unknown	N/A	Choana	pos. (33.3)	pos. (33.2)	neg.	*C. psittaci* A
**683K**	Common buzzard	*Buteo buteo*	Unknown	N/A	Cloaca	neg.	n.d.	n.d.	N/A
**684C**	Carrion crow	*Corvus corone*	Stadt Zürich	ZH	Choana	neg.	n.d.	n.d.	N/A
**684K**	Carrion crow	*Corvus corone*	Stadt Zürich	ZH	Cloaca	neg.	n.d.	n.d.	N/A
**685C**	Carrion crow	*Corvus corone*	Stadt Zürich	ZH	Choana	neg.	n.d.	n.d.	N/A
**685K**	Carrion crow	*Corvus corone*	Stadt Zürich	ZH	Cloaca	neg.	n.d.	n.d.	N/A
**686C**	Carrion crow	*Corvus corone*	8003 Zürich	ZH	Choana	pos. (34.5)	neg.	neg.	*C. abortus*/*C. psittaci*
**686K**	Carrion crow	*Corvus corone*	8003 Zürich	ZH	Cloaca	neg.	n.d.	n.d.	N/A
**687C**	Common buzzard	*Buteo buteo*	8465 Trüllikon	ZH	Choana	neg.	n.d.	n.d.	N/A
**687K**	Common buzzard	*Buteo buteo*	8465 Trüllikon	ZH	Cloaca	neg.	n.d.	n.d.	N/A
**688C**	Carrion crow	*Corvus corone*	Stadt Zürich	ZH	Choana	pos. (28.2)	neg.	neg.	*C. psittaci* 1V
**688K**	Carrion crow	*Corvus corone*	Stadt Zürich	ZH	Cloaca	pos. (33.8)	neg.	neg.	*C. abortus*/*C. psittaci*
**689C**	Carrion crow	*Corvus corone*	Stadt Zürich	ZH	Choana	pos. (26.8)	neg.	neg.	*C. psittaci* 1V
**689K**	Carrion crow	*Corvus corone*	Stadt Zürich	ZH	Cloaca	pos. (33.2)	neg.	neg.	*C. abortus*/*C. psittaci*
**690C**	Carrion crow	*Corvus corone*	Stadt Zürich	ZH	Choana	pos. (29.4)	neg.	neg.	*C. psittaci* 1V
**690K**	Carrion crow	*Corvus corone*	Stadt Zürich	ZH	Cloaca	neg.	n.d.	n.d.	N/A
**691C**	Carrion crow	*Corvus corone*	Stadt Zürich	ZH	Choana	neg.	n.d.	n.d.	N/A
**691K**	Carrion crow	*Corvus corone*	Stadt Zürich	ZH	Cloaca	neg.	n.d.	n.d.	N/A
**692C**	Carrion crow	*Corvus corone*	Stadt Zürich	ZH	Choana	neg.	n.d.	n.d.	N/A
**692K**	Carrion crow	*Corvus corone*	Stadt Zürich	ZH	Cloaca	neg.	n.d.	n.d.	N/A
**693C**	Carrion crow	*Corvus corone*	Stadt Zürich	ZH	Choana	neg.	n.d.	n.d.	N/A
**693K**	Carrion crow	*Corvus corone*	Stadt Zürich	ZH	Cloaca	neg.	n.d.	n.d.	N/A
**694C**	Carrion crow	*Corvus corone*	Stadt Zürich	ZH	Choana	neg.	n.d.	n.d.	N/A
**694K**	Carrion crow	*Corvus corone*	Stadt Zürich	ZH	Cloaca	neg.	n.d.	n.d.	N/A
**695C**	Carrion crow	*Corvus corone*	Stadt Zürich	ZH	Choana	neg.	n.d.	n.d.	N/A
**695K**	Carrion crow	*Corvus corone*	Stadt Zürich	ZH	Cloaca	neg.	n.d.	n.d.	N/A
**696C**	Carrion crow	*Corvus corone*	Stadt Zürich	ZH	Choana	pos. (28.9)	neg.	neg.	*C. abortus*/*C. psittaci*
**696K**	Carrion crow	*Corvus corone*	Stadt Zürich	ZH	Cloaca	pos. (36.9 (1:10))	neg.	neg.	Not identified
**697C**	Carrion crow	*Corvus corone*	Stadt Zürich	ZH	Choana	neg.	n.d.	n.d.	N/A
**697K**	Carrion crow	*Corvus corone*	Stadt Zürich	ZH	Cloaca	neg.	n.d.	n.d.	N/A
**698C**	Carrion crow	*Corvus corone*	Stadt Zürich	ZH	Choana	neg.	n.d.	n.d.	N/A
**698K**	Carrion crow	*Corvus corone*	Stadt Zürich	ZH	Cloaca	neg.	n.d.	n.d.	N/A
**699C**	Carrion crow	*Corvus corone*	Stadt Zürich	ZH	Choana	neg.	n.d.	n.d.	N/A
**699K**	Carrion crow	*Corvus corone*	Stadt Zürich	ZH	Cloaca	neg.	n.d.	n.d.	N/A
**700C**	Carrion crow	*Corvus corone*	Stadt Zürich	ZH	Choana	neg.	neg.	neg.	N/A
**700K**	Carrion crow	*Corvus corone*	Stadt Zürich	ZH	Cloaca	neg.	n.d.	n.d.	N/A
**701C**	Carrion crow	*Corvus corone*	Stadt Zürich	ZH	Choana	neg.	n.d.	n.d.	N/A
**701K**	Carrion crow	*Corvus corone*	Stadt Zürich	ZH	Cloaca	neg.	n.d.	n.d.	N/A
**702C**	Carrion crow	*Corvus corone*	Stadt Zürich	ZH	Choana	pos. (31.6)	neg.	neg.	*C. abortus*/*C. psittaci*
**702K**	Carrion crow	*Corvus corone*	Stadt Zürich	ZH	Cloaca	neg.	n.d.	n.d.	N/A

**703C**	Carrion crow	*Corvus corone*	Stadt Zürich	ZH	Choana	neg.	n.d.	n.d.	N/A
**703K**	Carrion crow	*Corvus corone*	Stadt Zürich	ZH	Cloaca	neg.	n.d.	n.d.	N/A
**704C**	Carrion crow	*Corvus corone*	Stadt Zürich	ZH	Choana	neg.	n.d.	n.d.	N/A
**704K**	Carrion crow	*Corvus corone*	Stadt Zürich	ZH	Cloaca	neg.	n.d.	n.d.	N/A
**705C**	Carrion crow	*Corvus corone*	Stadt Zürich	ZH	Choana	pos. (30.9)	neg.	neg.	*C. abortus*/*C. psittaci*
**705K**	Carrion crow	*Corvus corone*	Stadt Zürich	ZH	Cloaca	pos. (37.2)	neg.	neg.	Not identified
**706C**	Carrion crow	*Corvus corone*	Stadt Zürich	ZH	Choana	pos. (30.2)	neg.	neg.	*C. psittaci* D
**706K**	Carrion crow	*Corvus corone*	Stadt Zürich	ZH	Cloaca	neg.	n.d.	n.d.	N/A
**707C**	Eurasian magpie	*Pica pica*	Unknown	N/A	Choana	neg.	n.d.	n.d.	N/A
**707K**	Eurasian magpie	*Pica pica*	Unknown	N/A	Cloaca	neg.	n.d.	n.d.	N/A
**708C**	Carrion crow	*Corvus corone*	Stadt Zürich	ZH	Choana	neg.	n.d.	n.d.	N/A
**708K**	Carrion crow	*Corvus corone*	Stadt Zürich	ZH	Cloaca	neg.	n.d.	n.d.	N/A
**711C**	Carrion crow	*Corvus corone*	2564 Bellmund	BE	Choana	pos. (31.6)	neg.	neg.	*C. psittaci* 1V
**711K**	Carrion crow	*Corvus corone*	2564 Bellmund	BE	Cloaca	neg.	n.d.	n.d.	N/A
**712C**	Carrion crow	*Corvus corone*	2564 Bellmund	BE	Choana	neg.	n.d.	n.d.	N/A
**712K**	Carrion crow	*Corvus corone*	2564 Bellmund	BE	Cloaca	neg.	n.d.	n.d.	N/A
**713C**	Carrion crow	*Corvus corone*	2564 Bellmund	BE	Choana	neg.	n.d.	n.d.	N/A
**713K**	Carrion crow	*Corvus corone*	2564 Bellmund	BE	Cloaca	neg.	n.d.	n.d.	N/A
**714C**	Carrion crow	*Corvus corone*	2564 Bellmund	BE	Choana	neg.	n.d.	n.d.	N/A
**714K**	Carrion crow	*Corvus corone*	2564 Bellmund	BE	Cloaca	neg.	n.d.	n.d.	N/A
**715C**	Carrion crow	*Corvus corone*	2564 Bellmund	BE	Choana	pos. (38.1)	neg.	neg.	Not identified
**715K**	Carrion crow	*Corvus corone*	2564 Bellmund	BE	Cloaca	neg.	n.d.	n.d.	N/A
**716C**	Carrion crow	*Corvus corone*	2564 Bellmund	BE	Choana	neg.	n.d.	n.d.	N/A
**716K**	Carrion crow	*Corvus corone*	2564 Bellmund	BE	Cloaca	pos. (30.6)	neg.	neg.	*C. psittaci* D
**717C**	Carrion crow	*Corvus corone*	2564 Bellmund	BE	Choana	neg.	n.d.	n.d.	N/A
**717K**	Carrion crow	*Corvus corone*	2564 Bellmund	BE	Cloaca	neg.	n.d.	n.d.	N/A
**718C**	Rook	*Corvus frugilegus*	2564 Bellmund	BE	Choana	neg.	n.d.	n.d.	N/A
**718K**	Rook	*Corvus frugilegus*	2564 Bellmund	BE	Cloaca	neg.	n.d.	n.d.	N/A
**719C**	Common buzzard	*Buteo buteo*	3270 Aarberg	BE	Choana	neg.	n.d.	n.d.	N/A
**719K**	Common buzzard	*Buteo buteo*	3270 Aarberg	BE	Cloaca	neg.	n.d.	n.d.	N/A
**720C**	Eurasian hobby	*Falco subbuteo*	8452 Adlikon	ZH	Choana	neg.	n.d.	n.d.	N/A
**720K**	Eurasian hobby	*Falco subbuteo*	8452 Adlikon	ZH	Cloaca	neg.	n.d.	n.d.	N/A
**721C**	Carrion crow	*Corvus corone*	Stadt Zürich	ZH	Choana	pos. (29.9)	neg.	neg.	*C. psittaci* 1V
**721K**	Carrion crow	*Corvus corone*	Stadt Zürich	ZH	Cloaca	neg.	n.d.	n.d.	N/A
**722C**	Carrion crow	*Corvus corone*	Stadt Zürich	ZH	Choana	neg.	n.d.	n.d.	N/A
**722K**	Carrion crow	*Corvus corone*	Stadt Zürich	ZH	Cloaca	neg.	n.d.	n.d.	N/A
**723C**	Carrion crow	*Corvus corone*	Stadt Zürich	ZH	Choana	neg.	n.d.	n.d.	N/A
**723K**	Carrion crow	*Corvus corone*	Stadt Zürich	ZH	Cloaca	neg.	n.d.	n.d.	N/A
**724C**	Carrion crow	*Corvus corone*	Stadt Zürich	ZH	Choana	neg.	n.d.	n.d.	N/A
**724K**	Carrion crow	*Corvus corone*	Stadt Zürich	ZH	Cloaca	neg.	n.d.	n.d.	N/A
**725C**	Carrion crow	*Corvus corone*	Stadt Zürich	ZH	Choana	pos. (31.6)	neg.	neg.	*C. abortus*/*C. psittaci*
**725K**	Carrion crow	*Corvus corone*	Stadt Zürich	ZH	Cloaca	neg.	n.d.	n.d.	N/A
**726K**	Common kestrel	*Falco tinnunculus*	Stadt Zürich	ZH	Cloaca	neg.	n.d.	n.d.	N/A

**727C**	Common buzzard	*Buteo buteo*	Unknown	N/A	Choana	neg.	n.d.	n.d.	N/A
**727K**	Common buzzard	*Buteo buteo*	Unknown	N/A	Cloaca	neg.	n.d.	n.d.	N/A
**728C**	Barn owl	*Tyto alba*	1786 Sugiez	FR	Choana	neg.	n.d.	n.d.	N/A
**728K**	Barn owl	*Tyto alba*	1786 Sugiez	FR	Cloaca	neg.	n.d.	n.d.	N/A
**729C**	Tawny owl	*Strix aluco*	1786 Sugiez	FR	Choana	neg.	n.d.	n.d.	N/A
**729K**	Tawny owl	*Strix aluco*	1786 Sugiez	FR	Cloaca	neg.	n.d.	n.d.	N/A
**730C**	Common kestrel	*Falco tinnunculus*	Unknown	N/A	Choana	neg.	n.d.	n.d.	N/A
**730K**	Common kestrel	*Falco tinnunculus*	Unknown	N/A	Cloaca	neg.	n.d.	n.d.	N/A
**731C**	Red kite	*Milvus milvus*	Unknown	N/A	Choana	neg.	n.d.	n.d.	N/A
**731K**	Red kite	*Milvus milvus*	Unknown	N/A	Cloaca	neg.	n.d.	n.d.	N/A
**732C**	Common buzzard	*Buteo buteo*	Unknown	N/A	Choana	neg.	n.d.	n.d.	N/A
**732K**	Common buzzard	*Buteo buteo*	Unknown	N/A	Cloaca	neg.	n.d.	n.d.	N/A
**733C**	Eurasian sparrowhawk	*Accipiter nisus*	Unknown	N/A	Choana	neg.	n.d.	n.d.	N/A
**733K**	Eurasian sparrowhawk	*Accipiter nisus*	Unknown	N/A	Cloaca	neg.	n.d.	n.d.	N/A
**734C**	Long-eared owl	*Asio otus*	Unknown	N/A	Choana	neg.	n.d.	n.d.	N/A
**734K**	Long-eared owl	*Asio otus*	Unknown	N/A	Cloaca	neg.	n.d.	n.d.	N/A
**735C**	Carrion crow	*Corvus corone*	Zug	ZG	Choana	pos. (28.4)	neg.	neg.	*C. psittaci* 1V
**735K**	Carrion crow	*Corvus corone*	Zug	ZG	Cloaca	neg.	n.d.	n.d.	N/A
**736C**	Carrion crow	*Corvus corone*	Zug	ZG	Choana	pos. (28.7)	neg.	neg.	*C. psittaci* 6N
**736K**	Carrion crow	*Corvus corone*	Zug	ZG	Cloaca	pos. (30.6)	neg.	neg.	*C. abortus*/*C. psittaci*
**737C**	Carrion crow	*Corvus corone*	Zug	ZG	Choana	pos. (31.1)	neg.	neg.	*C. abortus*/*C. psittaci*
**737K**	Carrion crow	*Corvus corone*	Zug	ZG	Cloaca	pos. (32.7)	neg.	neg.	*C. abortus*/*C. psittaci*
**738C**	Carrion crow	*Corvus corone*	Zug	ZG	Choana	neg.	n.d.	n.d.	N/A
**738K**	Carrion crow	*Corvus corone*	Zug	ZG	Cloaca	neg.	n.d.	n.d.	N/A
**739C**	Carrion crow	*Corvus corone*	Zug	ZG	Choana	neg.	n.d.	n.d.	N/A
**739K**	Carrion crow	*Corvus corone*	Zug	ZG	Cloaca	neg.	n.d.	n.d.	N/A
**740C**	Carrion crow	*Corvus corone*	Zug	ZG	Choana	pos. (27.7)	neg.	neg.	*C. psittaci* 1V
**740K**	Carrion crow	*Corvus corone*	Zug	ZG	Cloaca	pos. (28.0)	neg.	neg.	*C. abortus*/*C. psittaci*
**741C**	Carrion crow	*Corvus corone*	Zug	ZG	Choana	neg.	n.d.	n.d.	N/A
**741K**	Carrion crow	*Corvus corone*	Zug	ZG	Cloaca	neg.	n.d.	n.d.	N/A
**742C**	Carrion crow	*Corvus corone*	Zug	ZG	Choana	neg.	n.d.	n.d.	N/A
**742K**	Carrion crow	*Corvus corone*	Zug	ZG	Cloaca	neg.	n.d.	n.d.	N/A
**743C**	Carrion crow	*Corvus corone*	Zug	ZG	Choana	neg.	n.d.	n.d.	N/A
**743K**	Carrion crow	*Corvus corone*	Zug	ZG	Cloaca	neg.	n.d.	n.d.	N/A
**744C**	Carrion crow	*Corvus corone*	Zug	ZG	Choana	pos.(33.6)	neg.	neg.	*C. abortus*/*C. psittaci*
**744K**	Carrion crow	*Corvus corone*	Zug	ZG	Cloaca	neg.	n.d.	n.d.	N/A
**745C**	Carrion crow	*Corvus corone*	Zug	ZG	Choana	neg.	n.d.	n.d.	N/A
**745K**	Carrion crow	*Corvus corone*	Zug	ZG	Cloaca	neg.	n.d.	n.d.	N/A
**746C**	Carrion crow	*Corvus corone*	Zug	ZG	Choana	pos. (24.9)	neg.	neg.	*C. psittaci* 1V
**746K**	Carrion crow	*Corvus corone*	Zug	ZG	Cloaca	pos. (39.6)	neg.	neg.	Not identified
**747C**	Carrion crow	*Corvus corone*	Zug	ZG	Choana	neg.	n.d.	n.d.	N/A
**747K**	Carrion crow	*Corvus corone*	Zug	ZG	Cloaca	neg.	n.d.	n.d.	N/A
**748C**	Carrion crow	*Corvus corone*	Zug	ZG	Choana	neg.	n.d.	n.d.	N/A

**748K**	Carrion crow	*Corvus corone*	Zug	ZG	Cloaca	neg.	n.d.	n.d.	N/A
**749C**	Carrion crow	*Corvus corone*	Zug	ZG	Choana	pos. (39.6)	neg.	neg.	Not identified
**749K**	Carrion crow	*Corvus corone*	Zug	ZG	Cloaca	neg.	n.d.	n.d.	N/A
**750C**	Carrion crow	*Corvus corone*	Zug	ZG	Choana	pos. (31.5)	neg.	neg.	*C. abortus*/*C. psittaci*
**750K**	Carrion crow	*Corvus corone*	Zug	ZG	Cloaca	pos. (31.5)	neg.	neg.	*C. abortus*/*C. psittaci*
**751C**	Carrion crow	*Corvus corone*	Zug	ZG	Choana	pos. (20.2)	neg.	neg.	*C. psittaci* 1V
**751K**	Carrion crow	*Corvus corone*	Zug	ZG	Cloaca	pos. (27.7)	neg.	neg.	*C. abortus*/*C. psittaci*
**752C**	Carrion crow	*Corvus corone*	Zug	ZG	Choana	pos. (30.4)	neg.	neg.	*C. psittaci* 1V
**752K**	Carrion crow	*Corvus corone*	Zug	ZG	Cloaca	pos. (33.0)	neg.	neg.	*C. abortus*/*C. psittaci*
**753C**	Carrion crow	*Corvus corone*	Zug	ZG	Choana	pos. (34.0)	neg.	neg.	*C. abortus*/*C. psittaci*
**753K**	Carrion crow	*Corvus corone*	Zug	ZG	Cloaca	neg.	n.d.	n.d.	N/A
**754C**	Carrion crow	*Corvus corone*	Zug	ZG	Choana	pos. (26.3)	neg.	neg.	*C. abortus*/*C. psittaci*
**754K**	Carrion crow	*Corvus corone*	Zug	ZG	Cloaca	pos. (29.3)	neg.	neg.	Not identified
**755C**	Carrion crow	*Corvus corone*	Zug	ZG	Choana	pos. (36.7)	neg.	neg.	Not identified
**755K**	Carrion crow	*Corvus corone*	Zug	ZG	Cloaca	pos. (37.5)	neg.	neg.	Not identified
**756C**	Carrion crow	*Corvus corone*	Zug	ZG	Choana	pos. (34.0)	neg.	neg.	*C. abortus*/*C. psittaci*
**756K**	Carrion crow	*Corvus corone*	Zug	ZG	Cloaca	pos. (32.9)	neg.	neg.	*C. abortus*/*C. psittaci*
**757C**	Carrion crow	*Corvus corone*	Zug	ZG	Choana	pos. (37.28)	neg.	neg.	Not identified
**757K**	Carrion crow	*Corvus corone*	Zug	ZG	Cloaca	neg.	n.d.	n.d.	N/A
**758C**	Carrion crow	*Corvus corone*	Zug	ZG	Choana	neg.	n.d.	n.d.	N/A
**758K**	Carrion crow	*Corvus corone*	Zug	ZG	Cloaca	pos. (39.7)	neg.	neg.	Not identified
**759C**	Carrion crow	*Corvus corone*	Zug	ZG	Choana	pos. (33.1)	neg.	neg.	*C. abortus*/*C. psittaci*
**759K**	Carrion crow	*Corvus corone*	Zug	ZG	Cloaca	neg.	n.d.	n.d.	N/A
**760C**	Carrion crow	*Corvus corone*	Zug	ZG	Choana	pos. (25.9)	neg.	neg.	*C. abortus*/*C. psittaci*
**760K**	Carrion crow	*Corvus corone*	Zug	ZG	Cloaca	pos. (31.9)	neg.	neg.	*C. abortus*/*C. psittaci*
**761C**	Carrion crow	*Corvus corone*	Zug	ZG	Choana	pos. (29.7)	neg.	neg.	*C. psittaci* 1V
**761K**	Carrion crow	*Corvus corone*	Zug	ZG	Cloaca	neg.	neg.	neg.	N/A
**762C**	Carrion crow	*Corvus corone*	Zug	ZG	Choana	pos. (35.8)	neg.	neg.	Not identified
**762K**	Carrion crow	*Corvus corone*	Zug	ZG	Cloaca	neg.	n.d.	n.d.	N/A
**763C**	Carrion crow	*Corvus corone*	Zug	ZG	Choana	neg.	n.d.	n.d.	N/A
**763K**	Carrion crow	*Corvus corone*	Zug	ZG	Cloaca	neg.	n.d.	n.d.	N/A
**764C**	Carrion crow	*Corvus corone*	Zug	ZG	Choana	pos. (33.9)	neg.	neg.	*C. abortus*/*C. psittaci*
**764K**	Carrion crow	*Corvus corone*	Zug	ZG	Cloaca	neg.	n.d.	n.d.	N/A
**765C**	Carrion crow	*Corvus corone*	Zug	ZG	Choana	pos. (33.6)	neg.	neg.	*C. abortus*/*C. psittaci*
**765K**	Carrion crow	*Corvus corone*	Zug	ZG	Cloaca	neg.	n.d.	n.d.	N/A
**766C**	Carrion crow	*Corvus corone*	Zug	ZG	Choana	neg.	n.d.	n.d.	N/A
**766K**	Carrion crow	*Corvus corone*	Zug	ZG	Cloaca	neg.	n.d.	n.d.	N/A
**767C**	Carrion crow	*Corvus corone*	Zug	ZG	Choana	neg.	n.d.	n.d.	N/A
**767K**	Carrion crow	*Corvus corone*	Zug	ZG	Cloaca	neg.	n.d.	n.d.	N/A
**768C**	Carrion crow	*Corvus corone*	Zug	ZG	Choana	neg.	n.d.	n.d.	N/A
**768K**	Carrion crow	*Corvus corone*	Zug	ZG	Cloaca	neg.	n.d.	n.d.	N/A
**769C**	Carrion crow	*Corvus corone*	Zug	ZG	Choana	pos. (24.6)	neg.	neg.	*C. abortus*/*C. psittaci*
**769K**	Carrion crow	*Corvus corone*	Zug	ZG	Cloaca	neg.	n.d.	n.d.	N/A

**770C**	Carrion crow	*Corvus corone*	Zug	ZG	Choana	pos. (31.2)	neg.	neg.	*C. abortus*/*C. psittaci*
**770K**	Carrion crow	*Corvus corone*	Zug	ZG	Cloaca	neg.	n.d.	n.d.	N/A
**771C**	Carrion crow	*Corvus corone*	Zug	ZG	Choana	neg.	n.d.	n.d.	N/A
**771K**	Carrion crow	*Corvus corone*	Zug	ZG	Cloaca	neg.	n.d.	n.d.	N/A
**772C**	Carrion crow	*Corvus corone*	Zug	ZG	Choana	pos. (29.9)	neg.	neg.	*C. abortus*/*C. psittaci*
**772K**	Carrion crow	*Corvus corone*	Zug	ZG	Cloaca	pos. (26.8)	neg.	neg.	*C. psittaci* 1V
**773C**	Carrion crow	*Corvus corone*	Zug	ZG	Choana	pos. (31.0)	neg.	neg.	*C. abortus*/*C. psittaci*
**773K**	Carrion crow	*Corvus corone*	Zug	ZG	Cloaca	pos. (37.4 (1:10))	neg.	neg.	Not identified
**774C**	Carrion crow	*Corvus corone*	Zug	ZG	Choana	pos. (31.9)	neg.	neg.	*C. abortus*/*C. psittaci*
**774K**	Carrion crow	*Corvus corone*	Zug	ZG	Cloaca	neg.	n.d.	n.d.	N/A
**775C**	Carrion crow	*Corvus corone*	Zug	ZG	Choana	neg.	n.d.	n.d.	N/A
**775K**	Carrion crow	*Corvus corone*	Zug	ZG	Cloaca	neg.	n.d.	n.d.	N/A
**776C**	Carrion crow	*Corvus corone*	Zug	ZG	Choana	neg.	n.d.	n.d.	N/A
**776K**	Carrion crow	*Corvus corone*	Zug	ZG	Cloaca	neg.	n.d.	n.d.	N/A
**777C**	Carrion crow	*Corvus corone*	Zug	ZG	Choana	neg.	n.d.	n.d.	N/A
**777K**	Carrion crow	*Corvus corone*	Zug	ZG	Cloaca	neg.	n.d.	n.d.	N/A
**778C**	Carrion crow	*Corvus corone*	Zug	ZG	Choana	neg.	n.d.	n.d.	N/A
**778K**	Carrion crow	*Corvus corone*	Zug	ZG	Cloaca	neg.	n.d.	n.d.	N/A
**779C**	Carrion crow	*Corvus corone*	Zug	ZG	Choana	neg.	n.d.	n.d.	N/A
**779K**	Carrion crow	*Corvus corone*	Zug	ZG	Cloaca	neg.	n.d.	n.d.	N/A
**780C**	Common buzzard	*Buteo buteo*	Zug	ZG	Choana	neg.	n.d.	n.d.	N/A
**780K**	Common buzzard	*Buteo buteo*	Zug	ZG	Cloaca	neg.	n.d.	n.d.	N/A
**781C**	Common buzzard	*Buteo buteo*	Zug	ZG	Choana	neg.	neg.	neg.	N/A
**782C**	Common buzzard	*Buteo buteo*	Zug	ZG	Choana	neg.	n.d.	n.d.	N/A
**782K**	Common buzzard	*Buteo buteo*	Zug	ZG	Cloaca	neg.	n.d.	n.d.	N/A
**783C**	Common buzzard	*Buteo buteo*	Zug	ZG	Choana	neg.	n.d.	n.d.	N/A
**783K**	Common buzzard	*Buteo buteo*	Zug	ZG	Cloaca	neg.	n.d.	n.d.	N/A
**784C**	Common kestrel	*Falco tinnunculus*	8610 Uster	ZH	Choana	neg.	n.d.	n.d.	N/A
**784K**	Common kestrel	*Falco tinnunculus*	8610 Uster	ZH	Cloaca	neg.	n.d.	n.d.	N/A
**785C**	Red kite	*Milvus milvus*	Schaffhausen	SH	Choana	neg.	n.d.	n.d.	N/A
**785K**	Red kite	*Milvus milvus*	Schaffhausen	SH	Cloaca	neg.	n.d.	n.d.	N/A
**796C**	Long-eared owl	*Asio otus*	Zürich Flughafen	ZH	Choana	neg.	n.d.	n.d.	N/A
**796K**	Long-eared owl	*Asio otus*	Zürich Flughafen	ZH	Cloaca	neg.	n.d.	n.d.	N/A
**797C**	Carrion crow	*Corvus corone*	Zürich Flughafen	ZH	Choana	pos. (33.5)	neg.	neg.	*C. abortus*/*C. psittaci*
**797K**	Carrion crow	*Corvus corone*	Zürich Flughafen	ZH	Cloaca	pos. (33.6)	neg.	neg.	*C. abortus*/*C. psittaci*
**798C**	Carrion crow	*Corvus corone*	Zürich Flughafen	ZH	Choana	pos. (28.2)	neg.	neg.	*C. abortus*/*C. psittaci*
**798K**	Carrion crow	*Corvus corone*	Zürich Flughafen	ZH	Cloaca	pos. (26.7)	neg.	neg.	*C. psittaci* 1V
**799C**	Carrion crow	*Corvus corone*	Zürich Flughafen	ZH	Choana	neg.	n.d.	n.d.	N/A
**799K**	Carrion crow	*Corvus corone*	Zürich Flughafen	ZH	Cloaca	neg.	n.d.	n.d.	N/A
**800C**	Carrion crow	*Corvus corone*	Zürich Flughafen	ZH	Choana	neg.	n.d.	n.d.	N/A
**800K**	Carrion crow	*Corvus corone*	Zürich Flughafen	ZH	Cloaca	neg.	n.d.	n.d.	N/A
**801C**	Carrion crow	*Corvus corone*	Zürich Flughafen	ZH	Choana	neg.	n.d.	n.d.	N/A
**801K**	Carrion crow	*Corvus corone*	Zürich Flughafen	ZH	Cloaca	neg.	n.d.	n.d.	N/A

**802C**	Long-eared owl	*Asio otus*	Zürich Flughafen	ZH	Choana	neg.	n.d.	n.d.	N/A
**802K**	Long-eared owl	*Asio otus*	Zürich Flughafen	ZH	Cloaca	neg.	n.d.	n.d.	N/A
**803C**	Carrion crow	*Corvus corone*	Zürich Flughafen	ZH	Choana	neg.	n.d.	n.d.	N/A
**803K**	Carrion crow	*Corvus corone*	Zürich Flughafen	ZH	Cloaca	neg.	n.d.	n.d.	N/A
**804C**	Carrion crow	*Corvus corone*	Zürich Flughafen	ZH	Choana	neg.	n.d.	n.d.	N/A
**804K**	Carrion crow	*Corvus corone*	Zürich Flughafen	ZH	Cloaca	neg.	n.d.	n.d.	N/A
**805C**	Carrion crow	*Corvus corone*	Zürich Flughafen	ZH	Choana	neg.	n.d.	n.d.	N/A
**805K**	Carrion crow	*Corvus corone*	Zürich Flughafen	ZH	Cloaca	neg.	n.d.	n.d.	N/A
**806C**	Carrion crow	*Corvus corone*	Zürich Flughafen	ZH	Choana	neg.	n.d.	n.d.	N/A
**806K**	Carrion crow	*Corvus corone*	Zürich Flughafen	ZH	Cloaca	neg.	n.d.	n.d.	N/A
**807C**	Carrion crow	*Corvus corone*	Zürich Flughafen	ZH	Choana	neg.	n.d.	n.d.	N/A
**807K**	Carrion crow	*Corvus corone*	Zürich Flughafen	ZH	Cloaca	neg.	n.d.	n.d.	N/A
**808C**	Eurasian magpie	*Pica pica*	Zürich Flughafen	ZH	Choana	neg.	n.d.	n.d.	N/A
**808K**	Eurasian magpie	*Pica pica*	Zürich Flughafen	ZH	Cloaca	neg.	n.d.	n.d.	N/A
**809C**	Carrion crow	*Corvus corone*	Zürich Flughafen	ZH	Choana	neg.	n.d.	n.d.	N/A
**809K**	Carrion crow	*Corvus corone*	Zürich Flughafen	ZH	Cloaca	neg.	n.d.	n.d.	N/A
**810C**	Carrion crow	*Corvus corone*	Zürich Flughafen	ZH	Choana	neg.	n.d.	n.d.	N/A
**810K**	Carrion crow	*Corvus corone*	Zürich Flughafen	ZH	Cloaca	neg.	n.d.	n.d.	N/A
**811C**	Carrion crow	*Corvus corone*	Zürich Flughafen	ZH	Choana	neg.	n.d.	n.d.	N/A
**811K**	Carrion crow	*Corvus corone*	Zürich Flughafen	ZH	Cloaca	neg.	n.d.	n.d.	N/A
**812C**	Carrion crow	*Corvus corone*	Zürich Flughafen	ZH	Choana	neg.	n.d.	n.d.	N/A
**812K**	Carrion crow	*Corvus corone*	Zürich Flughafen	ZH	Cloaca	neg.	n.d.	n.d.	N/A
**813C**	Carrion crow	*Corvus corone*	Zürich Flughafen	ZH	Choana	neg.	n.d.	n.d.	N/A
**813K**	Carrion crow	*Corvus corone*	Zürich Flughafen	ZH	Cloaca	neg.	n.d.	n.d.	N/A
**814C**	Carrion crow	*Corvus corone*	Zürich Flughafen	ZH	Choana	pos. (25.6)	neg.	neg.	*C. psittaci* 1V
**814K**	Carrion crow	*Corvus corone*	Zürich Flughafen	ZH	Cloaca	pos. (29.1)	neg.	neg.	*C. abortus*/*C. psittaci*
**815C**	Carrion crow	*Corvus corone*	Zürich Flughafen	ZH	Choana	neg.	n.d.	n.d.	N/A
**815K**	Carrion crow	*Corvus corone*	Zürich Flughafen	ZH	Cloaca	neg.	n.d.	n.d.	N/A
**816Kot**	Common kestrel	*Falco tinnunculus*	3256 Bangerten	BE	Feces	neg.	n.d.	n.d.	N/A
**817Kot**	Tawny owl	*Strix aluco*	3253 Schnottwil	SO	Feces	neg.	n.d.	n.d.	N/A
**818C**	Long-eared owl	*Asio otus*	3427 Utzenstorf	BE	Choana	neg.	n.d.	n.d.	N/A
**818K**	Long-eared owl	*Asio otus*	3427 Utzenstorf	BE	Cloaca	neg.	n.d.	n.d.	N/A
**819C**	Carrion crow	*Corvus corone*	3432 Lützelflüh	BE	Choana	neg.	n.d.	n.d.	N/A
**819K**	Carrion crow	*Corvus corone*	3432 Lützelflüh	BE	Cloaca	neg.	n.d.	n.d.	N/A
**820C**	Eurasian hobby	*Falco subbuteo*	4573 Lohn-Ammansegg	SO	Choana	neg.	n.d.	n.d.	N/A
**820K**	Eurasian hobby	*Falco subbuteo*	4573 Lohn-Ammansegg	SO	Cloaca	neg.	n.d.	n.d.	N/A
**821C**	Black kite	*Milvus migrans*	3603 Uetendorf	BE	Choana	neg.	n.d.	n.d.	N/A
**821K**	Black kite	*Milvus migrans*	3603 Uetendorf	BE	Cloaca	neg.	n.d.	n.d.	N/A
**824C**	Carrion crow	*Corvus corone*	Zürich Flughafen	ZH	Choana	neg.	n.d.	n.d.	N/A
**824K**	Carrion crow	*Corvus corone*	Zürich Flughafen	ZH	Cloaca	neg.	n.d.	n.d.	N/A
**825C**	Carrion crow	*Corvus corone*	Zürich Flughafen	ZH	Choana	neg.	n.d.	n.d.	N/A

**825K**	Carrion crow	*Corvus corone*	Zürich Flughafen	ZH	Cloaca	neg.	n.d.	n.d.	N/A
**826C**	Carrion crow	*Corvus corone*	Zürich Flughafen	ZH	Choana	pos. (29.3)	neg.	neg.	*C. psittaci* 1V
**826K**	Carrion crow	*Corvus corone*	Zürich Flughafen	ZH	Cloaca	neg.	n.d.	n.d.	N/A
**827C**	Carrion crow	*Corvus corone*	Zürich Flughafen	ZH	Choana	neg.	n.d.	n.d.	N/A
**827K**	Carrion crow	*Corvus corone*	Zürich Flughafen	ZH	Cloaca	neg.	n.d.	n.d.	N/A
**828C**	Carrion crow	*Corvus corone*	Zürich Flughafen	ZH	Choana	neg.	n.d.	n.d.	N/A
**828K**	Carrion crow	*Corvus corone*	Zürich Flughafen	ZH	Cloaca	neg.	n.d.	n.d.	N/A
**829C**	Carrion crow	*Corvus corone*	Zürich Flughafen	ZH	Choana	neg.	n.d.	n.d.	N/A
**829K**	Carrion crow	*Corvus corone*	Zürich Flughafen	ZH	Cloaca	neg.	n.d.	n.d.	N/A
**830C**	Carrion crow	*Corvus corone*	Zürich Flughafen	ZH	Choana	neg.	n.d.	n.d.	N/A
**830K**	Carrion crow	*Corvus corone*	Zürich Flughafen	ZH	Cloaca	neg.	n.d.	n.d.	N/A
**831C**	Carrion crow	*Corvus corone*	Zürich Flughafen	ZH	Choana	neg.	n.d.	n.d.	N/A
**831K**	Carrion crow	*Corvus corone*	Zürich Flughafen	ZH	Cloaca	neg.	n.d.	n.d.	N/A
**832C**	Carrion crow	*Corvus corone*	Zürich Flughafen	ZH	Choana	neg.	n.d.	n.d.	N/A
**832K**	Carrion crow	*Corvus corone*	Zürich Flughafen	ZH	Cloaca	neg.	n.d.	n.d.	N/A
**833C**	Carrion crow	*Corvus corone*	Zürich Flughafen	ZH	Choana	neg.	n.d.	n.d.	N/A
**833K**	Carrion crow	*Corvus corone*	Zürich Flughafen	ZH	Cloaca	neg.	n.d.	n.d.	N/A
**834C**	Carrion crow	*Corvus corone*	Zürich Flughafen	ZH	Choana	neg.	n.d.	n.d.	N/A
**834K**	Carrion crow	*Corvus corone*	Zürich Flughafen	ZH	Cloaca	neg.	n.d.	n.d.	N/A
**835C**	Carrion crow	*Corvus corone*	Zürich Flughafen	ZH	Choana	neg.	n.d.	n.d.	N/A
**835K**	Carrion crow	*Corvus corone*	Zürich Flughafen	ZH	Cloaca	neg.	n.d.	n.d.	N/A
**836C**	Carrion crow	*Corvus corone*	Zürich Flughafen	ZH	Choana	neg.	n.d.	n.d.	N/A
**836K**	Carrion crow	*Corvus corone*	Zürich Flughafen	ZH	Cloaca	neg.	n.d.	n.d.	N/A
**837C**	Carrion crow	*Corvus corone*	Zürich Flughafen	ZH	Choana	neg.	n.d.	n.d.	N/A
**837K**	Carrion crow	*Corvus corone*	Zürich Flughafen	ZH	Cloaca	neg.	n.d.	n.d.	N/A
**838C**	Carrion crow	*Corvus corone*	Zürich Flughafen	ZH	Choana	neg.	n.d.	n.d.	N/A
**838K**	Carrion crow	*Corvus corone*	Zürich Flughafen	ZH	Cloaca	neg.	n.d.	n.d.	N/A
**839C**	Carrion crow	*Corvus corone*	Zürich Flughafen	ZH	Choana	neg.	n.d.	n.d.	N/A
**839K**	Carrion crow	*Corvus corone*	Zürich Flughafen	ZH	Cloaca	neg.	n.d.	n.d.	N/A
**840C**	Carrion crow	*Corvus corone*	Zürich Flughafen	ZH	Choana	neg.	n.d.	n.d.	N/A
**840K**	Carrion crow	*Corvus corone*	Zürich Flughafen	ZH	Cloaca	neg.	n.d.	n.d.	N/A
**841C**	Carrion crow	*Corvus corone*	Zürich Flughafen	ZH	Choana	neg.	n.d.	n.d.	N/A
**841K**	Carrion crow	*Corvus corone*	Zürich Flughafen	ZH	Cloaca	neg.	n.d.	n.d.	N/A
**842C**	Carrion crow	*Corvus corone*	Zürich Flughafen	ZH	Choana	pos. (37.1)	neg.	neg.	Not identified
**842K**	Carrion crow	*Corvus corone*	Zürich Flughafen	ZH	Cloaca	neg.	n.d.	n.d.	N/A
**843C**	Carrion crow	*Corvus corone*	Zürich Flughafen	ZH	Choana	neg.	n.d.	n.d.	N/A
**843K**	Carrion crow	*Corvus corone*	Zürich Flughafen	ZH	Cloaca	neg.	n.d.	n.d.	N/A
**844C**	Carrion crow	*Corvus corone*	Zürich Flughafen	ZH	Choana	neg.	n.d.	n.d.	N/A
**844K**	Carrion crow	*Corvus corone*	Zürich Flughafen	ZH	Cloaca	neg.	n.d.	n.d.	N/A
**845C**	Carrion crow	*Corvus corone*	Zürich Flughafen	ZH	Choana	neg.	n.d.	n.d.	N/A
**845K**	Carrion crow	*Corvus corone*	Zürich Flughafen	ZH	Cloaca	neg.	n.d.	n.d.	N/A
**846C**	Carrion crow	*Corvus corone*	Zürich Flughafen	ZH	Choana	pos. (31.8)	neg.	neg.	*C. abortus*/*C. psittaci*
**846K**	Carrion crow	*Corvus corone*	Zürich Flughafen	ZH	Cloaca	pos. (37.7)	neg.	neg.	Not identified

**847C**	Carrion crow	*Corvus corone*	Zürich Flughafen	ZH	Choana	pos. (33.0)	neg.	neg.	*C. abortus*/*C. psittaci*
**847K**	Carrion crow	*Corvus corone*	Zürich Flughafen	ZH	Cloaca	neg.	n.d.	n.d.	N/A
**848C**	Carrion crow	*Corvus corone*	Zürich Flughafen	ZH	Choana	pos. (26.6)	neg.	neg.	*C. psittaci* D
**848K**	Carrion crow	*Corvus corone*	Zürich Flughafen	ZH	Cloaca	neg.	n.d.	n.d.	N/A
**849C**	Carrion crow	*Corvus corone*	Zürich Flughafen	ZH	Choana	pos. (39.1)	neg.	neg.	Not identified
**849K**	Carrion crow	*Corvus corone*	Zürich Flughafen	ZH	Cloaca	pos. (38.4)	neg.	neg.	Not identified
**850C**	Carrion crow	*Corvus corone*	Zürich Flughafen	ZH	Choana	pos. (28.5)	neg.	neg.	*C. abortus*/*C. psittaci*
**850K**	Carrion crow	*Corvus corone*	Zürich Flughafen	ZH	Cloaca	pos. (27.9)	neg.	neg.	*C. psittaci* 1V
**851C**	Carrion crow	*Corvus corone*	Zürich Flughafen	ZH	Choana	pos. (31.9)	neg.	neg.	*C. abortus*/*C. psittaci*
**851K**	Carrion crow	*Corvus corone*	Zürich Flughafen	ZH	Cloaca	neg.	n.d.	n.d.	N/A
**852C**	Carrion crow	*Corvus corone*	Zürich Flughafen	ZH	Choana	neg.	n.d.	n.d.	N/A
**852K**	Carrion crow	*Corvus corone*	Zürich Flughafen	ZH	Cloaca	neg.	n.d.	n.d.	N/A
**853C**	Carrion crow	*Corvus corone*	Zürich Flughafen	ZH	Choana	pos. (34.6)	neg.	neg.	Not identified
**853K**	Carrion crow	*Corvus corone*	Zürich Flughafen	ZH	Cloaca	neg.	n.d.	n.d.	N/A
**854C**	Carrion crow	*Corvus corone*	Zürich Flughafen	ZH	Choana	neg.	n.d.	n.d.	N/A
**854K**	Carrion crow	*Corvus corone*	Zürich Flughafen	ZH	Cloaca	neg.	n.d.	n.d.	N/A
**855C**	Eurasian sparrowhawk	*Accipiter nisus*	Zürich Flughafen	ZH	Choana	neg.	n.d.	n.d.	N/A
**855K**	Eurasian sparrowhawk	*Accipiter nisus*	Zürich Flughafen	ZH	Cloaca	neg.	n.d.	n.d.	N/A
**856C**	Carrion crow	*Corvus corone*	Zürich Flughafen	ZH	Choana	pos. (29.3)	neg.	neg.	*C. psittaci* 1V
**856K**	Carrion crow	*Corvus corone*	Zürich Flughafen	ZH	Cloaca	pos. (42.5)	neg.	neg.	Not identified
**857C**	Carrion crow	*Corvus corone*	Zürich Flughafen	ZH	Choana	pos. (39.6)	neg.	neg.	Not identified
**857K**	Carrion crow	*Corvus corone*	Zürich Flughafen	ZH	Cloaca	pos. (33.9)	neg.	neg.	Not identified
**858C**	Carrion crow	*Corvus corone*	Zürich Flughafen	ZH	Choana	pos. (27.8)	neg.	neg.	*C. psittaci* 1V
**858K**	Carrion crow	*Corvus corone*	Zürich Flughafen	ZH	Cloaca	pos. (32.3)	neg.	neg.	*C. abortus*/*C. psittaci*
**859C**	Common kestrel	*Falco tinnunculus*	Zürich Flughafen	ZH	Choana	neg.	n.d.	n.d.	N/A
**859K**	Common kestrel	*Falco tinnunculus*	Zürich Flughafen	ZH	Cloaca	neg.	n.d.	n.d.	N/A
**860C**	Long-eared owl	*Asio otus*	6042 Dietwil	AG	Choana	neg.	n.d.	n.d.	N/A
**860K**	Long-eared owl	*Asio otus*	6042 Dietwil	AG	Cloaca	neg.	n.d.	n.d.	N/A
**861C**	Carrion crow	*Corvus corone*	Unknown	N/A	Choana	pos. (22.4)	neg.	neg.	*C. abortus*/*C. psittaci*
**861K**	Carrion crow	*Corvus corone*	Unknown	N/A	Cloaca	pos. (23.6)	neg.	neg.	*C. psittaci* 1V
**862C**	Eurasian jay	*Garrulus glandarius*	Unknown	N/A	Choana	neg.	n.d.	n.d.	N/A
**862K**	Eurasian jay	*Garrulus glandarius*	Unknown	N/A	Cloaca	neg.	n.d.	n.d.	N/A
**863C**	Common buzzard	*Buteo buteo*	Unknown	N/A	Choana	neg.	n.d.	n.d.	N/A
**863K**	Common buzzard	*Buteo buteo*	Unknown	N/A	Cloaca	neg.	n.d.	n.d.	N/A
**864C**	Common buzzard	*Buteo buteo*	3474 Wynigen	BE	Choana	neg.	n.d.	n.d.	N/A
**864K**	Common buzzard	*Buteo buteo*	3474 Wynigen	BE	Cloaca	neg.	n.d.	n.d.	N/A
**865C**	Long-eared owl	*Asio otus*	6026 Rain	LU	Choana	neg.	n.d.	n.d.	N/A
**866C**	Carrion crow	*Corvus corone*	6023 Rothenburg	LU	Choana	neg.	n.d.	n.d.	N/A
**867C**	Common buzzard	*Buteo buteo*	Unknown	N/A	Choana	neg.	n.d.	n.d.	N/A
**867K**	Common buzzard	*Buteo buteo*	Unknown	N/A	Cloaca	neg.	n.d.	n.d.	N/A
**868C**	Common buzzard	*Buteo buteo*	Unknown	N/A	Choana	neg.	n.d.	n.d.	N/A
**868K**	Common buzzard	*Buteo buteo*	Unknown	N/A	Cloaca	neg.	n.d.	n.d.	N/A
**869C**	Common kestrel	*Falco tinnunculus*	Unknown	N/A	Choana	neg.	n.d.	n.d.	N/A

**869K**	Common kestrel	*Falco tinnunculus*	Unknown	N/A	Cloaca	neg.	n.d.	n.d.	N/A
**870C**	Common kestrel	*Falco tinnunculus*	Unknown	N/A	Choana	neg.	n.d.	n.d.	N/A
**870K**	Common kestrel	*Falco tinnunculus*	Unknown	N/A	Cloaca	neg.	n.d.	n.d.	N/A
**871C**	Common kestrel	*Falco tinnunculus*	3267 Seedorf	BE	Choana	neg.	n.d.	n.d.	N/A
**871K**	Common kestrel	*Falco tinnunculus*	3267 Seedorf	BE	Cloaca	neg.	n.d.	n.d.	N/A
**872C**	Eurasian sparrowhawk	*Accipiter nisus*	2542 Pieterlen	BE	Choana	neg.	n.d.	n.d.	N/A
**872K**	Eurasian sparrowhawk	*Accipiter nisus*	2542 Pieterlen	BE	Cloaca	neg.	n.d.	n.d.	N/A
**873C**	Common kestrel	*Falco tinnunculus*			Choana	neg.	n.d.	n.d.	N/A
**873K**	Common kestrel	*Falco tinnunculus*			Cloaca	neg.	n.d.	n.d.	N/A
**874C**	Tawny owl	*Strix aluco*	Unknown	N/A	Choana	neg.	n.d.	n.d.	N/A
**874K**	Tawny owl	*Strix aluco*	Unknown	N/A	Cloaca	neg.	n.d.	n.d.	N/A
**875C**	Eurasian sparrowhawk	*Accipiter nisus*	Unknown	N/A	Choana	neg.	n.d.	n.d.	N/A
**875K**	Eurasian sparrowhawk	*Accipiter nisus*	Unknown	N/A	Cloaca	neg.	n.d.	n.d.	N/A
**876C**	Tawny owl	*Strix aluco*	3412 Heimiswil	BE	Choana	neg.	n.d.	n.d.	N/A
**876K**	Tawny owl	*Strix aluco*	3412 Heimiswil	BE	Cloaca	neg.	n.d.	n.d.	N/A
**877C**	Long-eared owl	*Asio otus*	3360 Herzogenbuchsee	BE	Choana	pos. (34.0)	neg.	neg.	Not identified
**877K**	Long-eared owl	*Asio otus*	3360 Herzogenbuchsee	BE	Cloaca	pos. (31.3)	neg.	neg.	*C. psittaci* M56
**878C**	Common buzzard	*Buteo buteo*	3315 Bätterkinden	BE	Choana	neg.	n.d.	n.d.	N/A
**878K**	Common buzzard	*Buteo buteo*	3315 Bätterkinden	BE	Cloaca	neg.	n.d.	n.d.	N/A
**879C**	Common buzzard	*Buteo buteo*	4614 Hägendorf	SO	Choana	neg.	n.d.	n.d.	N/A
**879K**	Common buzzard	*Buteo buteo*	4614 Hägendorf	SO	Cloaca	neg.	n.d.	n.d.	N/A
**880C**	Common buzzard	*Buteo buteo*	3628 Uttigen	BE	Choana	neg.	n.d.	n.d.	N/A
**880K**	Common buzzard	*Buteo buteo*	3628 Uttigen	BE	Cloaca	neg.	n.d.	n.d.	N/A
**881C**	Common buzzard	*Buteo buteo*	3380 Wangen a.d. Aare	BE	Choana	neg.	n.d.	n.d.	N/A
**881K**	Common buzzard	*Buteo buteo*	3380 Wangen a.d. Aare	BE	Cloaca	neg.	n.d.	n.d.	N/A
**882C**	Carrion crow	*Corvus corone*	3007 Bern	BE	Choana	neg.	n.d.	n.d.	N/A
**882K**	Carrion crow	*Corvus corone*	3007 Bern	BE	Cloaca	neg.	n.d.	n.d.	N/A
**883C**	Carrion crow	*Corvus corone*	3366 Bollodingen	BE	Choana	neg.	n.d.	n.d.	N/A
**883K**	Carrion crow	*Corvus corone*	3366 Bollodingen	BE	Cloaca	neg.	n.d.	n.d.	N/A
**884C**	Eurasian sparrowhawk	*Accipiter nisus*	Unknown	N/A	Choana	neg.	n.d.	n.d.	N/A
**884K**	Eurasian sparrowhawk	*Accipiter nisus*	Unknown	N/A	Cloaca	neg.	n.d.	n.d.	N/A
**885C**	Common kestrel	*Falco tinnunculus*	3317 Mülchi	BE	Choana	neg.	n.d.	n.d.	N/A
**885K**	Common kestrel	*Falco tinnunculus*	3317 Mülchi	BE	Cloaca	neg.	n.d.	n.d.	N/A
**886C**	Barn owl	*Tyto alba*	4586 Kyburg-Buchegg	SO	Choana	neg.	n.d.	n.d.	N/A
**886K**	Barn owl	*Tyto alba*	4586 Kyburg-Buchegg	SO	Cloaca	neg.	n.d.	n.d.	N/A
**887C**	Common kestrel	*Falco tinnunculus*	4554 Etziken	SO	Choana	neg.	n.d.	n.d.	N/A
**887K**	Common kestrel	*Falco tinnunculus*	4554 Etziken	SO	Cloaca	neg.	n.d.	n.d.	N/A
**888C**	Common kestrel	*Falco tinnunculus*	4710 Balsthal	SO	Choana	neg.	n.d.	n.d.	N/A
**888K**	Common kestrel	*Falco tinnunculus*	4710 Balsthal	SO	Cloaca	neg.	n.d.	n.d.	N/A
**889C**	Eurasian sparrowhawk	*Accipiter nisus*	Unknown	N/A	Choana	neg.	n.d.	n.d.	N/A
**889K**	Eurasian sparrowhawk	*Accipiter nisus*	Unknown	N/A	Cloaca	neg.	n.d.	n.d.	N/A

**890C**	Eurasian sparrowhawk	*Accipiter nisus*	Unknown	N/A	Choana	neg.	n.d.	n.d.	N/A
**890K**	Eurasian sparrowhawk	*Accipiter nisus*	Unknown	N/A	Cloaca	neg.	n.d.	n.d.	N/A
**891C**	Barn owl	*Tyto alba*	3365 Seeberg	BE	Choana	neg.	n.d.	n.d.	N/A
**891K**	Barn owl	*Tyto alba*	3365 Seeberg	BE	Cloaca	neg.	n.d.	n.d.	N/A
**892C**	Long-eared owl	*Asio otus*	Unknown	N/A	Choana	neg.	n.d.	n.d.	N/A
**892K**	Long-eared owl	*Asio otus*	Unknown	N/A	Cloaca	neg.	n.d.	n.d.	N/A
**893C**	Eurasian sparrowhawk	*Accipiter nisus*	3428 Wiler	BE	Choana	neg.	n.d.	n.d.	N/A
**893K**	Eurasian sparrowhawk	*Accipiter nisus*	3428 Wiler	BE	Cloaca	neg.	n.d.	n.d.	N/A
**894C**	Long-eared owl	*Asio otus*	3251 Wengi	BE	Choana	neg.	n.d.	n.d.	N/A
**894K**	Long-eared owl	*Asio otus*	3251 Wengi	BE	Cloaca	neg.	n.d.	n.d.	N/A
**895C**	Common kestrel	*Falco tinnunculus*	Unknown	N/A	Choana	neg.	n.d.	n.d.	N/A
**895K**	Common kestrel	*Falco tinnunculus*	Unknown	N/A	Cloaca	neg.	n.d.	n.d.	N/A
**896C**	Common kestrel	*Falco tinnunculus*	3473 Alchenstorf	BE	Choana	neg.	n.d.	n.d.	N/A
**896K**	Common kestrel	*Falco tinnunculus*	3473 Alchenstorf	BE	Cloaca	neg.	n.d.	n.d.	N/A
**897C**	Common kestrel	*Falco tinnunculus*	Unknown	N/A	Choana	neg.	n.d.	n.d.	N/A
**897K**	Common kestrel	*Falco tinnunculus*	Unknown	N/A	Cloaca	neg.	n.d.	n.d.	N/A
**898C**	Common kestrel	*Falco tinnunculus*	3314 Schalunen	BE	Choana	neg.	n.d.	n.d.	N/A
**898K**	Common kestrel	*Falco tinnunculus*	3314 Schalunen	BE	Cloaca	neg.	n.d.	n.d.	N/A
**899C**	Common kestrel	*Falco tinnunculus*	3110 Münsingen	BE	Choana	neg.	n.d.	n.d.	N/A
**899K**	Common kestrel	*Falco tinnunculus*	3110 Münsingen	BE	Cloaca	neg.	n.d.	n.d.	N/A
**900C**	Common kestrel	*Falco tinnunculus*	3365 Seeberg	BE	Choana	neg.	n.d.	n.d.	N/A
**900K**	Common kestrel	*Falco tinnunculus*	3365 Seeberg	BE	Cloaca	neg.	n.d.	n.d.	N/A
**901C**	Common kestrel	*Falco tinnunculus*	Unknown	N/A	Choana	neg.	n.d.	n.d.	N/A
**901K**	Common kestrel	*Falco tinnunculus*	Unknown	N/A	Cloaca	neg.	n.d.	n.d.	N/A
**902C**	Common kestrel	*Falco tinnunculus*	3422 Kirchberg	BE	Choana	neg.	n.d.	n.d.	N/A
**902K**	Common kestrel	*Falco tinnunculus*	3422 Kirchberg	BE	Cloaca	neg.	n.d.	n.d.	N/A
**903C**	Common buzzard	*Buteo buteo*	3006 Bern	BE	Choana	neg.	n.d.	n.d.	N/A
**903K**	Common buzzard	*Buteo buteo*	3006 Bern	BE	Cloaca	neg.	n.d.	n.d.	N/A
**904C**	Barn owl	*Tyto alba*	3273 Kappelen	BE	Choana	neg.	n.d.	n.d.	N/A
**904K**	Barn owl	*Tyto alba*	3273 Kappelen	BE	Cloaca	neg.	n.d.	n.d.	N/A
**905C**	Common kestrel	*Falco tinnunculus*	5463 Wislikofen	AG	Choana	neg.	n.d.	n.d.	N/A
**905K**	Common kestrel	*Falco tinnunculus*	5463 Wislikofen	AG	Cloaca	neg.	n.d.	n.d.	N/A
**906C**	Eurasian sparrowhawk	*Accipiter nisus*	3400 Burgdorf	BE	Choana	neg.	n.d.	n.d.	N/A
**906K**	Eurasian sparrowhawk	*Accipiter nisus*	3400 Burgdorf	BE	Cloaca	neg.	n.d.	n.d.	N/A
**907C**	Common buzzard	*Buteo buteo*	3422 Kirchberg	BE	Choana	neg.	n.d.	n.d.	N/A
**907K**	Common buzzard	*Buteo buteo*	3422 Kirchberg	BE	Cloaca	neg.	n.d.	n.d.	N/A
**908C**	Common buzzard	*Buteo buteo*	4553 Subingen	SO	Choana	neg.	n.d.	n.d.	N/A
**908K**	Common buzzard	*Buteo buteo*	4553 Subingen	SO	Cloaca	neg.	n.d.	n.d.	N/A
**909C**	Red kite	*Milvus milvus*	3295 Rüti bei Büren	BE	Choana	neg.	n.d.	n.d.	N/A
**909K**	Red kite	*Milvus milvus*	3295 Rüti bei Büren	BE	Cloaca	neg.	n.d.	n.d.	N/A
**910C**	Common buzzard	*Buteo buteo*	Unknown	N/A	Choana	neg.	n.d.	n.d.	N/A
**910K**	Common buzzard	*Buteo buteo*	Unknown	N/A	Cloaca	pos. (41.2)	neg.	neg.	Not identified
**911C**	Eurasian sparrowhawk	*Accipiter nisus*	Unknown	N/A	Choana	neg.	n.d.	n.d.	N/A

**911K**	Eurasian sparrowhawk	*Accipiter nisus*	Unknown	N/A	Cloaca	neg.	n.d.	n.d.	N/A
**912C**	Common kestrel	*Falco tinnunculus*	Unknown	N/A	Choana	neg.	n.d.	n.d.	N/A
**912K**	Common kestrel	*Falco tinnunculus*	Unknown	N/A	Cloaca	neg.	n.d.	n.d.	N/A
**913C**	Common buzzard	*Buteo buteo*	4713 Matzendorf	SO	Choana	neg.	n.d.	n.d.	N/A
**913K**	Common buzzard	*Buteo buteo*	4713 Matzendorf	SO	Cloaca	neg.	n.d.	n.d.	N/A
**914C**	Common buzzard	*Buteo buteo*	Unknown	N/A	Choana	neg.	n.d.	n.d.	N/A
**914K**	Common buzzard	*Buteo buteo*	Unknown	N/A	Cloaca	neg.	n.d.	n.d.	N/A
**926C**	Bearded vulture	*Gypaetus barbatus*	7522 La Punt-Chamues-ch	GR	Choana	neg.	n.d.	n.d.	N/A
**926K**	Bearded vulture	*Gypaetus barbatus*	7522 La Punt-Chamues-ch	GR	Cloaca	neg.	n.d.	n.d.	N/A
**927C**	Golden eagle	*Aquila chrysaetos*	7463 Surses	GR	Choana	neg.	n.d.	n.d.	N/A
**927K**	Golden eagle	*Aquila chrysaetos*	7463 Surses	GR	Cloaca	neg.	n.d.	n.d.	N/A
**928C**	Golden eagle	*Aquila chrysaetos*	7165 Breil	GR	Choana	neg.	n.d.	n.d.	N/A
**928K**	Golden eagle	*Aquila chrysaetos*	7165 Breil	GR	Cloaca	neg.	n.d.	n.d.	N/A
**929K**	Golden eagle	*Aquila chrysaetos*	7156 Pigniu	GR	Cloaca	neg.	n.d.	n.d.	N/A
**930C**	Golden eagle	*Aquila chrysaetos*	7608 Castasegna	GR	Choana	neg.	n.d.	n.d.	N/A
**930K**	Golden eagle	*Aquila chrysaetos*	7608 Castasegna	GR	Cloaca	neg.	n.d.	n.d.	N/A
**931C**	Eurasian eagle-owl	*Asio otus*	7304 Maienfeld	GR	Choana	neg.	n.d.	n.d.	N/A
**931K**	Eurasian eagle-owl	*Asio otus*	7304 Maienfeld	GR	Cloaca	neg.	n.d.	n.d.	N/A
**932C**	Eurasian eagle-owl	*Asio otus*	6565 Lumbreida San Bernardino	GR	Choana	neg.	n.d.	n.d.	N/A
**932K**	Eurasian eagle-owl	*Asio otus*	6565 Lumbreida San Bernardino	GR	Cloaca	neg.	n.d.	n.d.	N/A
**933K**	Golden eagle	*Aquila chrysaetos*	6534 San Vittore	GR	Cloaca	neg.	n.d.	n.d.	N/A
**934C**	Eurasian eagle-owl	*Asio otus*	8752 Näfels	GL	Choana	neg.	n.d.	n.d.	N/A
**934K**	Eurasian eagle-owl	*Asio otus*	8752 Näfels	GL	Cloaca	neg.	n.d.	n.d.	N/A
**935C**	Hooded crow	*Corvus cornix*	7130 Ilanz/Glion	GR	Choana	neg.	n.d.	n.d.	N/A
**935K**	Hooded crow	*Corvus cornix*	7130 Ilanz/Glion	GR	Cloaca	neg.	n.d.	n.d.	N/A
**945C**	Carrion crow	*Corvus corone*	1294 Genthod	GE	Choana	neg.	n.d.	n.d.	N/A
**945K**	Carrion crow	*Corvus corone*	1294 Genthod	GE	Cloaca	neg.	n.d.	n.d.	N/A
**946C**	Carrion crow	*Corvus corone*	1294 Genthod	GE	Choana	neg.	n.d.	n.d.	N/A
**946K**	Carrion crow	*Corvus corone*	1294 Genthod	GE	Cloaca	neg.	n.d.	n.d.	N/A
**947C**	Carrion crow	*Corvus corone*	1294 Genthod	GE	Choana	neg.	n.d.	n.d.	N/A
**947K**	Carrion crow	*Corvus corone*	1294 Genthod	GE	Cloaca	neg.	n.d.	n.d.	N/A
**948C**	Carrion crow	*Corvus corone*	1294 Genthod	GE	Choana	neg.	n.d.	n.d.	N/A
**948K**	Carrion crow	*Corvus corone*	1294 Genthod	GE	Cloaca	neg.	n.d.	n.d.	N/A
**949C**	Carrion crow	*Corvus corone*	1294 Genthod	GE	Choana	neg.	n.d.	n.d.	N/A
**949K**	Carrion crow	*Corvus corone*	1294 Genthod	GE	Cloaca	neg.	n.d.	n.d.	N/A
**950C**	Carrion crow	*Corvus corone*	1294 Genthod	GE	Choana	neg.	n.d.	n.d.	N/A
**950K**	Carrion crow	*Corvus corone*	1294 Genthod	GE	Cloaca	neg.	n.d.	n.d.	N/A
**951C**	Carrion crow	*Corvus corone*	1294 Genthod	GE	Choana	neg.	n.d.	n.d.	N/A
**951K**	Carrion crow	*Corvus corone*	1294 Genthod	GE	Cloaca	neg.	n.d.	n.d.	N/A

**952C**	Carrion crow	*Corvus corone*	1294 Genthod	GE	Choana	neg.	n.d.	n.d.	N/A
**952K**	Carrion crow	*Corvus corone*	1294 Genthod	GE	Cloaca	neg.	n.d.	n.d.	N/A
**953C**	Carrion crow	*Corvus corone*	1294 Genthod	GE	Choana	neg.	n.d.	n.d.	N/A
**953K**	Carrion crow	*Corvus corone*	1294 Genthod	GE	Cloaca	neg.	n.d.	n.d.	N/A
**954C**	Carrion crow	*Corvus corone*	1294 Genthod	GE	Choana	neg.	n.d.	n.d.	N/A
**954K**	Carrion crow	*Corvus corone*	1294 Genthod	GE	Cloaca	neg.	n.d.	n.d.	N/A
**955C**	Carrion crow	*Corvus corone*	1294 Genthod	GE	Choana	neg.	n.d.	n.d.	N/A
**955K**	Carrion crow	*Corvus corone*	1294 Genthod	GE	Cloaca	neg.	n.d.	n.d.	N/A
**956C**	Carrion crow	*Corvus corone*	1294 Genthod	GE	Choana	neg.	n.d.	n.d.	N/A
**956K**	Carrion crow	*Corvus corone*	1294 Genthod	GE	Cloaca	neg.	n.d.	n.d.	N/A
**957C**	Carrion crow	*Corvus corone*	1294 Genthod	GE	Choana	neg.	n.d.	n.d.	N/A
**957K**	Carrion crow	*Corvus corone*	1294 Genthod	GE	Cloaca	neg.	n.d.	n.d.	N/A
**958C**	Carrion crow	*Corvus corone*	1294 Genthod	GE	Choana	neg.	n.d.	n.d.	N/A
**958K**	Carrion crow	*Corvus corone*	1294 Genthod	GE	Cloaca	neg.	n.d.	n.d.	N/A
**959C**	Carrion crow	*Corvus corone*	1294 Genthod	GE	Choana	neg.	n.d.	n.d.	N/A
**959K**	Carrion crow	*Corvus corone*	1294 Genthod	GE	Cloaca	neg.	n.d.	n.d.	N/A
**960C**	Carrion crow	*Corvus corone*	1786 Sugiez	FR	Choana	neg.	n.d.	n.d.	N/A
**960K**	Carrion crow	*Corvus corone*	1786 Sugiez	FR	Cloaca	neg.	n.d.	n.d.	N/A
**961K**	Carrion crow	*Corvus corone*	1786 Sugiez	FR	Cloaca	neg.	n.d.	n.d.	N/A
**962C**	Carrion crow	*Corvus corone*	1786 Sugiez	FR	Choana	neg.	n.d.	n.d.	N/A
**963C**	Carrion crow	*Corvus corone*	1786 Sugiez	FR	Choana	neg.	n.d.	n.d.	N/A
**964C**	Carrion crow	*Corvus corone*	1786 Sugiez	FR	Choana	neg.	n.d.	n.d.	N/A
**964K**	Carrion crow	*Corvus corone*	1786 Sugiez	FR	Cloaca	neg.	n.d.	n.d.	N/A
**965C**	Carrion crow	*Corvus corone*	1786 Sugiez	FR	Choana	neg.	n.d.	n.d.	N/A
**965K**	Carrion crow	*Corvus corone*	1786 Sugiez	FR	Cloaca	neg.	n.d.	n.d.	N/A
**966C**	Carrion crow	*Corvus corone*	1786 Sugiez	FR	Choana	neg.	n.d.	n.d.	N/A
**967C**	Carrion crow	*Corvus corone*	1786 Sugiez	FR	Choana	neg.	n.d.	n.d.	N/A
**967K**	Carrion crow	*Corvus corone*	1786 Sugiez	FR	Cloaca	neg.	n.d.	n.d.	N/A
**968C**	Carrion crow	*Corvus corone*	1786 Sugiez	FR	Choana	neg.	n.d.	n.d.	N/A
**968K**	Carrion crow	*Corvus corone*	1786 Sugiez	FR	Cloaca	neg.	n.d.	n.d.	N/A
**969C**	Carrion crow	*Corvus corone*	1786 Sugiez	FR	Choana	neg.	n.d.	n.d.	N/A
**969K**	Carrion crow	*Corvus corone*	1786 Sugiez	FR	Cloaca	neg.	n.d.	n.d.	N/A
**970C**	Carrion crow	*Corvus corone*	1786 Sugiez	FR	Choana	neg.	n.d.	n.d.	N/A
**970K**	Carrion crow	*Corvus corone*	1786 Sugiez	FR	Cloaca	neg.	n.d.	n.d.	N/A
**971C**	Carrion crow	*Corvus corone*	1786 Sugiez	FR	Choana	neg.	n.d.	n.d.	N/A
**971K**	Carrion crow	*Corvus corone*	1786 Sugiez	FR	Cloaca	neg.	n.d.	n.d.	N/A
**972C**	Carrion crow	*Corvus corone*	1786 Sugiez	FR	Choana	pos. (27.5)	neg.	neg.	C. psittaci 1V
**972K**	Carrion crow	*Corvus corone*	1786 Sugiez	FR	Cloaca	pos. (36.2)	neg.	neg.	Not identified
**973C**	Eurasian sparrowhawk	*Accipiter nisus*	6703 Riviera	TI	Choana	neg.	n.d.	n.d.	N/A
**973K**	Eurasian sparrowhawk	*Accipiter nisus*	6703 Riviera	TI	Cloaca	neg.	n.d.	n.d.	N/A
**974C**	Eurasian sparrowhawk	*Accipiter nisus*	Unknown	N/A	Choana	neg.	n.d.	n.d.	N/A
**974K**	Eurasian sparrowhawk	*Accipiter nisus*	Unknown	N/A	Cloaca	neg.	n.d.	n.d.	N/A
**975C**	Northern goshawk	*Accipiter gentilis*	6702 Claro	TI	Choana	neg.	n.d.	n.d.	N/A

**975K**	Northern goshawk	*Accipiter gentilis*	6702 Claro	TI	Cloaca	neg.	n.d.	n.d.	N/A
**976C**	Eurasian sparrowhawk	*Accipiter nisus*	6710 Biasca	TI	Choana	neg.	n.d.	n.d.	N/A
**976K**	Eurasian sparrowhawk	*Accipiter nisus*	6710 Biasca	TI	Cloaca	neg.	n.d.	n.d.	N/A
**977C**	Common buzzard	*Buteo buteo*	Unknown	N/A	Choana	neg.	n.d.	n.d.	N/A
**977K**	Common buzzard	*Buteo buteo*	Unknown	N/A	Cloaca	neg.	n.d.	n.d.	N/A
**978C**	European Honey-buzzard	*Pernis apivorus*	6514 Sementina	TI	Choana	neg.	n.d.	n.d.	N/A
**978K**	European Honey-buzzard	*Pernis apivorus*	6514 Sementina	TI	Cloaca	neg.	n.d.	n.d.	N/A
**979C**	Tawny owl	*Strix aluco*	6760 Faido	TI	Choana	neg.	n.d.	n.d.	N/A
**979K**	Tawny owl	*Strix aluco*	6760 Faido	TI	Cloaca	neg.	n.d.	n.d.	N/A
**980C**	Common kestrel	*Falco tinnunculus*	6535 Roveredo	GR	Choana	neg.	n.d.	n.d.	N/A
**980K**	Common kestrel	*Falco tinnunculus*	6535 Roveredo	GR	Cloaca	neg.	n.d.	n.d.	N/A
**981C**	Peregrine falcon	*Falco peregrinus*	6512 Giubiasco	TI	Choana	neg.	n.d.	n.d.	N/A
**981K**	Peregrine falcon	*Falco peregrinus*	6512 Giubiasco	TI	Cloaca	neg.	n.d.	n.d.	N/A
**982C**	Eurasian jay	*Garrulus glandarius*	3995 Ernen	VS	Choana	neg.	n.d.	n.d.	N/A
**982K**	Eurasian jay	*Garrulus glandarius*	3995 Ernen	VS	Cloaca	neg.	n.d.	n.d.	N/A
**983C**	Eurasian jay	*Garrulus glandarius*	3910 Saas Grund	VS	Choana	neg.	n.d.	n.d.	N/A
**983K**	Eurasian jay	*Garrulus glandarius*	3910 Saas Grund	VS	Cloaca	neg.	n.d.	n.d.	N/A
**984K**	Golden eagle	*Aquila chrysaetos*	3911 Ried Brig	VS	Cloaca	neg.	n.d.	n.d.	N/A
**985C**	Northern goshawk	*Accipiter gentilis*	3984 Fiesch	VS	Choana	neg.	n.d.	n.d.	N/A
**985K**	Northern goshawk	*Accipiter gentilis*	3984 Fiesch	VS	Cloaca	neg.	n.d.	n.d.	N/A
**986C**	Carrion crow	*Corvus corone*	3995 Ernen	VS	Choana	neg.	n.d.	n.d.	N/A
**987C**	Eurasian jay	*Garrulus glandarius*	3911 Ried Brig	VS	Choana	neg.	n.d.	n.d.	N/A
**987K**	Eurasian jay	*Garrulus glandarius*	3911 Ried Brig	VS	Cloaca	neg.	n.d.	n.d.	N/A
**988C**	Eurasian jay	*Garrulus glandarius*	3911 Ried Brig	VS	Choana	neg.	n.d.	n.d.	N/A
**989C**	Eurasian sparrowhawk	*Accipiter nisus*	3984 Fiesch	VS	Choana	neg.	n.d.	n.d.	N/A
**989K**	Eurasian sparrowhawk	*Accipiter nisus*	3984 Fiesch	VS	Cloaca	neg.	n.d.	n.d.	N/A
**990C**	Carrion crow	*Corvus corone*	3916 Ferden	VS	Choana	neg.	n.d.	n.d.	N/A
**990K**	Carrion crow	*Corvus corone*	3916 Ferden	VS	Cloaca	neg.	n.d.	n.d.	N/A
**991C**	Eurasian magpie	*Pica pica*	3911 Ried Brig	VS	Choana	neg.	n.d.	n.d.	N/A
**991K**	Eurasian magpie	*Pica pica*	3911 Ried Brig	VS	Cloaca	neg.	n.d.	n.d.	N/A
**992C**	Eurasian jay	*Garrulus glandarius*	3995 Ernen	VS	Choana	neg.	n.d.	n.d.	N/A
**992K**	Eurasian jay	*Garrulus glandarius*	3995 Ernen	VS	Cloaca	neg.	n.d.	n.d.	N/A
**993K**	Carrion crow	*Corvus corone*	1786 Sugiez	FR	Cloaca	neg.	n.d.	n.d.	N/A
